# Albumin/vaccine nanocomplexes that assemble in vivo for combination cancer immunotherapy

**DOI:** 10.1038/s41467-017-02191-y

**Published:** 2017-12-05

**Authors:** Guizhi Zhu, Geoffrey M. Lynn, Orit Jacobson, Kai Chen, Yi Liu, Huimin Zhang, Ying Ma, Fuwu Zhang, Rui Tian, Qianqian Ni, Siyuan Cheng, Zhantong Wang, Nan Lu, Bryant C. Yung, Zhe Wang, Lixin Lang, Xiao Fu, Albert Jin, Ido D. Weiss, Harshad Vishwasrao, Gang Niu, Hari Shroff, Dennis M. Klinman, Robert A. Seder, Xiaoyuan Chen

**Affiliations:** 1 Laboratory of Molecular Imaging and Nanomedicine, National Institute of Biomedical Imaging and Bioengineering (NIBIB), National Institutes of Health (NIH), Bethesda, MD 20892 USA; 2 Vaccine Research Center, National Institute of Allergy and Infectious Diseases (NIAID), NIH, Bethesda, MD 20892 USA; 3 Molecular Imaging Center, Department of Radiology, Keck School of Medicine, University of Southern California, Los Angeles, CA 90033 USA; 4 School of Engineering, China Pharmaceutical University, Nanjing, 210009 China; 5 State Key Laboratory of Molecular Vaccinology and Molecular Diagnostics, Center for Molecular Imaging and Translational Medicine, School of Public Health, Xiamen University, Xiamen, 361102 China; 6 Laboratory of Cellular Imaging and Macromolecular Biophysics, NIBIB, NIH, Bethesda, MD 20892 USA; 7 Laboratory of Molecular Immunology, NIAID, NIH, Bethesda, MD 20892 USA; 8 Advanced Imaging and Microscopy Resource, National Institutes of Health, Bethesda, 20892 MD USA; 9 Section on High Resolution Optical Imaging, NIBIB, NIH, Bethesda, MD 20892 USA; 10 Cancer and Inflammation Program, National Cancer Institute, Frederick, MD 21702 USA

## Abstract

Subunit vaccines have been investigated in over 1000 clinical trials of cancer immunotherapy, but have shown limited efficacy. Nanovaccines may improve efficacy but have rarely been clinically translated. By conjugating molecular vaccines with Evans blue (EB) into albumin-binding vaccines (AlbiVax), here we develop clinically promising albumin/AlbiVax nanocomplexes that self-assemble in vivo from AlbiVax and endogenous albumin for efficient vaccine delivery and potent cancer immunotherapy. PET pharmacoimaging, super-resolution microscopies, and flow cytometry reveal almost 100-fold more efficient co-delivery of CpG and antigens (Ags) to lymph nodes (LNs) by albumin/AlbiVax than benchmark incomplete Freund’s adjuvant (IFA). Albumin/AlbiVax elicits ~10 times more frequent peripheral antigen-specific CD8^+^ cytotoxic T lymphocytes with immune memory than IFA-emulsifying vaccines. Albumin/AlbiVax specifically inhibits progression of established primary or metastatic EG7.OVA, B16F10, and MC38 tumors; combination with anti-PD-1 and/or Abraxane further potentiates immunotherapy and eradicates most MC38 tumors. Albumin/AlbiVax nanocomplexes are thus a robust platform for combination cancer immunotherapy.

## Abstract (web summary)

Albumin conjugates can enhance drug delivery. Here, the authors repurpose albumin-binding Evans blue to develop nanovaccines that co-deliver adjuvants and tumor neoantigens to antigen-presenting cells in lymph nodes, resulting in potent and durable antitumour immunity in combination immunotherapy.

## Introduction

The past decade has witnessed remarkable advances in cancer immunotherapy, including immune checkpoint inhibitors^1^. However, only limited patient populations respond to single immune checkpoint inhibitors, and although combining multiple biologic checkpoint inhibitors increases response rates, it also elevates toxicity. Vaccines exploit synergistic signaling pathways for combination cancer immunotherapy. Despite the tremendous potential of subunit vaccines for cancer immunotherapy, their clinical outcome thus far has been suboptimal, largely due to inefficient co-delivery of adjuvants and Ags to secondary lymphoid organs, such as LNs where immune responses of lymphocytes are coordinated, leading to weak immunostimulation and immune tolerance^2,3^. Although tumor-specific neoantigens are promising for personalized immunotherapy^4–9^, there remains a lack of a general technology to deliver heterogenous peptide neoantigens efficiently. As a clinical benchmark, subunit Ags are administered in depot-forming water-in-oil emulsions (e.g., IFA) of CpG oligodeoxynucleotide, a Toll-like receptor 9 (TLR9) agonist, and T helper 1 enhancer^10,11^. Despite sustainable vaccine release from the depot and improved Ag immunogenicity, IFA has limited clinical efficacy. Indeed, IFA sequesters minimal Ag determinant-specific T cells in the depots, exhausting and depleting T cells, thereby preventing T cells from infiltrating tumors^12^. Alternatively, nanovaccines, which can efficiently co-deliver locally administered adjuvants/Ags into LNs via lymphatics, have been explored for vaccine delivery^13–16^. However, the pharmacological behaviors of perhaps all currently reported nanovaccines were studied either invasively or semiquantitatively, and more importantly, the clinical translation of most synthetic nanovaccines has been hampered by complications in large-scale manufacturing, quality control, formulation, and safety^17,18^.

We hypothesize that nanovaccines assembled in vivo from exogenous molecular vaccines and endogenous nanocarriers would not only enhance vaccine bioavailability in LNs, but also bypass the complications mentioned above. Compared with synthetic nanovaccines, molecular vaccines are chemically defined and often relatively well suited to large-scale production, including quality control and safety evaluation. Many natural biomolecules, such as albumin and immunoglobins (IgGs), are excellent candidates for endogenous carriers, given their (1) long half-lives (over 20 days in humans)^19^, (2) ubiquity in peripheral tissues, and (3) easy accessibility to vaccines with good patient compliance^20^. Internalized IgG and albumin in cells can be recycled from endolysosomes and transported out of cells via acidic binding to receptors, including fragment crystallizable receptor (FcR) and neonatal Fc receptor (FcRn), thereby avoiding protein degradation and prolonging their half-lives. We chose albumin as an endogenous component to assemble nanovaccines with exogenous molecular vaccines, for the following reasons: (1) While binding to endogenous IgG Fc likely interferes with IgG’s biological functions, albumin is a natural carrier with multiple, versatile, intrinsic-binding sites for biomolecules, and drugs^21–25^; (2) The size of murine and human albumins (66 kDa) exceeds the cutoff (45 kDa) to be disseminated systemically from interstitial space by blood^26^, meaning that nearly all will be drained through lymphatics to LNs. Together with the slow lymph flow, these characteristics offer a long time window for albumin/AlbiVax nanocomplexes to modulate lymphocytes in LNs. Interestingly, albumin can be efficiently internalized by antigen-presenting cells (APCs) via endocytosis, which can facilitate intracellular vaccine delivery for optimal Ag processing and presentation. Indeed, albumin–drug conjugates/complexes have been enthusiastically pursued for decades^27^, ranging from albumin conjugates of antiviral 5-fluorodeoxyuridine and cytosine arabinoside^22,25,28^, lipid-prodrug conjugates^29^, drug-peptide/fatty acid conjugates^30^, to Abraxane of albumin/paclitaxel nanocomplexes. Specific albumin–vaccine conjugates include streptococcal protein G-antigen conjugates^31^, albumin–antigen/interleukin-2 (IL-2) fusion proteins^25,28^, and lipid–vaccine conjugates^20,32^ that remarkably improved vaccine delivery to LNs and substantially potentiated immune responses.

Here we took a clinically oriented perspective to develop AlbiVax by first repurposing a clinically practiced EB that binds to albumin at binding site I^33^, and then conjugating molecular vaccines with an EB derivative. The resulting albumin/AlbiVax nanocomplexes that assemble in vivo were efficiently delivered to LNs and induced potent and durable anti-cancer immunity. EB and derivatives have been developed for preclinical and clinical LN identification^34–39^. They have excellent clinical safety profiles^36,39^, and rhesus monkeys survived 25 mg kg^−1^ systemically injected EB^40^, a dose 500-fold higher than that of AlbiVax. We exploited multiscale pharmacoimaging to quantitatively and systematically investigate the pharmacology of albumin/AlbiVax nanocomplexes in animals by PET, in entire LNs by light sheet fluorescence microscopy, and in APCs by super-resolution instantaneous structured illumination microscopy (instant SIM)^41^. Albumin/AlbiVax nanocomplexes potentiated both innate and adaptive immunity, leading to a markedly expanded repertoire of Ag-specific CD8^+^ CTLs and T cell memory. Albumin/AlbiVax nanocomplexes, alone or in combination with anti-PD-1 and/or Abraxane, dramatically inhibited tumor progression in multiple syngeneic tumor models.

## Results

### Efficient LN delivery of albumin/AlbiVax nanocomplexes

**Fig. 1:**
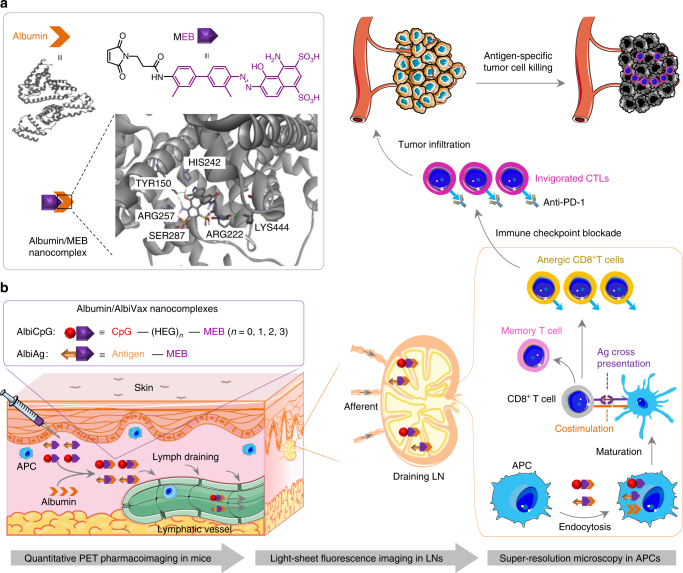
Schematic of albumin/AlbiVax nanocomplexes for efficient vaccine delivery and combination cancer immunotherapy. **a** Upper: structure of HSA (PDB ID: 2BXH) and chemical structure of MEB; lower: schematic structure of albumin/MEB nanocomplexes (left) and 3D molecular structure predicted by molecular docking (right). Sticks represent MEB and the amino acid residues in the binding site I of HSA. Green dashed lines represent hydrogen bonds between MEB and amino acids. **b** Working mechanism of albumin/AlbiVax nanocomplexes as potent T cell vaccines. Left box: modular structures of AlbiCpG and albumin-binding Ag (AlbiAg). AlbiCpG were engineered by site specifically conjugating MEB and thiol-modified CpG, with hexaethyloxy-glycol (HEG) as tunable linkers; AlbiAg was synthesized by conjugating MEB and cysteine-modified Ags, including TAA and tumor-specific neoantigen discovered via exome sequencing. Left lower: locally administered AlbiVax binds to endogenous albumin and assembles into albumin/AlbiVax nanocomplexes, which were efficiently delivered to LNs due to lymphatic drainage and prolonged retention in LNs. Right: harnessing the endocytosis pathway of albumin, albumin/AlbiCpG and albumin/AlbiAg nanocomplexes were co-delivered into APCs and activated APCs for antigen cross presentation and clonal expansion of antigen-specific CD8^+^ CTLs, thereby eliciting robust and durable antitumor immunity. While albumin/AlbiVax nanocomplexes upregulated the expression of PD-1 on these CD8^+^ CTLs, combination of albumin/AlbiVax nanocomplexes with anti-PD-1 dramatically enhanced immunotherapeutic efficacy in established primary and metastatic tumors. The pharmacological behaviors of albumin/AlbiVax nanocomplexes were studied by quantitative PET imaging, light sheet fluorescence microscopy in whole cleared tissue, and super-resolution imaging in single APCs

AlbiVax was synthesized by conjugating thiol-modified vaccines with maleimide-functionalized EB derivative (MEB), which tightly binds to human serum albumin (HSA) on binding site I (Fig. 1 and Supplementary Figs. 1 and 2). CpG was studied first. MEB was conjugated onto the 3′-end of CpG (Supplementary Fig. 3), and the resulting MEB–CpG, as a candidate of albumin-binding CpG (AlbiCpG), either alone or premixed with albumin, maintained the immunostimulatory activity of CpG in RAW264.7 macrophages. By contrast, MEB modification on the 5′-end of CpG abrogated its activity^42^. MEB-GpC control was also not immunostimulatory (Supplementary Fig. 4). Like MEB^43^, in the presence of albumin, the originally self-quenched MEB fluorescence of MEB–CpG was recovered and the fluorescence lifetime was prolonged, indicating albumin binding of MEB–CpG. The fluorescence of MEB–CpG was less than MEB upon albumin binding (Supplementary Fig. 5a, b). Intriguingly, the fluorescence of MEB–DNAs was partially activated in the absence of albumin, in a manner independent of DNA sequence, length, and deoxynucleotides, presumably resulting from intramolecular electrostatic interactions between MEB and ssDNA in MEB–DNAs, as well as reduced intermolecular stacking of MEB–DNAs relative to free MEB (Supplementary Figs. 6–9).

**Fig. 2:**
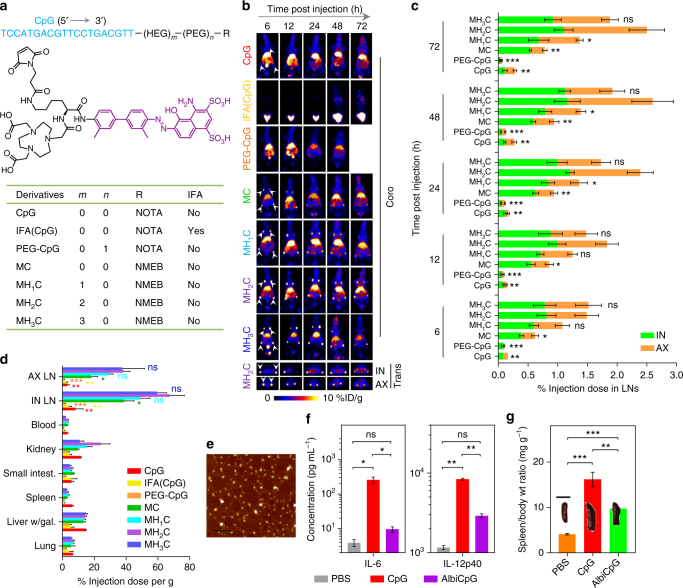
Quantitative screening of albumin/AlbiCpG nanocomplexes for LN delivery. **a** Structures, radiolabeling, and formulations of CpG derivatives for PET-based screening. Shown in the middle is the molecular structure of NMEB used for radiolabeling of AlbiCpG. Bioconjugations were conducted using thiol on Cp, maleimide on NMEB (for AlbiCpG), or NOTA (for CpG and IFA(CpG)), as well as maleimide and alkyne on bifunctional PEG20K and azide on NOTA for PEG–CpG. **b** Upper: representative coronal (coro) PET images showing FVB mice at 6 h post s.c. injection (dose: 4.4–5.5 Mbq) of CpG derivatives at tail base. Nanocomplexes of albumin with four MEB–CpG derivatives, respectively, efficiently delivered CpG to LNs, relative to free CpG, PEG–CpG, and IFA(CpG). Lower: representative transverse (trans) PET images showing LN delivery of MH_2_C at 6 h post injection. White arrow heads mark IN and AX LNs. **c** Amounts of CpG derivatives in IN and AX LNs quantified from three-dimensional (3D)-reconstructed, decay-corrected PET images (*n = *4–8). The IFA(CpG) signal in LNs was too low to visualize for quantification. **d** Biodistribution of tested compounds in resected organs measured by γ counting 3 days post injection (*n = *4). (Liver/gal: liver with gallbladder; intest.: intestine). **e** An AFM image of HSA/AlbiCpG nanocomplexes (premixed AlbiCpG: HSA = 1:1). Scale bar: 200 nm. **f**, **g** In C57BL/6 mice, AlbiCpG s.c. injected at the tail base ameliorated the systemic toxicity of CpG (*n = *4) as shown by lower IL-6 and IL-12p40 titers in blood (**f**) (dose: 5 nmol CpG equivalents) and ameliorated splenomegaly (**g**) on day 6 (dose: 5 nmol CpG equivalents on day 0 and day 3). Scale bar for spleen: 1 cm. Wt: weight. Data show mean ± s.e.m. of two–three independent experiments. ***p < 0.001, **p < 0.01, *p < 0.05, ns: not significant (*p* > 0.05) by one-way ANOVA with Bonferroni post test. Asterisks in **c** indicate statistically significant differences between the corresponding compounds with MH_2_C

The above fluorescence phenomena imply interactions between MEB and DNA in MEB–CpG, which likely interfere with the albumin binding of MEB–CpG. Thus, 0, 1, 2, and 3 units of HEG linkers between MEB and CpG were used to synthesize MEB–CpG (MC), MEB–HEG–CpG (MH_1_C), MEB–(HEG)_2_–CpG (MH_2_C), and MEB–(HEG)_3_–CpG (MH_3_C) with ca. 0, 2, 4, and 6-nm linkers, respectively, and quantitatively screened for LN delivery by PET in FVB mice. Specifically, MEB–CpG derivatives were radiolabeled with ^64^Cu (*t*_1/2_: 12.6 h) via 1,4,7-triazacyclononane-triacetic acid (NOTA)-MEB (NMEB)^44^ (Fig. 2a and Supplementary Fig. 10), and were subcutaneously (s.c.) injected (dose: 4.4–5.5 Mbq) at the tail base of mice, followed by PET imaging over 3 days (Fig. 2b, Supplementary Figs. 11 and 12, and Supplementary Movie 1). PEG–CpG conjugate (PEG MW: 20 kDa), and CpG with or without IFA were used as controls. As quantified from decay-corrected PET results (Fig. 2c), only <0.3% injection dose (%ID) of CpG was delivered to inguinal (IN) and axillary (AX) LNs, and IFA-emulsified CpG [IFA(CpG)] was substantially trapped at the injection sites with little delivered to draining LNs. In contrast, all MEB–CpG derivatives were efficiently delivered to LNs (IN + AX), with MH_2_C and MH_3_C being the most efficient (%ID: 0.62 MC, 1.08 MH_1_C, 1.49 MH_2_C, and 1.52 MH_3_C at 6 h post injection). MH_2_C peaked with 2.60 ± 0.30 %ID at 48 h post injection, with more than 1.45 %ID retained in LNs over 3 days. The area under the curve (AUC) of MC, MH_1_C, MH_2_C, and MH_3_C in these LNs were 3.0-, 5.5-, 6.5-, and 6.1-fold greater, respectively, than that of CpG within 3 days. The deep penetration of PET allowed to observe substantial amounts of MEB–CpG derivatives also in deep iliac (IL) LNs (Supplementary Fig. 13). PEG–CpG failed to enhance LN delivery, which suggests the essential and unique role of albumin for efficient LN delivery of albumin/AlbiVax nanocomplexes and rules out that the large size of albumin/AlbiCpG nanocomplexes was the sole factor contributing to efficient LN delivery. Significant amount of CpG and derivatives were still retained at injection sites (Supplementary Fig. 14), likely due to the slow lymphatic clearance and the interaction of phosphorothioate DNA with extracellular matrix. Consistently, high signal intensity and density of MEB–CpG derivatives in resected draining LNs were detected by γ counting. On day 3, IN LNs had 38.8 ± 6.3, 48.9 ± 6.2, 67.4 ± 9.3, and 59.5 ± 6.02 (mean ± standard error of mean (s.e.m.), same below; *n* = 4) percent injection dose per gram of tissue (%ID/g) of MC, MH_1_C, MH_2_C, and MH_3_C, respectively, in contrast to 8.3 ± 4.7, 4.0 ± 1.7, and 1.48 ± 0.55 (mean ± s.e.m.; *n* = 4) %ID/g of CpG, IFA(CpG), and PEG–CpG, respectively (Fig. 2d, Supplementary Fig. 15a–c). On day5, 2.5 ± 1.3, 37.3 ± 6.1, 38.6 ± 3.4,37.6 ± 1.8 (*n* = 4) %ID/g of CpG, MH_1_C, MH_2_C, and MH_3_C, respectively, were detected in IN LNs (Supplementary Fig. 15d, e). MEB–CpG derivatives were also observed in organs such as liver, kidney, and small intestine, presumably because of the ubiquity of albumin as well as the metabolism and renal clearance of CpG derivatives. Taken together, MH_2_C, which showed strong binding affinity with mouse serum albumin (MSA) (*K*_d_ = 1.0 μM; *R* ^2^ = 0.90) (Supplementary Fig. 16), was selected as the optimized AlbiCpG for further studies (Supplementary Fig. 11g). The efficient delivery of AlbiCpG was also verified by the purplish MEB color and strong MEB fluorescence of draining LNs (Supplementary Fig. 17). Overall, the efficient LN delivery of albumin/AlbiCpG nanocomplexes was attributed to a combination of relatively large sizes (*d*: ~13.0 nm; Fig. 2e and Supplementary Fig. 18), prolonged LN retention and efficient uptake by APCs (discussed below). Worth noting, AlbiCpG elicited less serum IL-6 and IL-12p40 than free CpG early after injection (Fig. 2f and Supplementary Fig. 19a) and milder splenomegaly (Fig. 2g and Supplementary Fig. 19b), suggesting the amelioration of acute systemic toxicity of CpG by reducing systemic dissemination^45^.

### Super-resolution analysis of nanovaccines in LNs and APCs

**Fig. 3:**
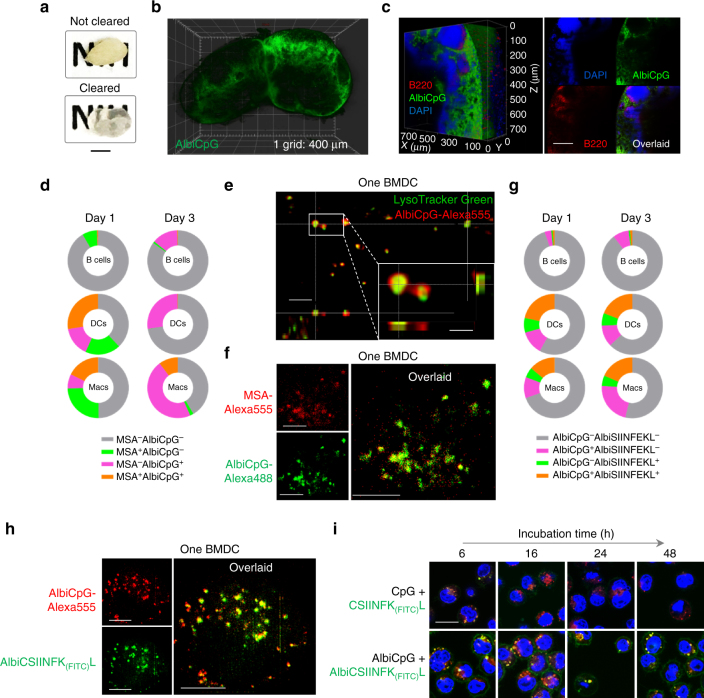
Super-resolution observation of intranodal and intracellular co-delivery of albumin/AlbiVax nanocomplexes. **a** Photographs of non-cleared and PACT-cleared mouse LNs. Scale bar: 2 cm. **b**,** c** Light sheet fluorescence microscopy images showing the 3D intranodal distribution of albumin/AlbiCpG–Alexa488 nanocomplexes in a whole LN (**b**) and a close-up of the subcapsular sinus areas (**c** left: 3D distribution; right: cross sections) 1 day after injection. Substantial albumin/AlbiCpG nanocomplexes were located within or near the subcapsular sinus and around B cell follicles (Supplementary Videos 2 and 3). Scale bar in **b**: 400 µm; Scale bar in **c**: 200 µm. **d** Fractions of IN LN B220^+^ B cells, CD11c^+^ DCs, and F4/80^+^ macrophages that took up AlbiCpG-Alexa555 and/or MSA-FITC, on day 1 and day 3 post s.c. injection of premixed AlbiCpG + MSA into C57BL/6 mice (*n = *4). **e** Deconvoluted confocal microscopy images showing AlbiCpG (200 nM) in the endolysosomes of one single BMDC after 2-h incubation. Inset: 2 endolysosomes showing AlbiCpG primarily on the endolysosome membrane. (Red: AlbiCpG; green: LysoTracker Green.) Scale bar: 2 µm; inset: 500 nm. **f** Instant SIM super-resolution images showing co-localization of MSA-Alexa555 (3 mg mL^−1^) and AlbiCpG-Alexa488 (200 nM) in one BMDC after 2-h incubation. Scale bars: 4 µm. **g** Fractions of IN LN B220^+^ B cells, CD11c^+^ DCs, and F4/80^+^ macrophages that took up AlbiCpG-Alexa555 and AlbiCSIINFEK_(FITC)_L on day 1 and day 3 post s.c. injection of AlbiCpG + AlbiSIINFEKL into C57BL/6 mice (*n = *4). **h** Instant SIM images showing intracellular co-delivery of AlbiCpG-Alexa555 (200 nM) + AlbiCSIINFEK_(FITC)_L (200 nM) in one BMDC after 2-h incubation. AlbiCSIINFEK_(FITC)_L was located together with AlbiCpG as well as separately in the cytosol. Scale bars: 2 µm. **i** Confocal microscopy images showing efficient antigen presentation of AlbiCSIINFEK_(FITC)_L (500 nM) + AlbiCpG (500 nM), compared with CSIINFEK_(FITC)_L (500 nM) + CpG (500 nM) in BMDCs. For incubation time longer than 6 h, fresh medium was substituted after 8-h incubation. (Blue: Hoechst 33342; green: FITC; red: LysoTracker Red.) Scale bars: 10 µm

The intranodal distribution of albumin/AlbiCpG nanocomplexes was further mapped by light sheet fluorescence microscopy (LSFM), which allows for higher resolution, 3D imaging of whole tissues with low photobleaching. Specifically, 1 day post s.c. injection of AlbiCpG-Alexa488 (higher photostability of Alexa488 than MEB) in C57BL/6 mice, draining IN LNs were resected and “cleared” to be transparent using passive CLARITY technique (PACT)^46^ (Fig. 3a). LSFM of the cleared LNs mapped the 3D distribution of intranodal AlbiCpG, which was especially abundant within or near the subcapsular sinus areas and around B cell follicles, as verified in LN slices (Fig. 3b, c, Supplementary Fig. 20, and Supplementary Movies 2 and 3).

We further analyzed the intracellular delivery of albumin/AlbiCpG nanocomplexes in APCs, a pivotal process for potent immunomodulation. Premixed MSA-FITC and AlbiCpG-Alexa555 were injected s.c. at the tail base of C57BL/6 mice. B220^+^ B cells, CD11c^+^ DCs, and F4/80^+^ macrophages were analyzed by flow cytometry on day 1 and day 3 post injection (Fig. 3d and Supplementary Fig. 21a, b). On day 1, 43% DCs and 27% macrophages were AlbiCpG^+^, and 28% DCs and 18% macrophages were AlbiCpG^+^MSA^+^, suggesting efficient uptake of albumin/AlbiCpG nanocomplexes. By contrast, only <1% B cells were AlbiCpG^+^, despite 7% MSA^+^ B cells. On day 3, 27% DCs, 55% macrophages, and 14% B cells were AlbiCpG^+^. MSA-FITC was only marginally detected, likely due to exhaustion. Efficient uptake of AlbiCpG was recapitulated in RAW264.7 macrophages and bone-marrow-derived dendritic cells (BMDCs) by γ counting of ^64^Cu-labeled AlbiCpG, confocal microscopy, and flow cytometry (Supplementary Fig. 21c–e). Deconvoluted confocal microscopy revealed ubiquitous AlbiCpG-Alexa555 in BMDC endolysosomes, and intriguingly, primarily on the endolysosome membranes wherein TLR9 resides^47^ (Fig. 3e, Supplementary Fig. 21f, and Supplementary Movie 4).

To study the intracellular vaccine delivery in APCs, we exploited instant SIM, a super-resolution imaging system that features ultrafast acquisition (up to 100 Hz) of 3D super-resolution images^41^. Dye-labeled MSA was rapidly endocytosed into BMDC endolysosome (Supplementary Fig. 21g), where MSA was co-localized with AlbiCpG when premixed MSA-Alexa555/AlbiCpG-Alexa488 was added to BMDCs (Fig. 3f), recapitulating intracellular co-delivery of albumin/AlbiCpG nanocomplexes by endocytosis. Albumin/AlbiCpG nanocomplexes upregulated the expression of costimulatory factor CD80 on DCs in draining LNs of C57BL/6 mice, despite accompanying lymphadenopathy (Supplementary Fig. 22a, b). In RAW264.7 macrophages and BMDCs, albumin/AlbiCpG nanocomplexes upregulated the expression of CD80 and CD86, and potently stimulated the production of TNFα, IL-6, and IL-12p40 (Supplementary Fig. 22c, d).

We then studied co-delivery of CpG and Ag by albumin/AlbiVax. We modified SIINFEKL, an epitope of ovalbumin (OVA), with cysteine for MEB conjugation and with FITC on lysine, which retained epitope binding ability to H-2K^b^ MHC class I^48^. The resulting MEB-CSIINFEK_(FITC)_L (denoted as AlbiCSIINFEK_(FITC)_L) was mixed with AlbiCpG-Alexa555 and s.c. co-injected at the tail base of C57BL/6 mice. After 1 and 3 days, IN LN DCs and macrophages, but not B cells, showed high uptake per cell (median fluorescence intensity, MFI) of both AlbiCpG and AlbiCSIINFEKL (Supplementary Fig. 23), with 15–20% AlbiCpG^+^AlbiCSIINFEKL^+^ DCs and macrophages (Fig. 3g), demonstrating efficient intracellular co-delivery of CpG and peptide Ag to LN APCs. Super-resolution imaging using instant SIM revealed that AlbiCSIINFEK_(FITC)_L and AlbiCpG-Alexa555 were co-localized in BMDCs within 2 h, recapitulating efficient intracellular co-delivery of CpG and Ag via albumin/AlbiVax (Fig. 3h). Moreover, some AlbiCSIINFEK_(FITC)_L was also located in the cytosol. Relative to CpG + CSIINFEK_(FITC)_L, AlbiCpG + AlbiCSIINFEK_(FITC)_L resulted in efficient and sustained antigen presentation in BMDCs (Fig. 3i and Supplementary Fig. 24a). The intracellular co-delivery was likely mediated by binding of AlbiCpG and AlbiCSIINFEKL with separate albumin molecules, because Förster resonance energy transfer between AlbiCpG-Alexa555 and AlbiCSIINFEK_(FITC)_L was undetectable in the presence of albumin (Supplementary Fig. 24b). We hypothesize that albumin/AlbiVax would dissociate in the acidic endolysosome, as evidenced by 20-fold weaker binding affinity of AlbiCpG with MSA at pH 5 than at pH 7.4 (Supplementary Fig. 24c). This dissociation will likely liberate AlbiVax, thereby preventing AlbiVax from trafficking out of APCs together with recycling albumin, and enhancing intracellular immunostimulation of AlbiCpG and cytosolic delivery of Ag.

### AlbiVax elicited potent and durable T cell responses

**Fig. 4:**
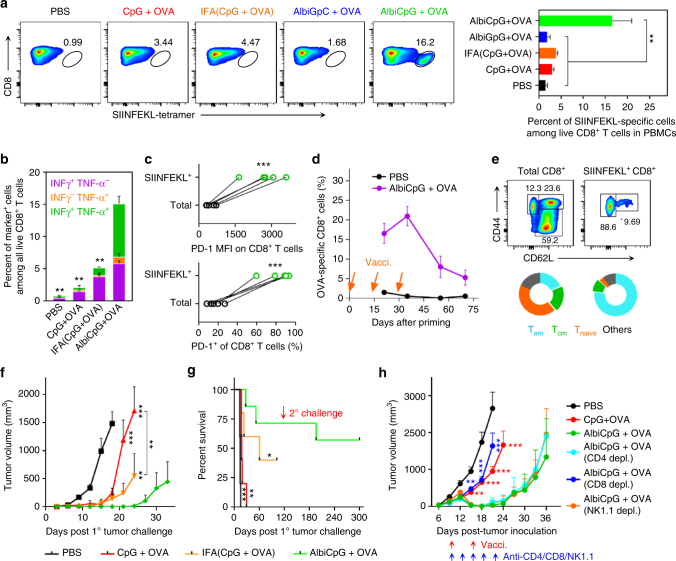
Albumin/AlbiVax nanocomplexes induced potent and durable antitumor T cell responses. **a**–**g** C57BL/6 mice (*n* = 5–7) were s.c. vaccinated with AlbiVax (2 nmol AlbiCpG equivalents + 10 µg OVA) at the tail base on day 0, day 14, and day 28, followed by immune analysis on day 21, day 35, and day 70, and 1° tumor challenge on day 71. **a** Representative flow cytometry plots (left) and frequency (right) of SIINFEKL^+^CD8^+^ T cells in peripheral blood on day 21 stained using phycoerythrin (PE)-labeled H-2K^b^-SIINFEKL tetramer. **b** Percentage of cytokine-producing CD8^+^ T cells in peripheral blood, measured by intracellular staining of IFN-γ and TNF-α on day 21. **c** Higher level (MFI) and frequency of PD-1 expression on SIINFEKL^+^CD8 T cells than that on total CD8^+^ T cells in peripheral blood on day 21. (Two-tailed paired *t* test.) **d** Frequencies of SIINFEKL^+^CD8^+^ CTLs in peripheral blood over 70 days post priming. **e** Representative flow cytometry results (upper) and percentage (lower) of effector memory T cells (Tem, CD62L^−^CD44^+^), central memory T cells (Tcm, CD62L^high^CD44^+^), and naive T cells (CD62L^+^CD44^−^) in peripheral blood on day 70, showing AlbiVax-induced T cell memory. **f**,** g** Tumor growth curve (**f**) and mouse survival (**g**) after s.c. challenging vaccinated mice with EG7.OVA cells. 1° challenge: 3 × 10^5^ cells on the right shoulder on day 71 post priming vaccination; 2° challenge: 1 × 10^6^ cells on the right flank on day 211. **h** AlbiCpG + OVA regressed established EG7.OVA tumor. C57BL/6 mice (*n* = 6–8) were s.c. inoculated with 3 × 10^5^ EG7.OVA cells on day 0, and treated with AlbiCpG + OVA (2 nmol CpG equivalents, 20 µg OVA) on day 6, day 12, and day 18. Lymphocyte depletion by anti-CD8, but not anti-CD4 or anti-NK1.1 (200 μg, on day 6, day 9, day 12, day 15, and day 18) abrogated the therapeutic efficacy of AlbiVax. ***p < 0.001, **p < 0.01, *p < 0.05, ns: not significant (*p* > 0.05), by one-way ANOVA with Bonferroni post test, unless denoted otherwise. Data show mean ± s.e.m. of two–three independent experiments. Asterisks in **a**, **b**, **f**–**h** indicate statistically significant differences between AlbiCpG and other groups

Albumin/AlbiCpG nanocomplexes were then studied for T cell responses with OVA. Compared with CpG + OVA, AlbiCpG + OVA (i.e., AlbiVax)-pulsed BMDCs markedly enhanced the proliferation of OT-1 CD8^+^ T cells (Supplementary Fig. 25a), suggesting that albumin/AlbiCpG promoted antigen cross presentation. We then immunized C57BL/6 mice with 2 nmol AlbiCpG and 10 μg OVA on day 0 and day 14, and stained CD8^+^ T cells in peripheral blood using a H-2K^b^-SIINFEKL tetramer on day 21 (Fig. 4a and Supplementary Fig. 25b). The frequencies of tetramer^+^CD8^+^ T cells were 2.9 ± 0.2% (*n* = 5) induced by CpG + OVA, 3.8 ± 0.2% (*n* = 5) by CpG + OVA emulsified in benchmark IFA, and by contrast, 16.5 ± 2.56% (*n* = 7) by AlbiCpG + OVA (*p < *0.01). No significant T cell responses were induced by control AlbiGpC + OVA, again indicating low immunogenicity of the MEB moiety. AlbiCpG + OVA increased the repertoires of polyfunctional IFNγ^+^, TNFα^+^, and IFNγ^+^TNFα^+^ peripheral CD8^+^ T cells, thus potentiating the cytotoxicity of CTLs (Fig. 4b and Supplementary Fig. 25c). AlbiCpG + OVA enhanced the serum titers of Ag-specific IgG2a, which benefits cancer therapy (Supplementary Fig. 25d). Upon immunization with AlbiCpG + OVA, 16.7 ± 3.2% (*n* = 5) of total peripheral CD8^+^ T cells expressed immune checkpoint PD-1 (Supplementary Fig. 25e, f). Among SIINKFEKL^+^CD8^+^ T cells, this frequency increased to 86.1 ± 6.1% (*n* = 7), and the PD-1 MFI was fivefold higher than that on total CD8^+^ T cells (Fig. 4c). The upregulated PD-1 expression upon AlbiVax elicited T cell activation suggests chronic antigen stimulation that eventually caused T cell exhaustion. Compared with total CD8^+^ T cells, these differential phenotypic characteristics of Ag-specific CD8^+^ T cells indicate that Ag-specific CD8^+^ T cells exhibit a more exhausted state upon vaccination, in line with clinical observations^4,49^. Given low spontaneous antitumor T cell responses in cancer patients and low to medium response rates of immune checkpoint blockade, the above observations provide the rationale to simultaneously elicit T cell responses and enhance the response rates to checkpoint inhibitors, such as anti-PD-1, for optimal therapy. A second boosting vaccination on day 28 further expanded the frequency of Ag-specific CD8^+^ CTLs to 21.0 ± 2.5% (*n* = 7) on day 35. This frequency remained >5% by day 70 (Fig. 4d), indicating immune memory, which was further demonstrated by a large repertoire of SIINKFEKL^+^ central memory T cells (CD8^+^CD44^+^CD62L^high^) on day 70 (Fig. 4e and Supplementary Fig. 26). On day 71, immunized mice were s.c. challenged with 3 × 10^5^ EG7.OVA cells. Marginal survival benefits were observed in mice vaccinated with CpG + OVA, despite early delay of tumor progression. IFA(CpG + OVA) moderately protected against challenge. By contrast, AlbiCpG + OVA significantly protected mice from challenge, with 71% (5/7) mice remaining tumor-free for more than 3 months (Fig. 4f, g). The surviving mice were re-challenged s.c. with 3 × 10^5^ EG7.OVA cells 120 days after 1° challenge, and 4/5 mice survived the 2° challenge for more than 6 months, again demonstrating durable T cell responses elicited by albumin/AlbiVax nanocomplexes. Of note, the binding of AlbiCpG with OVA (*K*_d_ = 2.3 μM; *R* ^2^ = 0.96. Supplementary Fig. 25g) likely contributed to co-delivery of AlbiCpG and OVA to LNs and T cell responses.

We then investigated AlbiCpG + OVA for immunotherapy of established tumors. C57BL/6 mice were s.c. inoculated with 3 × 10^5^ EG7.OVA, primed on day 6 post inoculation (~35 mm^3^ tumors), and boosted on day 12 and day 18 (2 nmol CpG, 20 µg OVA). While CpG + OVA moderately retarded tumor progression, AlbiCpG + OVA markedly regressed tumors in all mice upon boosting (Fig. 4h). The eventual tumor recurrence likely resulted from immunoregulatory checkpoints, such as PD-1 on CD8 CTLs and PD-L1 on EG7.OVA cells (Supplementary Fig. 27a), and combination of AlbiCpG + OVA with anti-PD-1 would likely reduce tumor recurrence. While depletion of CD4^+^ T cells or natural killer 1.1 (NK1.1) cells marginally affected the therapeutic outcome of AlbiCpG + OVA, depletion of CD8^+^ T cells nearly completely neutralized its efficacy, suggesting the central role of CD8^+^ CTLs in AlbiVax-based immunotherapy (Fig. 4h). To study the Ag specificity of immunotherapy, C57BL/6 mice were s.c. inoculated with 3 × 10^5^ EL4 cells (OVA^−^) on the left shoulder and 3 × 10^5^ EG7.OVA on the right shoulder. Treatment with AlbiCpG + OVA again regressed 4/6 EG7.OVA tumors, whereas EL4 tumor progression was barely affected (Supplementary Fig. 27b, c), thus confirming the Ag specificity of AlbiVax-mediated immunotherapy. Again, AlbiCpG + OVA elevated frequency of peripheral SIINFEKL^+^CD8^+^ CTLs (Supplementary Fig. 27d). Moreover, no morbidity or toxicity was observed in AlbiVax-treated mice (Supplementary Fig. 27e).

### Albumin/AlbiVax for combination melanoma immunotherapy

**Fig. 5:**
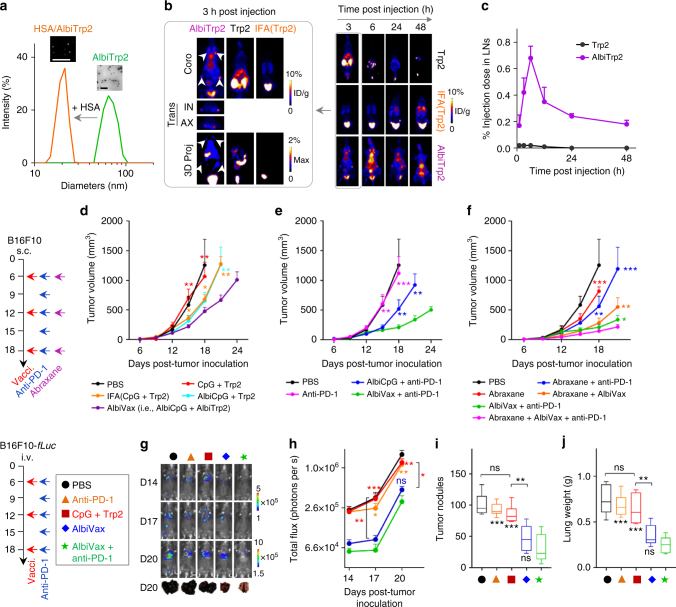
Albumin/AlbiVax nanocomplexes for melanoma combination immunotherapy. **a** Transformation of AlbiTrp2 nanoparticles into albumin/AlbiTrp2 nanocomplexes in the presence of HSA (molar ratio of HSA: albumin = 1:1). Insets: an AFM image (left) and a TEM image showing the corresponding nanoparticles. Scale bars: 200 nm. **b** Representative coronal, transverse, and 3D projection of PET images at 3 h post injection (left), and representative coronal PET images at 3, 6, 24, and 48 h post s.c. injection of AlbiTrp2, free Trp2, and IFA(Trp2) at the tail base of FVB mice. (Dose: 4.4–5.5 Mbq.) **c** Quantification of albumin/AlbiTrp2 nanocomplexes and Trp2 in draining LNs (IN + AX). White arrows mark LNs. **d**–**f** B16F10 tumor growth after treatment with AlbiVax (**d**), double combination of albumin/AlbiVax nanocomplexes and anti-PD−1 (**e**), and triple combination of albumin/AlbiVax nanocomplexes, anti-PD-1, and Abraxane (**f**). C57BL/6 mice were s.c. inoculated with 3 × 10^5^ B16F10 cells, treated with AlbiVax (2 nmol CpG equivalents + 20 µg AlbiTrp2) (day 6, day 12, and day 18), anti-PD-1 every 3 days from day 6 for five times (200 µg), and Abraxane on day 6, day 12, and day 18 (20 mg kg^−1^). **g**–**j** C57BL/6 mice were i.v. injected with 1 × 10^5^ B16F10-*fLuc* cells, treated with AlbiVax (2 nmol CpG equivalents + 20 µg AlbiTrp2) on day 6, day 12, and day 18 and anti-PD-1 (200 µg) every 3 days from day 6 for six times. **g** Representative bioluminescence images on day 14, day 17, and day 20, and photographs of lungs on day 20. **i** Quantified bioluminescence intensities of lungs. **i**,** j** Numbers of tumor nodules (**i**) and lung weights (**j**) on day 20. Data show mean ± s.e.m. of two–three independent experiments. ***p < 0.001, **p < 0.01, *p < 0.05, ns not significant (*p* > 0.05) by one-way ANOVA with Bonferroni post-test

We then studied peptide-Ag-based AlbiVax for immunotherapy of melanoma, an aggressive skin tumor whose clinical outcome can be improved by potentiating antitumor immunity. A melanoma-associated subunit Ag, tyrosinase-related protein 2 (Trp2), was used in this study. Trp2 modified with an N-terminal cysteine was conjugated with MEB. Hydrophilic MEB increased the water solubility of MEB–Trp2 conjugate relative to Trp2, resulting in self-assembled amphiphilic MEB–Trp2 nanoparticles in aqueous solution (Fig. 5a). MEB–Trp2 was tightly bound to albumin (*K*_d_ = 0.79 μM. *R* ^2^ = 0.88), which drove the transformation from MEB–Trp2 (AlbiTrp2) amphiphilic nanoparticles to albumin/AlbiTrp2 nanocomplexes and recovered MEB fluorescence (Fig. 5a and Supplementary Fig. 28). Trp2 delivery was quantitatively imaged by PET in FVB mice s.c. injected with ^64^Cu-labeled AlbiTrp2 at the tail base. While Trp2 was rapidly disseminated systemically, leaving no detectable Trp2 in draining LNs, albumin/AlbiTrp2 nanocomplexes were efficiently delivered to LNs with 91-fold larger AUC than Trp2 over 3 days (Fig. 5b, c). By contrast, IFA-emulsified Trp2 dramatically retained Trp2 at the injection sites, which might not only sequestrate, but also disarm and delete Ag-specific CD8^+^ CTLs.

Syngeneic C57BL/6 mice vaccinated with AlbiVax (i.e., AlbiCpG + AlbiTrp2) significantly resisted a s.c. challenge of 3 × 10^5^ B16F10 cells, in contrast to marginal protection by CpG + Trp2 or IFA(CpG + Trp2) (Supplementary Fig. 29a). In B16F10 tumor-bearing C57BL/6 mice, PET verified that both AlbiCpG and AlbiTrp2 were efficiently delivered to LNs, except for tumor draining LNs due to damaged lymphatic drainage in tumors (Supplementary Fig. 29b–e). For immunotherapy, C57BL/6 mice s.c. inoculated with 3 × 10^5^ B16F10 cells were treated with AlbiVax (2 nmol CpG equivalent, 20 µg AlbiTrp2) on day 6, day 12, and day 18 post inoculation. While none of CpG + Trp2, AlbiCpG + Trp2, or IFA(CpG + Trp2) significantly inhibited tumor progression, albumin/AlbiVax nanocomplexes significantly prohibited tumor growth (Fig. 5d). Given the high expression of PD-L1 on B16F10 tumor cells (Supplementary Fig. 29f), a combination of anti-PD-1 and albumin/AlbiVax nanocomplexes enhanced the therapeutic efficacy (Fig. 5e). A triple combination of anti-PD-1, albumin/AlbiVax nanocomplexes, and chemotherapeutic Abraxane that enhances tumor immunity^50,51^, further inhibited the tumor progression (Fig. 5f). No tumors were eradicated, but we envision that enhanced therapeutic efficacy can be achieved using multi-epitope albumin/AlbiVax to induce a broader spectrum of antitumor T cell responses, or by combining with more synergistic therapeutics^16,32^. Taking advantage of the systemic T cell responses induced by albumin/AlbiVax, we then investigated immunotherapy of metastatic melanoma, a primary death cause of melanoma patients. A lung metastatic B16F10 model was established by intravenous (i.v.) injection of 1 × 10^5^ B16F10-*fLuc* cells into C57BL/6 mice. When lung metastases were established, mice were again treated with AlbiVax on day 6, day 12, and day 18, and anti-PD-1 on day 6, day 9, day 12, day 15, and day 18 post-cell injection. Lung metastatic B16F10-*fLuc* burdens were monitored by bioluminescence imaging (Fig. 5g, h). Neither CpG + Trp2 nor anti-PD-1 alone significantly inhibited tumor progression, whereas albumin/AlbiVax inhibited the deposition and progression of lung metastases (Fig. 5i, j). Again, combining albumin/AlbiVax nanocomplexes and anti-PD-1 further potentiated the therapeutic efficacy.

### Neoantigen-based albumin/AlbiVax for tumor immunotherapy

**Fig. 6:**
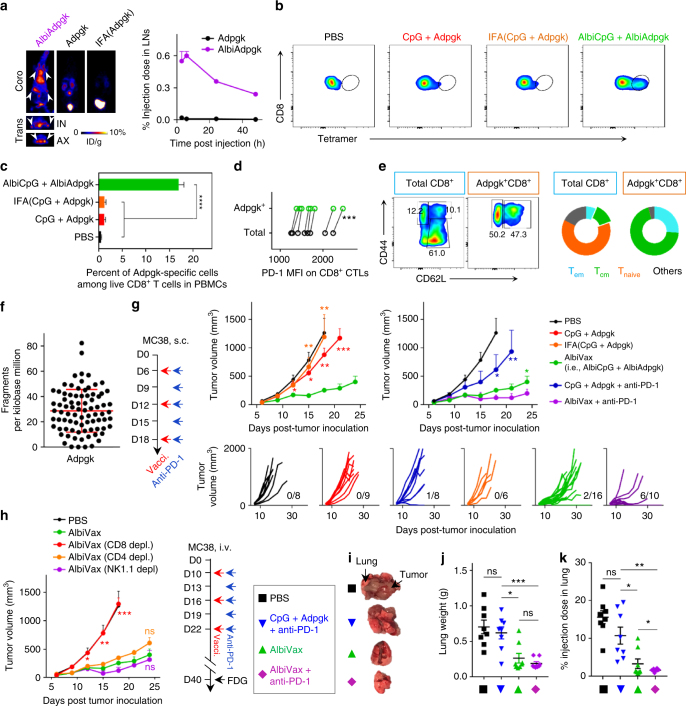
Neoantigen-based albumin/AlbiVax nanocomplexes for personalized cancer immunotherapy. **a** Upper: representative coronal and transverse PET images at 6 h post injection, and lower: quantification of albumin/AlbiAdpgk nanocomplexes and Adpgk in IN and AX LNs of FVB mice (*n* = 4). White arrows mark LNs. IFA(Adpgk) was undetectable in LNs. (Dose: 4.4–5.5 Mbq) **b**–**e** C57BL/6 mice (*n* = 5) were vaccinated with AlbiVax (2 nmol AlbiCpG + 20 µg AlbiAdpgk) on day 0 and day 14, followed by flow cytometric analysis of H-2D^b^-ASMTNMELM tetramer^+^CD8^+^ T cells (**b**, **c**) and PD-1 expression on peripheral CD8^+^ T cells (**d**) on day 21, and CD8^+^ T cell central memory (CD62L^high^CD44^+^) on day 50 (**e**). **f** Exome-sequencing results verifying *Adpgk* variant in MC38 cells. **g**, **h** MC38 tumor growth after treatment with AlbiVax alone or in combination with anti-PD-1. C57BL/6 mice were s.c. inoculated with 3 × 10^5^ MC38 cells, treated with AlbiVax (2 nmol AlbiCpG + 20 µg AlbiAdpgk) on day 6, day 12, and day 18), and with anti-PD-1 (200 µg) every 3 days from day 6 for six times. **h** Depletion of CD8^+^ T cells, but not CD4^+^ T cells or NK cells abrogated the therapeutic efficacy of AlbiVax (*n = *5). **i**–**k** Immunotherapy of lung metastatic MC38 tumor with AlbiVax alone or in combination with anti-PD-1. C57BL/6 mice were i.v. inoculated with 1 × 10^5^ MC38 cells, treated with AlbiVax (2 nmol AlbiCpG + 20 µg AlbiAdpgk) on day 10, day 16, and day 22), and with anti-PD-1 (200 µg) every 3 days from day 10 for six times. On day 40, mice were injected with FDG tracer (100 µCi). Mice were killed and lungs and tumors were collected (**i**), weighted (**j**), and radioactivity measured by γ-counting (**k**). Data show mean ± s.e.m. of two–three independent experiments. ***p < 0.001, **p < 0.01, *p < 0.05, ns: not significant (*p* > 0.05) by one-way ANOVA with Bonferroni post-test

We next explored albumin/AlbiVax nanocomplexes for neoantigen-based personalized tumor immunotherapy. We used Adpgk, a neoantigen [ASMTN(R → M)ELM] in MC38 tumors^6^. The conjugation of hydrophilic MEB improved the water solubility of MEB-Adpgk, and entailed MEB-Adpgk to strongly bind to albumin (*K*_d_ = 1.1 μM. *R* ^2^ = 0.96) and form amphiphilic nanoparticles in aqueous solution (Supplementary Fig. 30a, b). Using ^64^Cu-labeled NMEB-Adpgk (denoted MEB-Adpgk as AlbiAdpgk), PET demonstrated efficient LN delivery of albumin/AlbiAdpgk, and the AUC in IN and AX LNs was 43-fold higher than that of Adpgk within 2 days (Fig. 6a and Supplementary Fig. 30c, d). IFA(Adpgk) was again trapped in the injection sites, and despite the particulate formulation of AlbiAdpgk, significantly less AlbiAdpgk was trapped at the injection sites than IFA(Adpgk) (Supplementary Fig. 30e), which is particularly significant for subunit Ag-based vaccination to prevent the dysfunction of functional CD8^+^ CTLs. Immunization of C57BL/6 mice with AlbiVax (AlbiCpG + AlbiAdpgk) on day 0 and day 14 elicited 14.1- and 13.6-fold greater frequency of peripheral Adpgk^+^CD8^+^ CTLs than CpG + Adpgk and IFA(CpG + Adpgk), respectively, as stained using a H-2D^b^-ASMTNMELM tetramer (Fig. 6b, c). Albumin/AlbiVax upregulated PD-1 expression on peripheral CD8^+^ CTLs, especially Adpgk^+^CD8^+^ CTLs (Fig. 6d), suggesting that T cell responses were accompanied with T cell exhaustion that justified PD-1 blockade for optimal therapy. Further, albumin/AlbiVax induced central T cell memory for over 50 days (Fig. 6e). Immunizing C57BL/6 mice with AlbiVax on day 0 and day 14 markedly retarded MC38 tumor progression after s.c. challenge with 3 × 10^5^ MC38 cells on day 30 (Supplementary Fig. 30f).

To study albumin/AlbiVax nanocomplexes for personalized immunotherapy of MC38 tumor, the *Adpgk* variant was verified in MC38 cells by exome sequencing (Fig. 6f). A total of 3 × 10^5^ MC38 cells were s.c. inoculated in C57BL/6 mice, and treatment was initiated on day 6 when tumors were established (~30 mm^3^). In contrast to the moderate inhibition of tumor progression by IFA(CpG + Adpgk), albumin/AlbiVax inhibited tumor progression significantly more effectively (Fig. 6g). Most tumors failed to regress, in part due to immune checkpoints PD-1 on CD8^+^ CTLs (Fig. 6c) and PD-L1 on MC38 cells (Supplementary Fig. 30g). Thus, by combining anti-PD-1 with AlbiVax, CD8^+^ CTLs reinvigoration markedly increased the response rate and led to complete regression of 6/10 tumors for 4 months (Fig. 6g and Supplementary Fig. 30h). These tumor-free mice resisted a rechallenge with MC38 cells, again suggesting long-term immune memory. Lymphocyte depletion demonstrated that CD8^+^ T cells, but not CD4 T cells or NK1.1 cells, were vital to AlbiVax-mediated immunotherapy (Fig. 6h). Lung is a primary destination of colon cancer metastases. Lung metastatic-like tumors were established by i.v. injection of 1 × 10^5^ MC38 cells into C57BL/6 mice. From day 10 post-tumor inoculation, mice were treated with AlbiVax every 6 days for three times and anti-PD-1 every 3 days for six times. On day 40, the metabolic activity of lung metastatic-like tumors was studied using ^18^F-fluorodeoxyglucose (FDG), as shown by PET-CT of PBS-treated mice (Supplemental Fig. 30i). Organs of interest were resected, weighted, and radioactivity was measured by γ counting. Tumor burdens (Fig. 6i, j) and FDG radioactivity (Fig. 6k and Supplemental Fig. 30j) of lung metastatic-like tumors suggest that albumin/AlbiVax significantly reduced tumor progression, and combination with anti-PD-1 further potentiated the therapeutic efficacy (Fig. 6k).

## Discussion

Safe and robust T cell vaccines would hold great promise for combination cancer immunotherapy. Locally administered vaccines must provide efficient delivery to LNs for effective antitumor immunomodulation. Nanovaccines are a promising approach for efficient vaccine delivery to LNs, and quite a few have been developed preclinically, yet the clinical translation of these nanoformulas has been hindered by limitations in their safety, suitability for reproducible large-scale manufacturing, and in vivo integrity. We chose albumin, one of the most successful carriers of nanomedicines in the clinic, to deliver anti-cancer molecular vaccines in this study. We exploited the ability of clinically safe EB to bind with endogenous albumin and to concentrate within LNs, and we engineered AlbiVax by conjugating EB derivatives with molecular vaccines to efficiently co-deliver adjuvant and peptide Ags into LNs for combination cancer immunotherapy. In contrast to synthetic nanomedicines, such as Abraxane, albumin/AlbiVax nanocomplexes were assembled from endogenous albumin and exogenous molecular vaccines in vivo. This approach is intrinsically attractive for multiple reasons. First, EB-based AlbiVax has shown a good safety profile. EB had been considerably practiced in the clinic, and currently no side effects of EB derivatives were observed in mice or in two clinical trials of systemically or locally injected EB derivatives in hundreds of patients and healthy volunteers^36,39^. Moreover, rhesus monkeys survived 25 mg kg^−1^ systemically injected EB^40^. Though the safety of albumin/AlbiVax nanocomplexes remains to be systematically determined, a low dose is sufficient for potent immunization (0.4 mg kg^−1^ MEB equivalent, <0.2% of dose used in the above monkeys). Moreover, local administration and reduced systemic dissemination of AlbiVax further mitigate toxicity risk. Second, chemically defined AlbiVax are amenable to relatively easy large-scale manufacturing, formulation, and quality control, and they offer long shelf-life. Large-scale synthesis of AlbiVax via a biocompatible thiol-maleimide conjugation is expected to be generally applicable for molecular vaccines. Third, AlbiVax harnesses endogenous albumin to assemble nanocomplexes, thus avoiding the complications involved with safety, large-scale production and quality control of synthetic nanomaterials. Fourth, albumin/AlbiVax nanocomplexes have a comprehensive and sophisticated delivery mechanism: efficient delivery to LNs by lymphatic drainage, efficient intracellular delivery to APCs by endocytosis, and conditional intracellular vaccine release by albumin/AlbiVax dissociation in acidic endolysosomes. Efficient intracellular vaccine delivery is essential for Ag cross presentation and robust T cell responses. Internalized albumin/AlbiVax nanocomplexes in APCs is dissociated in the acidic endolysosome, probably due to loosened binding, thereby liberating AlbiVax for intracellular immunomodulation and preventing AlbiVax from efflux during albumin recycling. Fifth, EB-based AlbiVax is expected to be widely applicable for therapeutics ranging from peptides, nucleic acids, lipids, polysaccharides, to small molecules. AlbiVax was synthesized via conjugation of MEB and a thiol that can be easily modified for many chemical or biologic therapeutics; alternatively, we also developed EB derivatives with alkyne and amine that can be used for AlbiVax conjugation. Recombinant albumin–drug conjugates, by contrast, can only be applied to protein or peptide vaccines, which are inefficient at intracellular vaccines release that is however pivotal for potent immunomodulation; moreover, using endogenous albumin would obviate problems resulting from contamination during biologics production or immunogenic responses of exogenous albumin conjugates. Covalent conjugation of exogenous therapeutics to the cysteine-34 position of endogenous albumin, though prolong circulation, is limited again by inefficient intracellular vaccine release and nonspecific conjugation with other cysteine-accessible biomolecules including apolipoprotein B-100. Compared with albumin-binding peptides and hydrophobic lipids, hydrophilic EB derivatives may improve drug formulation especially for hydrophobic drugs, and avoid off-target interactions with surrounding biomolecules or cell membranes^52^. PEG can improve the aqueous solubility, but also adds up cost and increases the risk of allergy against PEG^53^, especially when an immunostimulatory adjuvant is co-administered.

We systematically and quantitatively studied vaccine delivery and revealed the spatiotemporal intranodal and intracellular behaviors of AlbiVax by multiscale imaging, including PET in small animals, light sheet fluorescence microscopy in whole LNs, and super-resolution fluorescence imaging in single APCs. Interestingly, super-resolution imaging allowed us to reveal that AlbiCpG was located primarily on APC endolysosome membrane likely due to binding to TLR9 on the membranes, and we observed substantial co-localization of AlbiCpG and AlbiAg in APCs as a result of albumin-mediated endocytosis. These observations may provide insight for the underlying mechanism of AlbiVax-mediated immunomodulation. Albumin/AlbiVax nanocomplexes co-delivered adjuvants and Ags to APCs and enabled sustained Ag presentation on APCs. In mice, albumin/AlbiVax nanocomplexes dramatically enhanced the potency and durability of T cell responses, and significantly inhibited tumor progression or eradicated tumors in multiple primary or metastatic tumors. There are over 1000 clinical trials of combination cancer immunotherapy involving vaccines currently under enthusiastic pursuit^54^. The principle that albumin/AlbiVax nanocomplexes are amenable for combination therapy with immune checkpoint inhibitors and chemotherapy is encouraging preclinical evidence supporting AlbiVax-based combination immunotherapy.

Albumin/AlbiVax nanocomplexes are readily applicable to neoantigen vaccination for personalized tumor immunotherapy. Although tumor immunotherapy benefits from high loads of tumor-specific mutations and high frequencies of neoantigen-specific CTLs^7,9,55–57^, natural neoantigen-specific CD8^+^ CTLs are often rare (e.g., 0.002–0.4% in melanoma, which already has the second highest tumor mutation loads^58,59^), likely due to low clonal neoantigen burden, inefficient antigen processing and cross presentation, and immunosuppression. Exogenous neoantigen vaccines delivered by albumin/AlbiVax nanocomplexes can potentiate neoantigen-specific immunity and potentiate tumor therapy. Further, combining checkpoint inhibitors with albumin/AlbiVax nanocomplexes can further unleash CTL activity and enhance therapeutic efficacy. While it currently takes a relatively long time to identify and manufacture neoantigen-based AlbiVax, patients can be treated with other regimens before AlbiVax.

In summary, by in vivo assembly with endogenous albumin, albumin/AlbiVax nanocomplexes represent a widely applicable T cell vaccine that, as quantitatively determined, is efficiently delivered into LNs, ameliorates side effects, induces potent and durable T cell responses, and as part of combination immunotherapy inhibits or eradicates established tumors.

## Methods

### DNA synthesis

DNA was synthesized at a 1 µmole scale of solid phase synthesis on an ABI 392 DNA synthesizer (Applied Biosystems) using materials purchased from Glen Research (Sterling, VA) or Chemgenes (Wilmington, MA). DNA was deprotected accordingly to the manufacturer’s instructions, and purified by reverse phase HPLC (Dionex Ultimate 3000, Thermo Fisher Scientific, Waltham, MA). Dimethoxytrityl (DMT) protecting group on DNA was removed using 0.5 M acetic acid. DNA was desalted and quantified on a Genesys 10 s UV-Vis spectrometer (Thermo Fisher Scientific, Waltham, MA). Fluorophores, thiol, or HEG were modified following the manufacturer’s instructions. All DNAs, including HEG linkers, had phosphorothioate backbone. DNA modified with Alexa488 or Alexa555 were from Integrated DNA Technology (Coralville, IA).

### AlbiVax synthesis

CpG1826 (TCCATGACGTTCCTGACGTT) and GpC control (TCCATGAGCTTCCTGAGCTT) were used. AlbiCpG or derivatives were synthesized using MEB and terminal thiol-modified CpG. For AlbiCpG screening, four CpG derivatives with 0, 1, 2, and 3 hexaethyloxy-glycol (HEG) units between MEB (confirmed by NMR analysis)^35^ and CpG were used. Thiol-modified DNA was pretreated with DTT (0.1 M) in PBS (37 °C, 1 h) to cleave the dithiol bond, followed by desalting using a NAP5 column in sodium ascorbate buffer (0.1%) to remove DTT and the thiol-appending small fragment cleaved from DNA. The resulting DNA (200 nmol) was mixed with MEB (1 mg) in 2 mL sodium ascorbate buffer (0.1%) in PBS and reacted at room temperature for 30 min. The resulting product was purified again using a NAP5 column to remove excess MEB, and quantified by UV absorbance at 260 nm (where MEB has negligible absorption compared with CpG) or by weighing lyophilized products in the case of synthesis at a relatively large scale.

MEB–antigen conjugates were synthesized using MEB and antigens (CSIINFEKL, Trp2, and Adpgk) that were modified with N-terminal cysteine. (Trp2: CSVYDFFVWL; Adpgk: CGIPVHLELASMTNMELMSSIVHQQVFPT.) In a typical reaction, MEB (10 mg) dissolved in water (1 mL) was added to peptide (10 mg) dissolved in DMF (2 mL) dropwise. The reaction mixture was agitated at room temperature for at least 1 day. MEB–antigen conjugates were purified using a C18 column on HPLC.

AlbiVax was verified using liquid chromatography–electrospray ionization–tandem mass spectrometry (LC–ESI−MS).

### AFM and TEM

The sizes and morphologies of nanomaterials were characterized using transmission electron microscopy (TEM) and atomic force microscopy (AFM). TEM samples of AlbiVax or a mixture of AlbiVax and HSA were prepared by depositing samples (10 µL) onto a carbon-coated copper grid. For AFM, samples (10 µL) were casted on freshly peeled mica substrate, followed by drying, rinsing, and dehumidifying. AFM was carried out in tapping mode in air on a PicoForce Multimode AFM (Bruker, CA) equipped with a Nanoscope® V controller, a type E scanner head, and a sharpened TESP-SS (Bruker, CA) AFM cantilever. AFM images were then analyzed by Nanoscope Software (version 7.3–8.15, Bruker, CA).

### Molecular docking and prediction of albumin/MEB binding

The HSA model was constructed using SwissPdb Viewer^60,61^ based on the X-ray complex structure of indoxyl sulfate with HSA (Protein Data Bank (PDB) ID code 2BXH)^33^, with the ligand and water molecules removed. The stereochemical restraints were obtained from the CHARMM molecular mechanics force field^62^. The model was then calculated via cycles of geometry optimization and molecular dynamics simulation using NAMD^63^, with energy-minimized HSA structure. The stereochemical quality was analyzed using PROCHECK^64^.

Automated molecular docking was conducted using AutoDock version 4.2.6^65^. The Lamarckian genetic algorithm (LGA) was applied to model the binding of MEB with HSA. For molecular docking, LGA describes the relationship between a ligand and a protein by the translation, orientation, and conformation of the ligand. The final solution provides a ligand conformation finely tuned to the protein^66^. The docking area (active site) of HSA was defined using the AutoDock module AutoGrid. The grid site was constrained to a 28.5 Å cubic space, including the entire binding site of HSA and providing sufficient space for translational and rotational walk of the ligand. The LGA was applied to search the conformational and orientational space of MEB while keeping the HSA structure rigid. Default parameters were used, except the maximum energy evaluations of 1.5 × 10^6^. With 10 independent runs, a maximum of 2.7 × 10^4^ genetic algorithm operations were generated on a single population of 50 individuals. Further, binding free energy and affinity constant were calculated, and the docked complex was selected based on matching interaction energies with geometric quality. Further energy minimization and geometric optimization were performed on the select complex until no more conflicts among the ligand and HSA.

A score function at binding free energy was derived and adopted in AutoDock^65^, based on the traditional molecular force field model of interaction energy. The restriction of internal rotors, global rotation, and translation were modeled depending on the calculated torsion angles of the ligand. The total binding free energy was empirically calibrated, using 30 structurally known protein–ligand complexes with experimentally determined binding constants. The score function is sufficient to rank the ligands on level of binding affinity and free energy (∆*G*_binding_ values), as well as the inhibition constant (*K*_i_). With this approach, we then calculated the binding free energy and inhibition constant.

### Photophysics of MEB–DNA

Photophysics of MEB–DNA was studied in terms of fluorescence spectrometry, intensity, and lifetime, in the absence or presence of albumin. All measurements were conducted on a fluorometer (Jobin Yvon, HORIBA) using excitation and emission parameters specific for MEB.

### Kinetic binding assay by BLI

The kinetic binding of AlbiVax with albumin was measured by BLI on an Octet Red96 system (fortéBio) using streptavidin-coated biosensors (fortéBio). Assays were carried out with agitation in solid black 96-well plates (Geiger Bio-One) in seven steps: 1, baseline (1x PBS), time: 60 s; 2, loading (Biotin-labeled albumin, 1 µg mL^−1^), time: 600 s; 3, baseline (1× PBS), time: 60 s; 4, quenching (1 µg mL^−1^ Biocytin (Thermo Scientific)), time: 180 s; 5, baseline (1× PBS), time: 60 s; 6, association, time: 600 s; 7, dissociation, time: 600 s. Nonspecific binding was performed by measuring the binding of albumin-loaded biosensor to buffer alone and blank biosensor to analytes. Data analysis and curve fitting were performed using Octet Analysis software 7.0. Binding data were fitted with a binding model of 1:1 ligand interaction using GraphPad Prism 7 (La Jolla, CA).

### Animal studies

All animal work was conducted in appliance to the NIH Guide for the Care and Use of Animals under protocols approved by the NIH Clinical Center Animal Care and Use Committee. Female C57BL/6j mice (6–8 weeks. The Jackson Laboratory) were used for fluorescence imaging of AlbiVax delivery, immunology analysis, and tumor therapy studies. Female FVB mice (6–8 weeks) were used in all PET studies of AlbiVax delivery in tumor-free mice as well as toxicity studies of AlbiCpG.

### PET imaging and biodistribution of AlbiVax

AlbiVax was radiolabeled with ^64^Cu for PET. To label ^64^Cu via chelation, a NOTA-functionalized MEB (NMEB) was synthesized and conjugated with vaccines^44^. Specifically, ^64^CuCl_2_ was converted to ^64^Cu-acetate by adding 0.5 mL of 0.4 M NH_4_OAc solution (pH 5.6) to 20 µL ^64^CuCl_2_. ^64^Cu-acetate solution (0.1 mL; 3–4 mCi) was added into a solution of 100 µg of CpG or NMEB-CpG or derivatives in water, or NMEB-Adpgk/NMEB-Trp2 in DMSO (10 mg mL^−1^). The reaction was shaken for 0.5 h at 37 °C. Thereafter, the radiochemical purity was determined using iTLC plates (Fisher Scientific), developed in 0.1 M citric acid (pH 5). Crude reaction of CpG or NMEB-CpG derivatives were purified on a Nap5 column (GE Healthcare) and the desired product was eluted in saline. NMEB-Adpgk and NMEB-Trp2 were purified on a C18 Sep-Pak (BOND-ELUT 100 mg, Varian); then NMEB peptides were eluted from the cartridge using 70% ethanol and 30% PBS.

Female FVB mice (6–8 weeks) were used in all PET imaging studies in naive mice. C57BL/6 mice (6–8 weeks) were used in PET imaging of tumor-bearing mice. Mice were anesthetized using isoflurane/O^2^ (2% v v^−1^) before injection. Anesthetized mice were injected s.c. at the base of tail with ^64^Cu-labeled vaccines (4.44−5.55 MBq/120–150 μCi each mouse) in PBS (100 μL). At indicated time points post injection, mice were scanned on an Inveon DPET scanner (Siemens Medical Solutions, Malvern, PA). PET images were reconstructed without correction for attenuation or scattering using a three-dimensional ordered subsets expectation maximization algorithm. ASI Pro VM^TM^ software was used for image analysis. Regions of interest (ROI) were drawn on LNs to calculate the %ID/g.

The above mice were killed at specified time points. Organs and blood were collected and wet weighed. The collected organs and blood, together with a series of standard solutions, were measured for ^64^Cu radioactivity on a gamma counter (Wallac Wizard 1480, PerkinElmer). The radioactivity of organs and blood was converted to calculate the percentages of the injected dose (%ID) in organs of interest and the percentages of the injected dose per gram of tissue (%ID/g).

### LN clearing and light sheet fluorescence imaging of LNs

Draining LNs of vaccinated C57Bl/6 mice were cleared using a modified PACT (passive CLARITY technique)^46^. Particularly, female C57BL/6 mice (6–8 weeks) were injected with AlbiCpG (5 nmol each mouse) s.c. at the base of the tail. After 24 h, transcardiac perfusion was conduct with 6 mL PBS and then 6 mL 4% paraformaldehyde (PFA). PFA-fixed LNs were then isolated and incubated at 4 °C overnight in hydrogel monomer solution in PBS containing 4% acrylamide and 0.25% photoinitiator 2,20-Azobis[2-(2-imidazolin-2-yl) propane] dihydrochloride (VA-044, Wako Chemicals USA). LNs infused with hydrogel monomer were degassed with nitrogen for 5 min and then incubated for 2 h at 37 °C to initiate tissue-hydrogel crosslinking. LNs were then washed with PBS to remove excess hydrogel. For tissue clearing, LN-hydrogel matrices were incubated in 8% SDS in 0.1 M PBS (pH 7.5) for 2 days at 37 °C with gentle shaking. LNs were cleared well and were then washed with PBS for four times in 1 day.

To image cleared LNs, LNs were mounted in refractive index matching solution (RIMS) with a refractive index (RI) of 1.46, for 1 day before imaging. RIMS was prepared by adding 40 g Histodenz (Sigma D2158) in 30 mL 0.02 M PB (pH 7.5) with 0.1% Tween-20 and 0.01% sodium azide. To mount LNs for imaging, LNs were embedded in 2% agarose in a 500-mL syringe. Once agarose was gelled in the syringe, the LN-gel matrices were push partially out of syringe and immersed in RIMS for 1 day to allow RIMS to penetrate the agarose gel and create a homogeneous imaging media.

Light sheet 3D imaging of LNs were performed on a Lightsheet Z.1 light sheet microscope (Carl Zeiss Microscopy, LLC).

### Cell lines and cell culture

EL4 and EG7.OVA cells were from Dr Joshua Farber’s laboratory at NIAID, NIH. EL4 cells were cultured in RPMI medium with 2 mM l-glutamine, 10% heat-inactivated FBS, and 1% penicillin and streptomycin; EG7.OVA cells were cultured in RPMI medium with 2 mM l-glutamine, 10% heat-inactivated FBS, and 0.4 mg mL^−1^ G418. MC38 cells were from Dr Robert Seder Laboratory at NIH Vaccine Research Center. RAW264.7 cells (from ATCC), B16F10 cells (from ATCC), and MC38 cells were cultured in DMEM medium with 10% heat-inactivated FBS and 1% penicillin and streptomycin. DC2.4 cells were from Dr Jonathan W. Yewdell Lab at NIAID, and cultured in RPMI medium with 2 mM l-glutamine, 10% heat-inactivated FBS, and 1% penicillin and streptomycin. Cells were grown in a humidified atmosphere (5% CO_2_, 37 °C). All cells were tested to be free of mycoplasma.

### Antibodies for flow cytometry and tissue staining

Antibodies were from BD Bioscience, eBioscience, and BioLegend. The following antibodies are listed in the order of antigen, fluorophore, clone, sources, catalog number, and dilution times for the final concentrations. CD11c, FITC, N418, Biolegend, 117306, 1600; CD11c, PE, HL3, BD, 553802, 800; F4/80, APC, BM8, eBioscience, 17–4801–82, 800; CD3, PerCP Cy5.5, 17A2, Biolegend, 100218, 800; B220, BV421, RA3-6B2, Biolegend, 103240, 800; B220, Al647, RA3-6B2, Biolegend, 103226, 1600; CD8a, APC-Cy7, 53-6.7, Biolegend, 100714, 800; CD8a, APC, 53-6.7, BD, 553035, 800; PD-1, BV421, 29F.1A12, Biolegend, 135221, 800; PD-L1, BV421, 10F.9G2, Biolegend, 124315, 800; IFN-gamma, PE, XMG1.2, BD, 554412, 100; IFN-gamma, FITC, XMG1.2, eBioscience, 11-7311-82, 100; TNF-alpha, FITC, MP6-XT22, BD, 560659, 100; TNF-alpha, APC, MP6-XT22, eBioscience, 560658, 100; CD62L, FITC, MEL-14, BD, 553150; CD44, PE-Cy5, IM7, BD, 561861. Antibodies were used according to the manufacturer’s instructions, including antibody dilution. Flow cytometry was conducted on a BD LSRFortessa X-50 flow cytometer at the Core Flow Cytometry Facility of Vaccine Research Center.

### In vitro cell uptake of AlbiCpG in APC

In vitro cell uptake was first studied using confocal laser scanning microscopy and flow cytometry. FITC-labeled AlbiCpG were incubated with RAW264.7 cells, DC2.4 cells, or BMDCs, and stained with Lysotracker Red DND-99 (Life Technologies, Carlsbad, CA) and 10 µg mL^−1^ Hoechst 33342 (Life Technologies, Carlsbad, CA) for 0.5 h in a cell culture incubator at 37 °C, prior to observation. Cells were then washed with Dulbecco’s PBS for three times before imaging on a Zeiss LSM 780 confocal microscope (Chesterfield, VA). Alternatively, flow cytometry was used to study the cell uptake using a BD Beckman Coulter flow cytometer (Brea, CA) or BD Accuri C6 flow cytometer (San Jose, CA). For flow cytometric analysis, RAW264.7 cells, or DC2.4 cells, or BMDC cells were seeded into 24-well plate, and 1 day later, cells were treated with AlbiCpG for a specified time, followed by flow cytometric analysis. Flow cytometry results were analyzed by using FlowJo V10 software.

### In vitro cell uptake of AlbiCpG into APCs by γ-counting

The cell uptake of AlbiCpG was also studied using ^64^Cu-labeled AlbiCpG. RAW264.7 cells or DC2.4 cells (2 × 10^5^ cells per well) were seeded onto a 12-well plate, and 1 day later, cells were treated with AlbiCpG (2 uCi per well) for a specified time, followed by washing with Dulbecco’s PBS, treating cells with NaOH solution, and collecting the resulting solution to measure the radioactivity on a γ counter.

### Super-resolution imaging of intracellular AlbiCpG in APCs

Cultured cells were treated with AlbiCpG for 2 h and washed with PBS. Endolysosome was stained with LysoTracker Green (Invitrogen, Carlsbad, CA) according to the manufacturer’s instruction. Cells were immersed in HEPES buffer (pH 7.4) during imaging. Super-resolution imaging was conducted on an instant linear structured illumination microscope (instant SIM) (built in Dr Hari Shroff Laboratory)^41^. On iSIM, Alexa488-labeled AlbiCpG (Ex: 488 nm) and LysoTracker Red DND-99 (Ex: 561 nm) was used for imaging for a long duration. Images were deconvolved and analyzed using home-built software^41^. Alternatively, confocal microscopy with deconvolution was conducted on a Leica SP8 workstation (Leica Microsystem, IL). On the Leica SP8 workstation, MEB fluorescence was monitored to image AlbiCpG (Ex: 561 nm; Em: 600–670 nm), confirming that there was minimal overlap in fluorescence between LysoTracker Green and MEB. Raw imaging results were deconvolved and analyzed on the Leica SP8 workstation.

### Proinflammatory factors

The concentrations of proinflammatory factors (TNFα, IL-6, and IL-12p40) were measured using ELISA (Thermo Fisher Scientific) according to the manufacturers’ instructions^13^. Cell culture medium from cells treated in vitro was collected at the specified time points post treatment. Sera of treated mice were collected at the specified time points post treatment. Medium or sera were diluted according to the manufacturers’ instructions.

### Splenomegaly

Female FVB mice (6–8 weeks. *n = *5) were injected with unconjugated CpG or AlbiCpG (5 nmol each mouse unless denoted otherwise). After 6 days, unless denoted otherwise, mice were weighted, spleens were collected, and the splenomegaly was assessed by determining the ratio of (spleen weight)/(mouse weight).

### Costimulatory factors on APCs

CD80 and CD86 were profiled on cell surfaces of RAW264.7 macrophages and BMDCs by flow cytometry. After treatment, cells were dissociated using non-enzymatic dissociation buffer. Dissociated cells were washed with PBS and resuspended in PBS for staining. To analyze costimulatory factors on LN-resident APCs, draining inguinal LNs were collected from mice treated with CpG or AlbiCpG (s.c. at the tail base), followed by physical tissue dissociation using sharp needles. Cells were pipetted for a few times to further dissociate cells, and filtered using a 40-µm cell strainer to remove tissue debris. Cells were washed twice using PBS, counted, and stained in PBS with dye-labeled antibodies at 4 °C for 30 min, followed by washing with PBS and flow cytometric analysis. Flow cytometry results were analyzed by using FlowJo V10 software.

### In vitro antigen cross presentation

CD11c^+^ BMDCs were isolated from female C57BL/6 mice (6–8 weeks) using positive selection beads (Miltenyi), and treated overnight with (OVA + AlbiCpG) vs. (OVA + CpG). OT-1 CD8^+^ T cells were isolated, labeled with CFSE, and added to co-culture with the above BMDCs. After 2 days, cells were stained with DAPI and anti-CD8-APC, and T cell proliferation was determined by divided viable (DAPI^−^) CD8^+^ cells in flow cytometry.

### AlbiVax uptake in LN-residing APCs

Dye-labeled AlbiVax or MSA (CpG: 2 nmol equivalent; antigen: 20 nmol equivalent; MSA: 20 nmol) were s.c. injected at the base of the tail of female C57BL/6j mice (6–8 weeks. *n = *6–8). LNs were isolated 1–3 days post injection. LNs were mechanically disrupted, pipetted, and filtered using 40-µm cell strainers. Cells were blocked with anti-CD16/CD32 in FCS buffer for 10 min, followed by staining for B cells (B220^+^), DCs (CD11c^+^), and macrophages (F4/80^+^), and analyzed by flow cytometry for AlbiVax or MSA fluorescence signal. Data were analyzed using FlowJo V10.

### Anti-OVA IgG1

Female C57BL/6 mice (6–8 weeks. *n* = 5) were vaccinated with AlbiCpG and OVA on day 0 and day 14. Peripheral blood was collected from vaccinated mice on day 21. Blood cells were precipitated by centrifugation and removed, and the resulting sera were enriched to measure anti-OVA IgG, IgG1, IgG2a, and IgM. Sera were diluted 1000 times for ELISA analysis.

### Tetramer staining and PD-1 expression on PBMCs

Mouse peripheral CD8^+^ T cells were stained for antigen-specific tetramer as described previously^15^. PE-conjugated H-2K^b^-SIINFEKL tetramer and H-2D^b^-ASMTNMELM tetramer (manufactured at the NIH Tetramer Core Facility) were used for tetramer staining of OVA-vaccinated mice and Adpgk-vaccinated mice, respectively. Briefly, mice were treated with AlbiVax on day 0 and day 14. Blood was collected from the treated mice on day 21. Blood cells were enriched by centrifugation. Red blood cells were lysed using ACK lysis buffer for 10 min at room temperature. Blood clots were removed using a filter. Cells were washed twice in PBS and cells were stained using Live/Dead Fixable Green Dead Cell Stain Kit for 10 min at room temperature. Staining was quenched and cells were washed with FCS buffer (PBS buffer with 0.1% FBS). Cells were then blocked with anti-CD16/CD32 for 10 min, followed by adding a dye-labeled antibody cocktail (CD3-PerCP Cy5.5, CD8-APC-Cy7, Tetramer-PE, PD-1-BV421) and staining at room temperature for 30 min. Cells were then washed, and 100 μL Cytofix was added into each well to resuspend cells, and cells were fixed at 4 °C for 20 min. Cells were then washed with Perm/Wash buffer, and resuspended for flow cytometric analysis. Flow cytometry was conducted on a BD LSRFortessa X-50 flow cytometer. Data were analyzed using FlowJo V10 version.

### Intracellular cytokine staining in T cells

Female C57BL/6 mice (6–8 weeks. *n = *5) were vaccinated with AlbiVax on day 0 and day 14. Peripheral blood was collected from vaccinated mice on day 21. Red blood cells were removed by incubating with ACK lysis buffer for 5 min and centrifugation for 10 min, and the obtained lymphocytes were transferred into U-bottom 96-well plate in 200 µL T cell culture media were: RPMI 1640 supplemented with 10% FBS, 100 U mL^−1^ penn/strep, 50 μM β-mercaptoethanol, 1x MEM non-essential amino acid solution, and 1 mM sodium pyruvate. Lymphocytes were pulsed with antigen epitopes (20 µg mL^−1^) for 2 h, followed by adding GolgePlug Protein Transport Inhibitor containing brefeldin A. Cells were then placed in a culture incubator for 4 h. Next, cells were incubated with anti-CD16/CD32 for 10 min at room temperature, then the cells were stained with anti-CD8-APC-Cy7 and DAPI for 20 min at room temperature. Cells were washed and subsequently fixed using Cytofix (BD Biosciences), washed, and permeabilized in 200 µL Cytoperm solution (BD Biosciences) according to the manufacturer’s instructions. Cells were washed using Perm/Wash Buffer (BD Biosciences). Permeabilized cells were then stained using anti-IFNγ-PE and anti-TNFα-FITC according to the manufactor’s guidance. Stained cells were then washed and resuspended for flow cytometric analysis. Flow cytometry was conducted on a BD LSRFortessa X-50 flow cytometer. Data were analyzed using FlowJo V10 version.

### Immune memory

Female C57BL/6 mice (6–8 weeks. *n = *6–8) were vaccinated as described above. Peripheral blood was collected to analyze antigen-specific CD8^+^ T cells and memory T cells. Immune memory was analyzed by flow cytometric analysis of peripheral lymph node homing receptor, CD62L, and adhesion molecule, CD44. Briefly, red blood cells were lysed using ACK lysis buffer, and blood cells were then collected by centrifugation and washing with FCS buffer (PBS buffer with 0.1% FBS). Cells were then blocked with anti-CD16/CD32 in FCS buffer for 10 min, followed by adding dye-labeled antibody cocktail (CD8-APC-Cy7, CD44-PE-Cy5, CD62L-FITC, and dead cell-staining DAPI) and staining at room temperature for 30 min. Cells were then washed, and 100 μL Cytofix was added into each well to resuspend cells. Cells were allowed for fixation at 4 °C for 20 min. Cells were then washed with Perm/Wash buffer, and resuspended for flow cytometric analysis. Central memory CD8^+^ T cells were analyzed as CD44^hi^CD62L^hi^ CD8^+^ T cells; effector memory CD8^+^ T cells had variable to low levels of CD62L and high CD44 levels; and naive CD8^+^ T cells had high levels of CD62L and low to intermediate levels of CD44. Flow cytometry was conducted on a BD LSRFortessa X-50 flow cytometer. Data were analyzed using FlowJo V10 version.

1° tumor challenge was conducted by s.c. inoculation of tumor cells (3 × 10^5^) on the right shoulder. Tumor sizes were monitored every 3 days thereafter. If applicable, 2° tumor challenge was conducted by s.c. inoculation of tumor cells (1 × 10^6^) on the right flank of mice that survived the 1° tumor challenge. Tumor sizes were again monitored every 3 days thereafter. Mice were killed if any dimension of the tumor was close to or already exceeded 2 cm. Tumor volume was calculated using the following formula:

$$\rm{Volume} = \left( {length \times width^2} \right)/2$$

Results were analyzed using GraphPad Prism 7 (La Jolla, CA).

### Plasmid transfection of tumor cells

B16F10 and MC38 cells were transfected with plasmid pcDNA3.1 (+)-*Neo*-*fLuc* for firefly luciferase expression. Briefly, 70% confluent cells were transfected using Lipofectamine 2000 (Invitrogen) according to the manufacturer’s instructions. Two days after transfection, cells were treated with Geneticin (0.4 mg mL^−1^) to screen cells that stably express firefly luciferase for a total of 4 weeks. The resulting cells were confirmed for firefly luciferase expression using bioluminescence imaging. Cells that expressed firefly luciferase were further cultured in medium containing Geneticin (0.4 mg mL^−1^).

### Tumor immunotherapy

For primary tumor models, female C57BL/6 mice (6–8 weeks. The Jackson Laboratory. *n = *6–8) were s.c. inoculated with EL4 cells, or EG7.OVA cells, B16F10 cells, MC38 cells on the shoulder. Tumor growth was monitored by caliper measurement. Mice were killedd when the maximal dimension of tumor reached about 2 cm. For a lung metastatic melanoma model, female C57BL/6 mice (6–8 weeks) were i.v. injected with B16F10-*fLuc* cells or MC38 cells (1 × 10^5^ per mouse).

To study antigen specificity of immunotherapy, 2 × 10^5^ EL4 lymphoblastoma cells and 2 × 10^5^ EG7.OVA (OVA expressing) were respectively inoculated on to the left and right shoulders of C57BL/6 mice (6–8 weeks). Four groups of mice (6–7 mice per group) were respectively vaccinated with (1) PBS, (2) CpG and OVA, (3) AlbiGpC and OVA, and (4) AlbiCpG and OVA (doses: 2 nmol CpG equivalents, 20 µg OVA), by s.c. injection in 50 µL volume at the base of the tail on day 3 and day 9 post-tumor inoculation. Tumor sizes and mouse weights were monitored every 3 days. Mice were killed on day 19 when one dimension of most EL4 tumors was close to or already exceeded 2 cm. The tumor volume was calculated and analyzed as described above.

A combination of immunotherapy (AlbiVax and anti-PD-1, Clone RMP1–14, Bio X cell) and chemotherapy (Abraxane) for melanoma treatment was studied in both a primary melanoma model and a lung metastatic melanoma model. In primary tumor model, 3 × 10^5^ B16F10 cells in 100 μL PBS were s.c. injected into the right shoulder of C57BL/6 mice. On day 6 post-tumor inoculation, when established tumors were palpable, mice were randomly divided into six groups (6–10 mice per group) for treatment. The dose is 1.4 nmol CpG equivalents, 20 μg Trp2 or AbTrp2, 200 μg anti-PD-1 (Bio X Cell, Inc., NH), and 20 mg kg^−1^ Abraxane (Celgene, MD). Vaccines were injected s.c. at the base of tail, anti-PD-1 was injected intraperitoneally, and Abraxane was injected i.v. Maintenance treatment was performed at the following doses: anti-PD-1 every 3 days for a total of five times, vaccines every 6 days for a total of three times, and Abraxane every 6 days for a total of three times. Tumor sizes and mouse weights were monitored every 3 days. Mice were killed when one dimension of a tumor exceeded 2 cm or ulceration developed. The tumor volume was calculated and analyzed as described above. Results from two independent studies were pooled together. Results were analyzed using GraphPad Prism 7 (La Jolla, CA).

In the lung metastatic B16F10 study, 1 × 10^5^ B16F10-*fLuc* cells were injected i.v. into C57BL/6 mice. On day 6 when lung metastasis is established, treatment was initiated (vaccine: 2 nmol CpG equivalents, 20 µg Ag, every 6 days; anti-PD-1: 200 µg, every 3 days). The growth of luciferase transgenic tumors was monitored by bioluminescence imaging after i.p. injection of an aqueous solution of D-luciferin (0.1 ml, 30 mg mL^−1^, GoldBio, St. Louis, MO) on an IVIS Lumina (Caliper Life Sciences). Bioluminescence in regions of interest (ROI) were quantified as total flux. Mice were killed if they developed severe morbidity. Mice were all killed on day 20 post inoculation, and lungs were collected to enumerate the counts of lung metastatic nodules and measure lung weights.

In lung metastatic-like MC38 tumor studies, 1 × 10^5^ MC38 cells were injected i.v. into C57BL/6 mice. On day 10 when lung metastasis is established, treatment was initiated (vaccine: 2 nmol CpG equivalents, 20 µg Ag, every 6 days; anti-PD-1: 200 µg, every 3 days). Mice were killed if they developed severe morbidity. At the end of study, mice were i.p. injected with FDG (3.7 Mbq). Representative mice were scanned by PET/CT on a nanoScan system (Mediso), and all mice were killed, and lungs collected to measure lung weights and radioactivity by γ-counting.

In lymphocyte depletion, female C57BL/6 mice (6–8 weeks) were *s.c*. inoculated with EG7.OVA or MC38 cells (3 × 10^5^) on the right shoulder. On day 6 when tumors were established, mice were divided into five groups to have comparable tumor volumes (*n* = 5). Five groups of mice were respectively vaccinated with PBS in group (1), and AlbiVax (2 nmol CpG equivalents and 20 µg OVA or AlbiAdpgk) in groups (2–5), by s.c. injection in 50 µL PBS at the base of the tail on day 6, 12, and 18 post-tumor inoculation. Meanwhile, on days 6, 9, 12, 15, and 18 post-tumor inoculation, mice in groups (2–5) were also intraperitoneally injected with PBS in group (2), anti-CD4 in group (3), anti-CD8 in group (4), and anti-NK1.1 in group (5) (antibody dose: 200 µg per mouse). Tumor sizes and mouse weights were monitored every 3 days. Mice were killed when any tumor dimension was close to or already exceeded 2 cm. The tumor volume was calculated and analyzed as described above.

### Data availability

The authors declare that all the data related with this study are available within the paper or can be obtained from the authors on request.

## Electronic supplementary material

Description of Additional Supplementary Files

Supplementray Information

Supplementary Movie 1

Supplementary Movie 2

Supplementary Movie 3

Supplementary Movie 4

Peer Review File
