# Supplementary material for: Albumin/vaccine nanocomplexes that assemble in vivo for combination cancer immunotherapy
Source: Nat Commun. 2017 Dec 5;8:1954. doi: 10.1038/s41467-017-02191-y (PMC5715147; doi:10.1038/s41467-017-02191-y)
Supplement: Supplementary file 1 — Description of Additional Supplementary Files [file 41467_2017_2191_MOESM1_ESM.pdf]

Description of Additional Supplementary Files

File Name: Supplementary Movie 1
Description: PET imaging of LN delivery of AlbiCpG in mice at 6h post injection. AlbiCpG was radiolabelled with 64Cu via chelation with NOTA.

File Name: Supplementary Movie 2
Description: Light-sheet fluorescence imaging of the distribution of AlbiCpG in mouse LNs. AlbiCpG was labeled with Alexa488.

File Name: Supplementary Movie 3
Description: Light-sheet fluorescence imaging of subcapsular AlbiCpG in mouse LNs. Green: AlbiVax; blue: cell nucleus stained by DAPI; red: B cells.

File Name: Supplementary Movie 4
Description: Super-resolution confocal imaging intracellular AlbiCpG in one single APC. Green: LysoTracker Green; red: AlbiCpG-Alexa555.
