# Supplementary material for: Albumin/vaccine nanocomplexes that assemble in vivo for combination cancer immunotherapy
Source: Nat Commun. 2017 Dec 5;8:1954. doi: 10.1038/s41467-017-02191-y (PMC5715147; doi:10.1038/s41467-017-02191-y)
Supplement: Supplementary file 2 — Supplementray Information [file 41467_2017_2191_MOESM2_ESM.pdf]

a

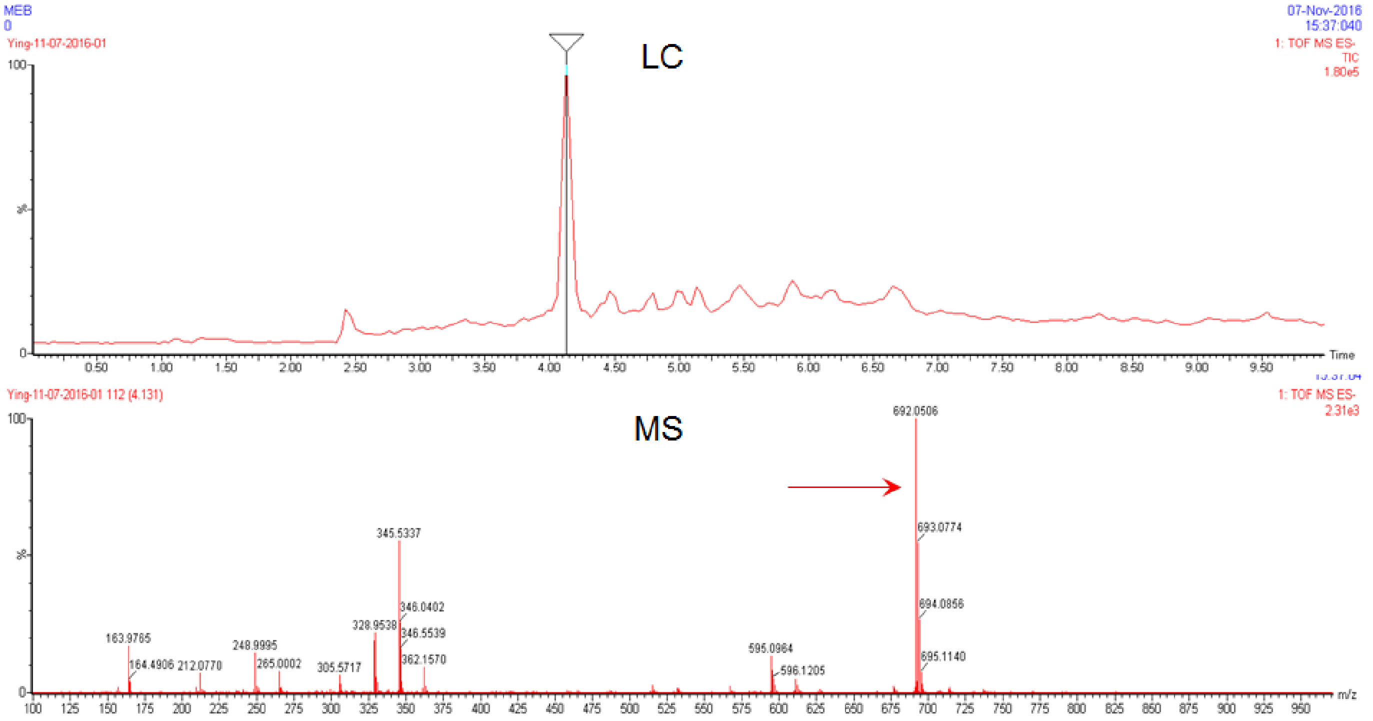
MEB
0
Ying-11-07-2016-01
07-Nov-2016
15:37:040
1: TOF MS ES-
TIC
1.80e5
LC
Time
Ying-11-07-2016-01 112 (4.131)
1: TOF MS ES-
2.31e3
MS
692.0506
693.0774
694.0856
695.1140
595.0964
596.1205
345.5337
346.0402
346.5539
362.1570
328.9538
305.5717
248.9995
265.0002
163.9765
164.4906
212.0770
m/z

b

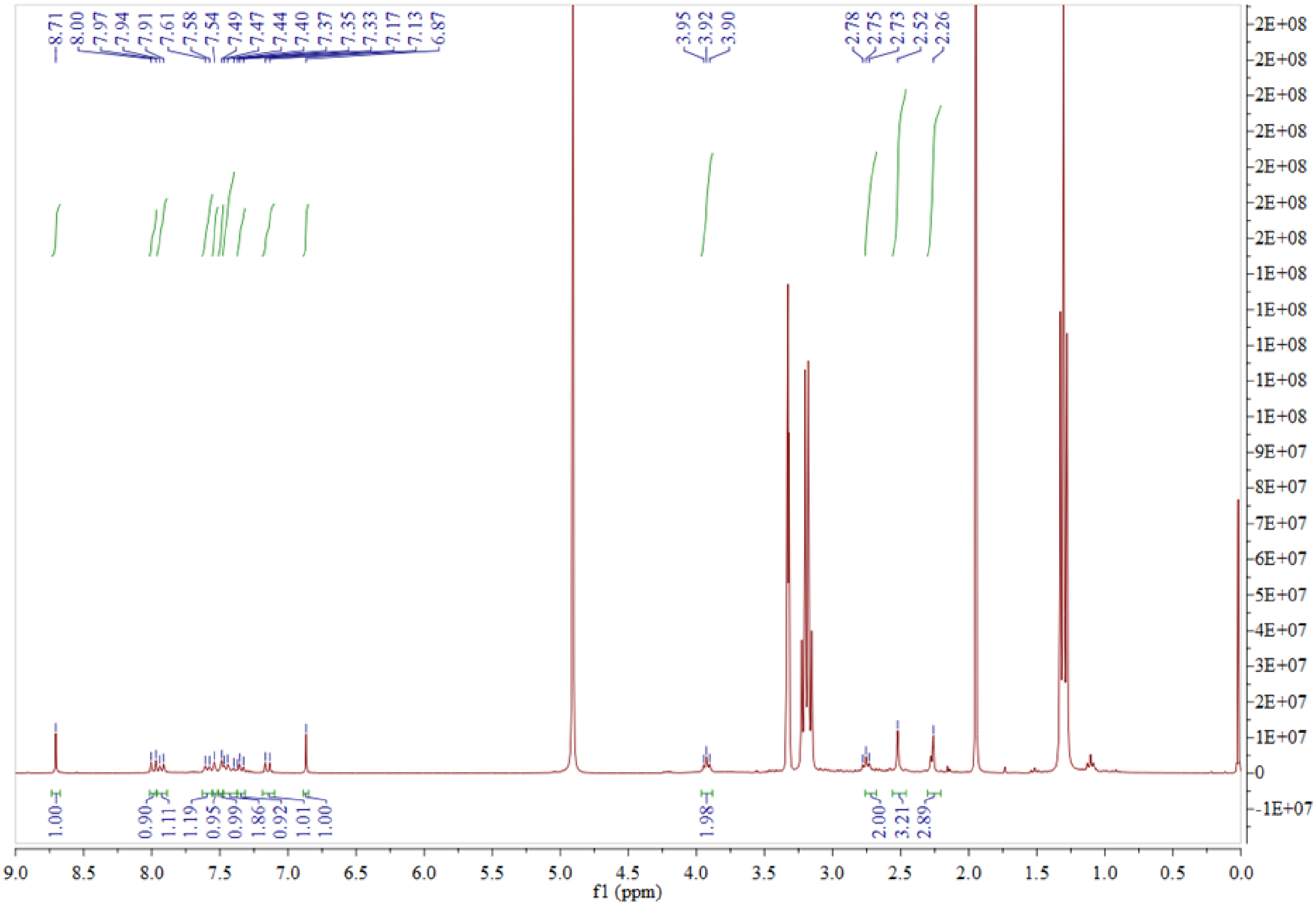
8.71
8.00
7.97
7.94
7.91
7.61
7.58
7.54
7.49
7.47
7.44
7.40
7.37
7.35
7.33
7.17
7.13
6.87
3.95
3.92
3.90
2.78
2.75
2.73
2.52
2.26
1.00
0.90
1.11
1.19
0.95
0.99
1.86
0.92
1.01
1.00
1.98
2.00
3.21
2.89
f1 (ppm)

c

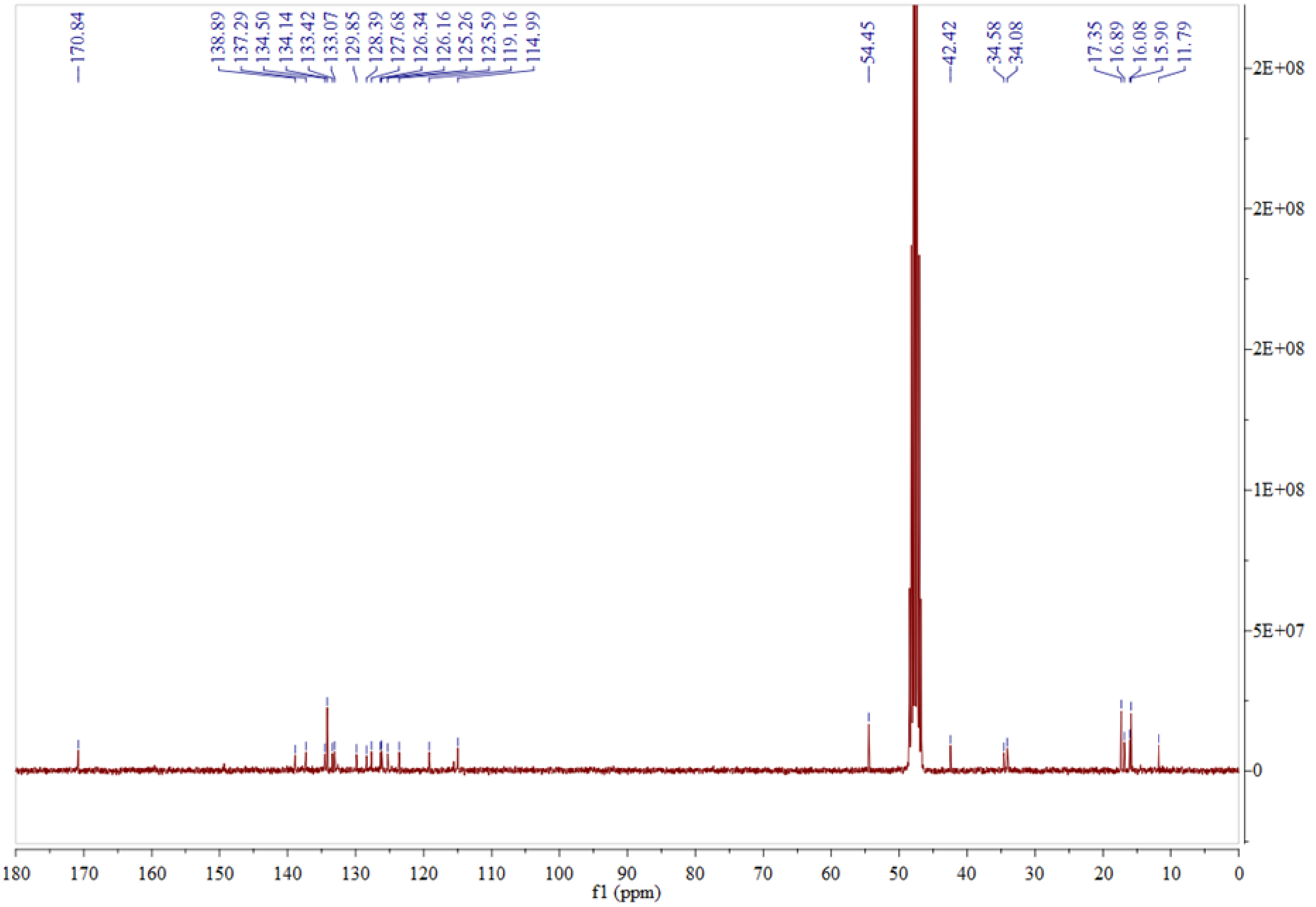

Supplementary Fig. 1. Characterization of MEB. To synthesize maleimide-functionalized EB derivative (MEB), di-tert-butyl dicarbonate was used to protect one end of tolidine in dichloromethane to afford Boc-tolidine, which was further reacted with1-amino-8-naphthol-2,4-disulfonic acid monosodium salt, followed by Boc deprotection using trifluoroacetic acid, to obtain EB-NH_2. Next, 3-maleimidopropionic acid (51.0 mg, 0.3 mmol), PyBOP (104.0 mg, 0.2 mmol) and DIPEA (130 mg, 1.0 mmol) were added to a solution of EB-NH_2 (54.1 mg, 0.1 mmol) in dry DMF. The mixture was vigorously stirred at room temperature for 24 h and monitored by the HPLC. After solvent evaporation under reduced pressure, the residue was purified by a Waters Xterra C-18 chromatography column running a linear gradient from 5% to 65% acetonitrile (0.1% TFA). The yield is ca. 75%. (a) LC-MS analysis of MEB. (b) NMR spectrum of MEB. ^{1}H NMR (300 MHz, MeOD) δ 8.71 (s, 1H), 7.99 (d, *J* = 9.9 Hz, 1H), 7.93 (d, *J* = 8.5 Hz, 1H), 7.59 (d, *J* = 8.7 Hz, 1H), 7.54 (s, 1H), 7.49 (s, 1H), 7.48 – 7.37 (m, 2H), 7.35 (t, *J* = 6.7 Hz, 1H), 7.15 (d, *J* = 9.9 Hz, 1H), 6.87 (s, 1H), 3.92 (t, *J* = 6.8 Hz, 2H), 2.75 (t, *J* = 6.8 Hz, 2H), 2.52 (s, 3H), 2.26 (s, 3H). (c) ^{13}C NMR (75 MHz, MeOD) δ 170.84, 138.89, 137.29, 134.50, 134.14, 133.42, 133.07, 129.85, 128.39, 127.68, 126.34, 126.16, 125.26, 123.59, 119.16, 114.99, 54.45, 42.42, 34.58, 34.08, 17.35, 16.89, 16.08, 15.90, 11.79.

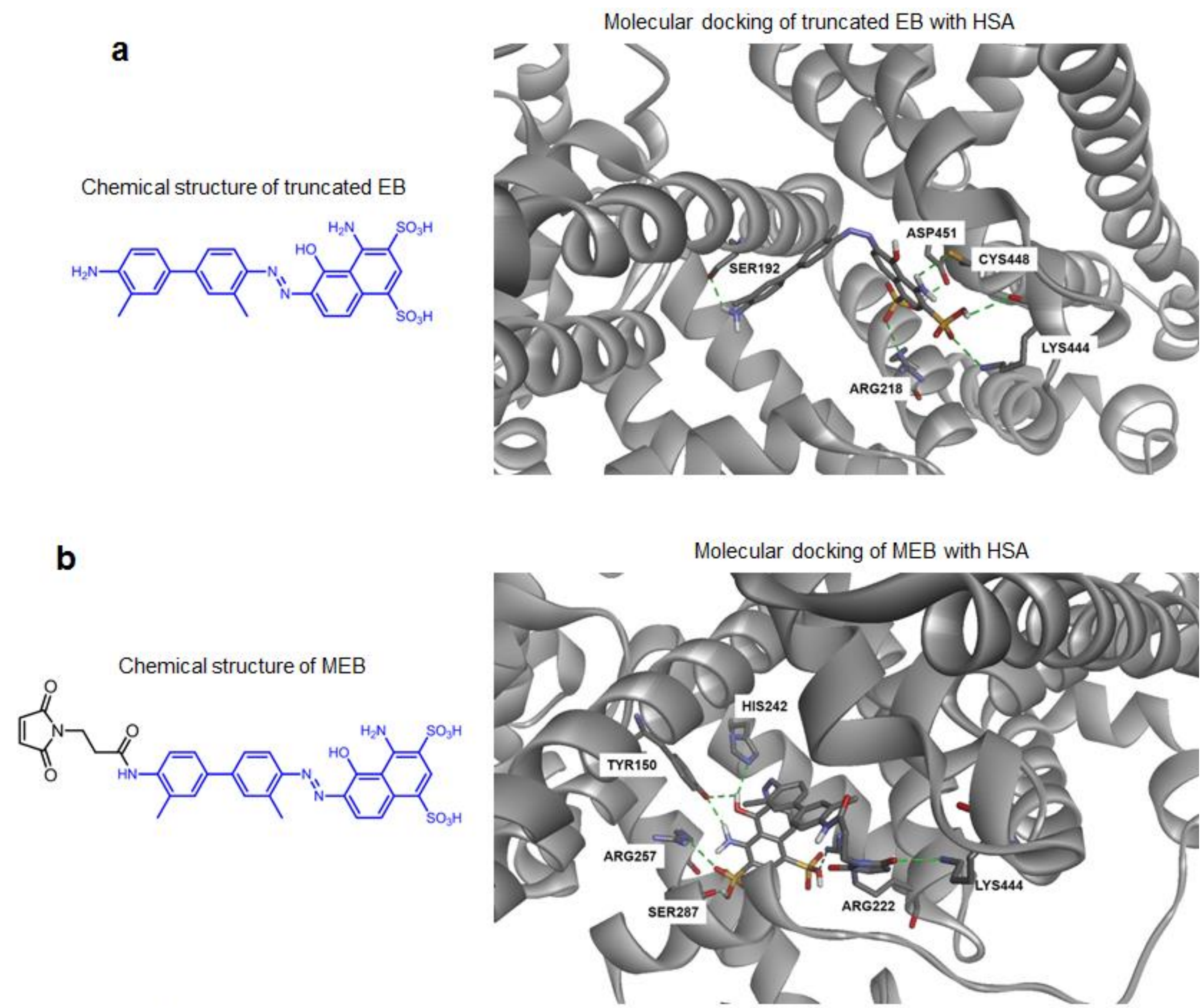

c

	$\Delta G_{binding}{}^{a}$ (kcal/mol)	$K_i{}^{b}$
Truncated EB with HSA	−9.13	203.80 nM
MEB with HSA	−9.38	133.00 nM

Supplementary Fig. **2.** Molecular docking and binding prediction. (**a**) Chemical structure of truncated EB (tEB) and predicted structure of tEB/HSA complex. HSA (grey) is represented as a solid ribbon. tEB and the residues (SER192, ARG218, LYS444, CYS448, and ASP451) in the binding site of HSA are represented in stick format. Hydrogen bonds of tEB with interacting amino acid residues in HSA are shown by green dashed lines. (**b**) Chemical structure of MEB and predicted structure of MEB/HSA complex. MEB and the residues (TYR150, ARG222, HIS242, ARG257, SER287, and LYS444) in the binding site of HSA are in stick representation. Hydrogen bonds of MEB with interacting amino acid residues in HSA are shown by green dashed lines. Docking figures were prepared with DSViewPro (BIOVIA). (**c**) Predicted $\Delta G_{binding}$ and K_i. [a]Binding free energy. [b]Inhibition constant (T = 298.15 K).

a. CpG

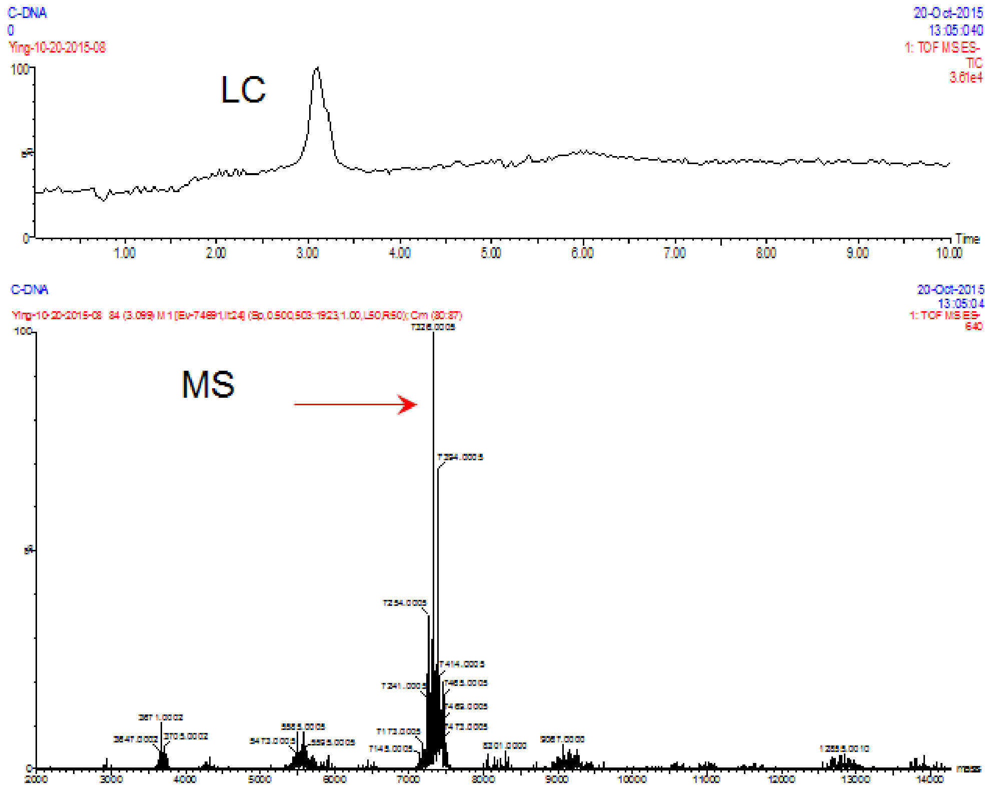

b. MEB-CpG

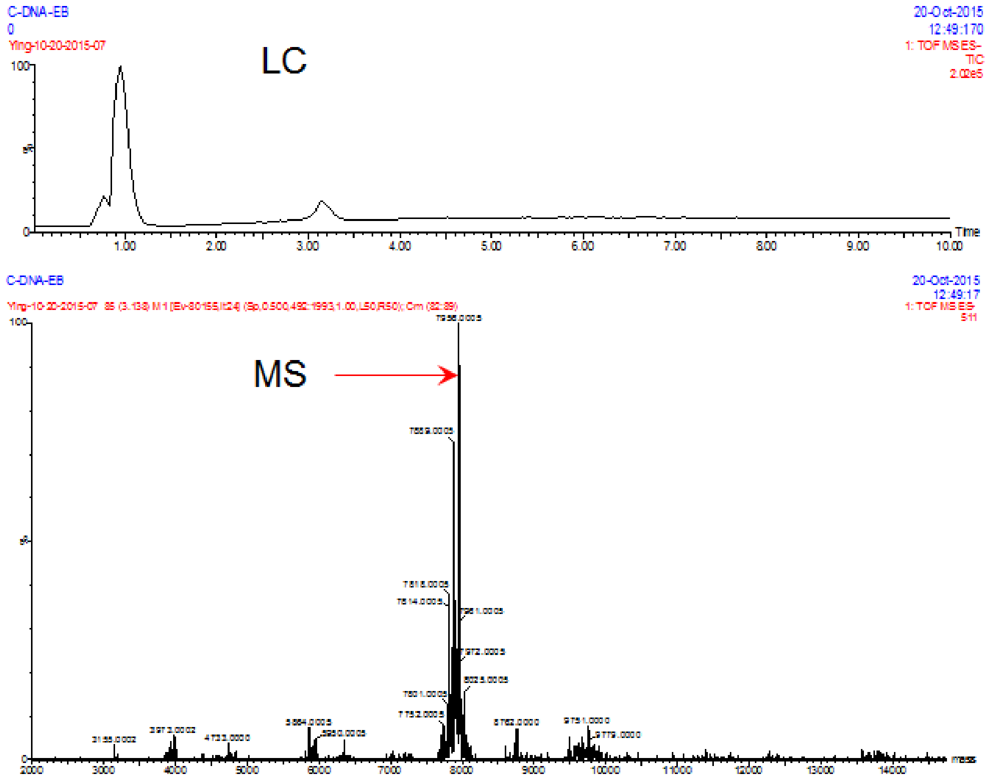

Supplementary Fig. 3. LC-MS results of CpG and MEB-CpG. CpG1826 (TCCATGACGTTCCTGACGTT) was used. MEB-CpG was synthesized using MEB and terminal thiol-modified CpG. Thiol-modified DNA was pretreated with DTT (0.1 M) in PBS (37 °C, 1 h) to cleave the dithiol bond, followed by desalting using a NAP5 column in sodium ascorbate buffer (0.1 %) to remove DTT and the thiol-appending small fragment cleaved from DNA. The resulting DNA (200 nmol) was mixed with MEB (1 mg) in 2 mL sodium ascorbate buffer (0.1 %) in PBS and reacted at room temperature for 30 min. The resulting product was purified again using a NAP5 column to remove excess MEB, and quantified by UV absorbance at 260 nm (where MEB has negligible absorption compared with CpG) or by weighing lyophilized products in the case of synthesis at a relatively large scale.

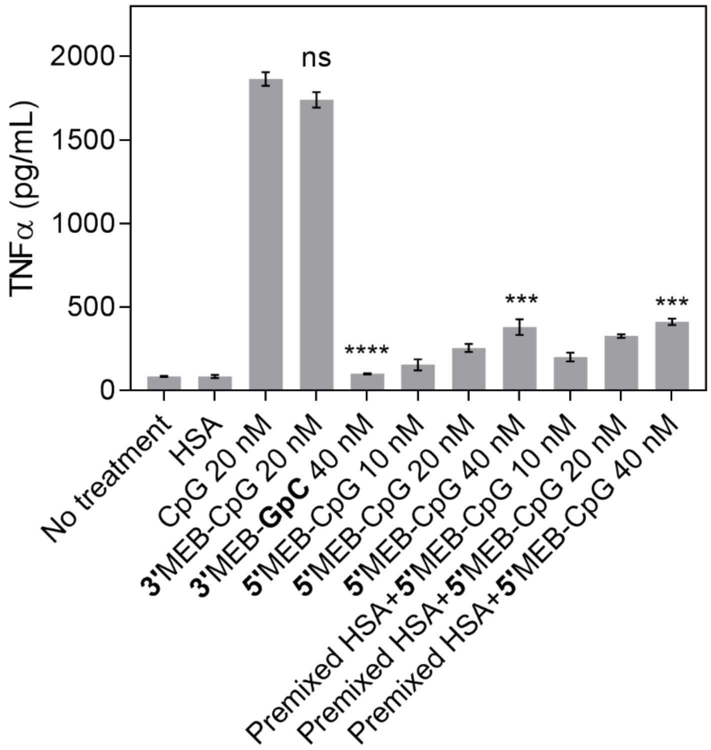

Supplementary Fig. 4. Effect of MEB modification sites on the immunostimulatory potency of CpG derivatives. ELISA analysis of the culture medium of *in vitro* RAW264.7 macrophages demonstrate that MEB-CpG with MEB modified on the 3'-end of CpG maintained its immunostimulatory activity either alone or premixed with human serum albumin (HSA). MEB-CpG conjugate with MEB modified on the 5'-end of CpG dramatically abrogated the immunostimulatory potency of CpG. Further, AlibiGpC control was inactive for immunostimulation, ruling out the immunogenicity of the MEB moiety. Data shown mean ± s.e.m. of 3 independent experiments. ****$p < 0.0001$, ***$p < 0.001$, ns: not significant ($p > 0.05$) by one-way ANOVA with Bonferroni post-test. Significance was compared between the group of (CpG 20 nM) and the corresponding other groups.

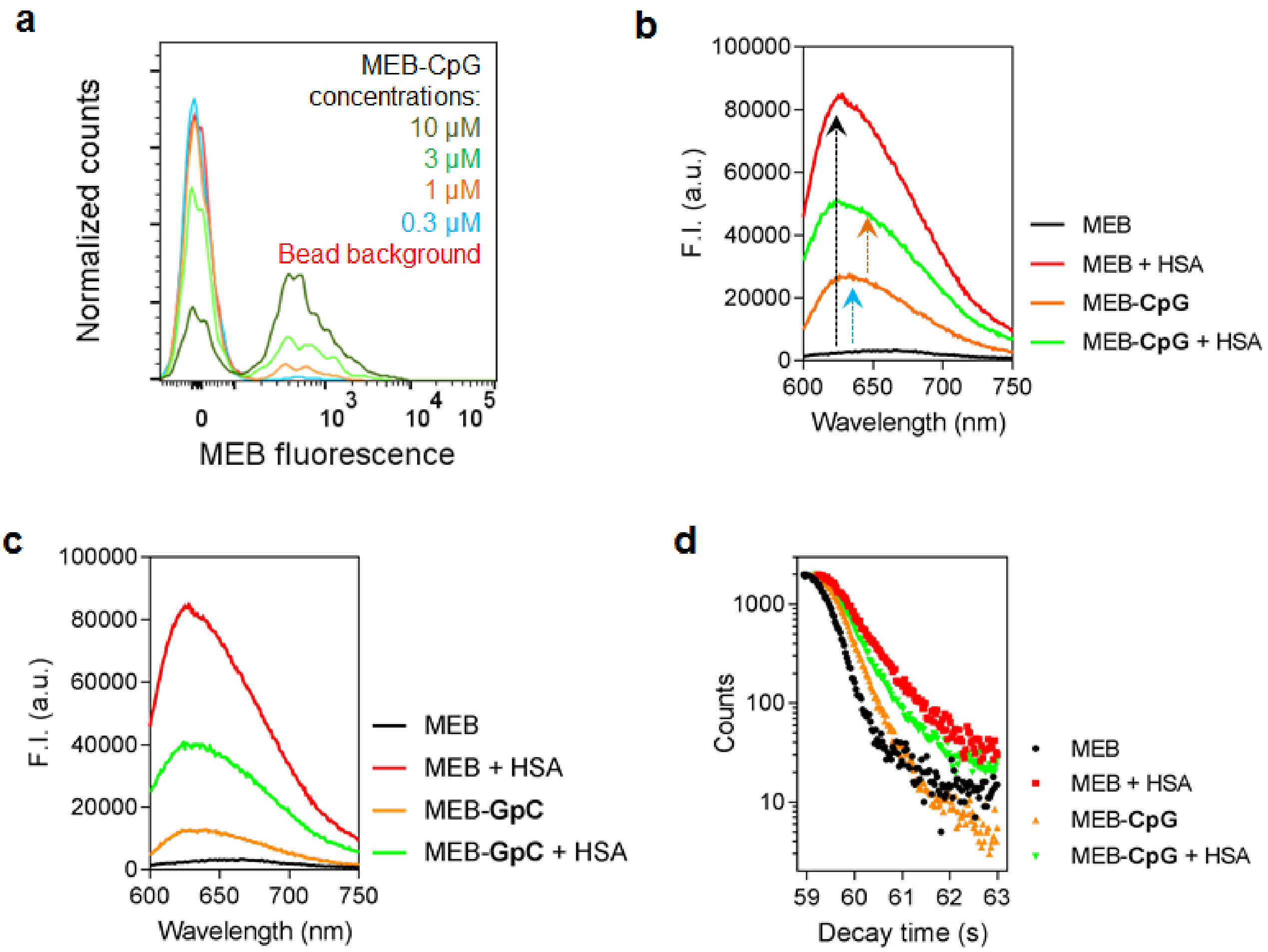

Supplementary Fig. 5. Recovery of MEB fluorescence and prolonged MEB fluorescence lifetime upon albumin binding of MEB-CpG. (a) Flow cytometry results showing the MEB fluorescence of HSA-coated beads in the presence of MEB-CpG, indicating binding of MEB-CpG to HSA. (b-d) Fluorescence of free MEB was enhanced upon conjugation with DNA (CpG and GpC), and the fluorescence of MEB in MEB-CpG (b) or MEB-GpC (c) was further enhanced in the presence of HSA, suggesting the binding of MEB-CpG with HSA. The fluorescence of MEB and MEB-CpG showing the blue shift of the fluorescence spectrum of MEB-CpG relative to free MEB. (Ex: 530 nm. MEB or equivalent: 1 μM. HSA: 10 μM) (d) Prolonged FL lifetime in the presence of HSA again suggests binding of AlbiCpG with HSA. (Ex: 480 nm. MEB or equivalent: 1 μM. HSA: 10 μM)

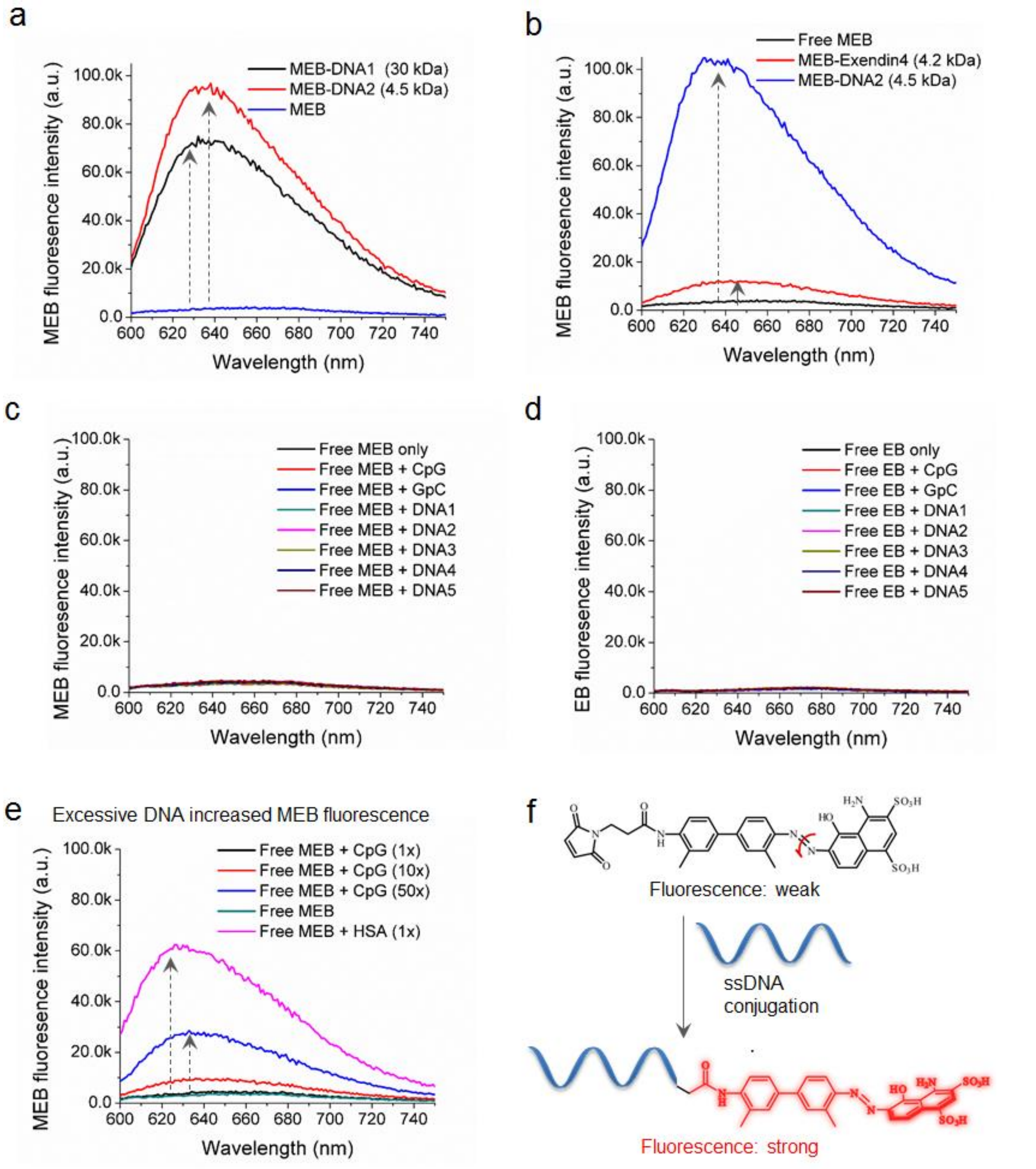

Supplementary Fig. 6. MEB fluorescence was recovered in covalently conjugated MEB with DNA independent of DNA length, at a dramatically greater degree than that of MEB-peptide covalent conjugates or mixture of free MEB with free DNA. (a) Spectra of MEB fluorescence demonstrate that MEB fluorescence of MEB-DNA conjugates was not dramatically influenced by the length of DNA in the case of a 15-mer DNA

(MW: ~4.5 kD) and a 90-mer DNA (MW: ~30 kD). (b) Spectra of MEB fluorescence showed that compared with peptide (Exendin4, MW: ~4.2 kDa) of comparable MW, the MEB fluorescence in MEB-DNA2 conjugate was activated at a dramatically higher degree. (c-e) Spectra of MEB fluorescence showed no fluorescence activation by non-covalent interactions of DNA and MEB (c) and parent Evans blue (EB) (d), at 1:1 molar ratio in the mixture of free MEB/EB and a panel of free ssDNA. In the mixture of free MEB and free DNA, MEB fluorescence can only be slightly increased in the presence of excessive DNA (e). (f) Schematic of MEB fluorescence activation upon conjugation with ssDNA. [Concentration of MEB or MEB-DNA conjugate: 1 μM; concentration of DNA: 1 μM unless noted otherwise in (e); HSA: human serum albumin; Ex: 550nm; Em: 600 nm-750 nm]

Sequences of MEB-DNA:

- CpG: TCCATGACGTTCCTGACGTT-MEB
- GpC: TCCATGAGCTTCCTGAGCTT-MEB
- DNA1: CGCTGAAAAACGTCAGGAACGTCATGGAAAAAAAACGTCAGGAACGTCATGGAAAAAAAACGTCAGGAACGTCATGGAAAAGTCA-MEB
- DNA2: GGTTGGTGTGGTTGG-MEB
- DNA3: AGCGTCGAATACCACTACACTTACACCAACTCTATCATCTCCCTTATAGACCACGAGCTCCATTAG-MEB
- DNA4: ATCTAACTGCTGCGCCGCCGGGAAAATACTGTACGGTTAGA-MEB
- DNA5: TGACGTAGGTTGGTGTGGTTGGGGCGTCA-MEB

MEB-Exendin4 peptide conjugate: MEB-CHGEGTFTSDLSKQMEEEAVRLFIEWLKNGGPSSGAPPPS[1]

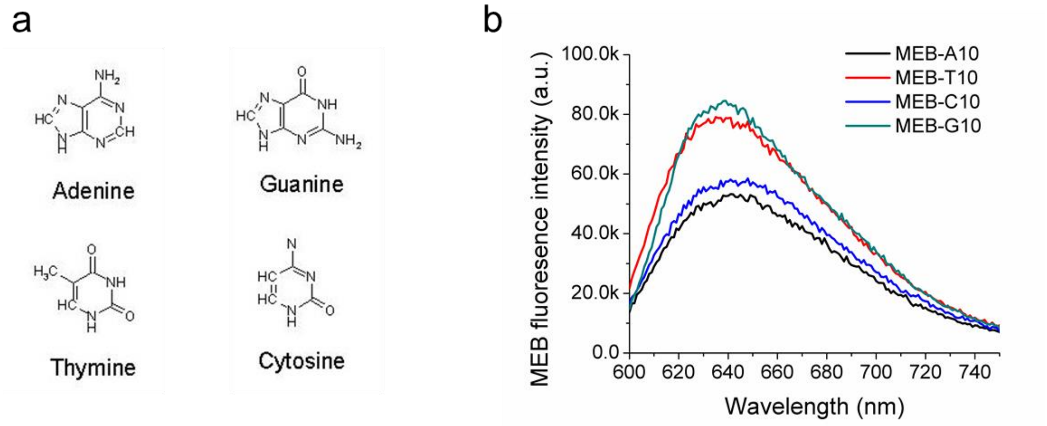

Supplementary Fig. 7. Partial activation of MEB fluorescence in MEB-DNA conjugates is independent of deoxynucleotides, despite up to 25% differences between fluorescence intensities. (a) Chemical structures of 4 nucleosides in DNA. (b) Spectra of MEB fluorescence showed that in four 10-mer ssDNA, T10 and G10 activated MEB fluorescence at a slightly higher degree than A10 and C10. [Concentration of MEB-DNA conjugate: 1 μM; Ex: 550nm; Em: 600 nm-750 nm]

Sequences of MEB-DNA conjugates:

- MEB-A10: AAA AAA AAA A-MEB
- MEB-T10: TTT TTT TTT T-MEB
- MEB-C10: CCC CCC CCC C-MEB
- MEB-G10: GGG GGG GGG G-MEB

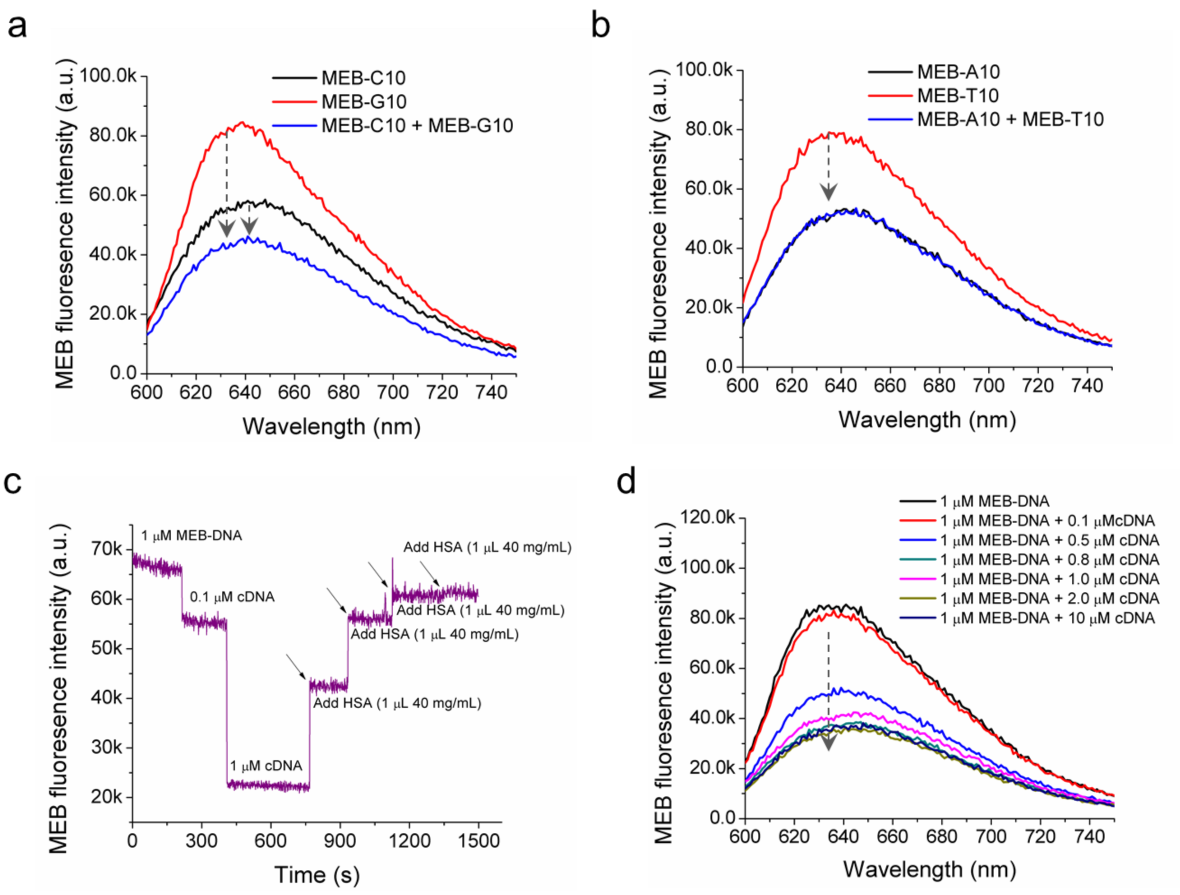

Supplementary Fig. 8. Formation of double-stranded DNA (dsDNA), by hybridization with the complementary DNA (cDNA), decreased the MEB fluorescence intensity of MEB-ssDNA conjugates. We hypothesize that this is because formation of dsDNA interfered with the electrostatic interaction between ssDNA and the MEB moiety in MEB-ssDNA conjugates. (a, b) Spectra of MEB fluorescence showed that the MEB fluorescence intensities of MEB-DNA were decreased by hybridization with cDNA. (Final total ssDNA equivalent: 1 μM). (c) Kinetic study of the decrease of MEB fluorescence intensity of MEB-DNA by adding cDNA, and the recovery of MEB fluorescence intensity by gradually adding HSA. (Ex: 550nm; Em: 680 nm) (d) Spectra of MEB fluorescence showed that the MEB fluorescence intensities of MEB-DNA were gradually decreased by hybridization with cDNA in a series of increasing concentrations. Sequences of MEB-DNA used in (c, d): TCCATGACGTTCCTGACGTT-MEB.

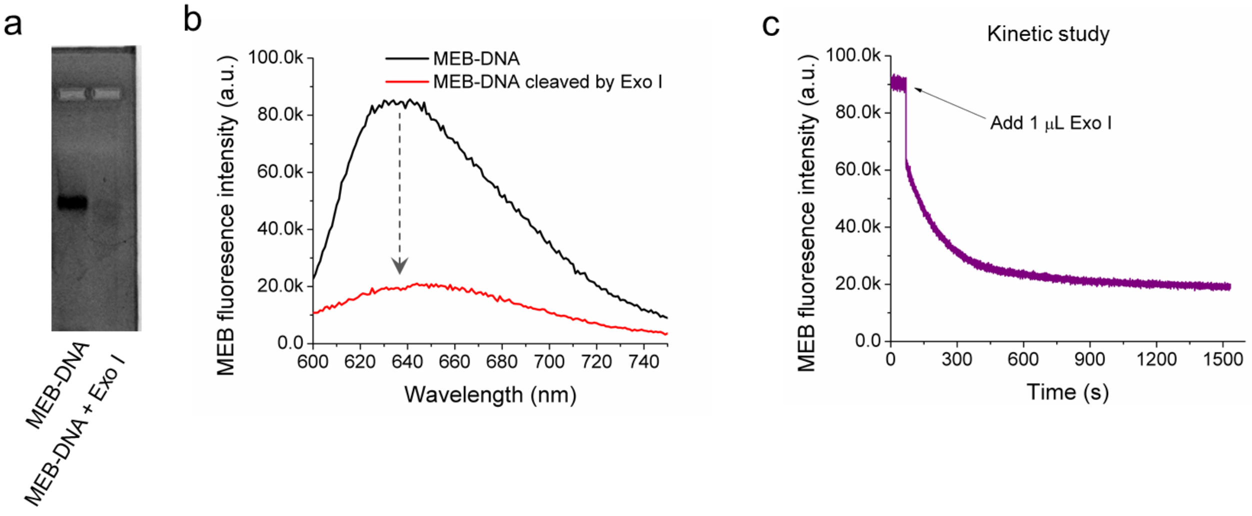

Supplementary Fig. 9. Cleavage of DNA in MEB-DNA conjugates decreased their MEB fluorescence. (a) An agarose gel electrophoresis image showing the cleavage of DNA in MEB-DNA conjugate by DNA Exonuclease I (Exo I). Exo I degrades ssDNA and releases deoxyribonucleoside 5'-monophosphates in a stepwise manner and eventually leaves 5'-terminal dinucleotides intact. Spectra (b) and real-time fluorescence intensities (Ex: 550 nm; Em: 680 nm) of MEB fluorescence (c) showed that the MEB fluorescence intensities of enzymatically cleaved MEB-DNA were decreased, compared to intact MEB-DNA. Sequences of MEB-DNA: TCCATGACGTTCCTGACGTT-MEB. [Concentration of MEB-DNA conjugate: 1 μM; Ex: 550nm; Em: 600 nm-750 nm]

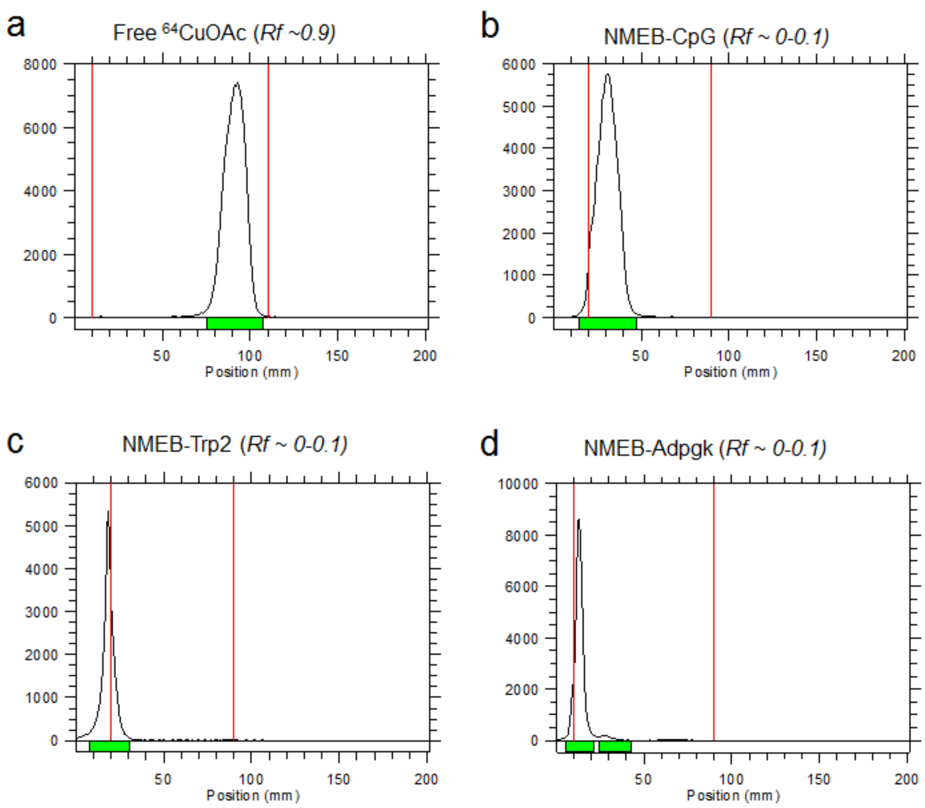

Supplementary Fig. 10. Instant thin layer chromatography (iTLC) graphs showing the labeling of CpG, Adpgk, and Trp2 with ^{64}Cu *via* NMEB. Rf: retention factors.

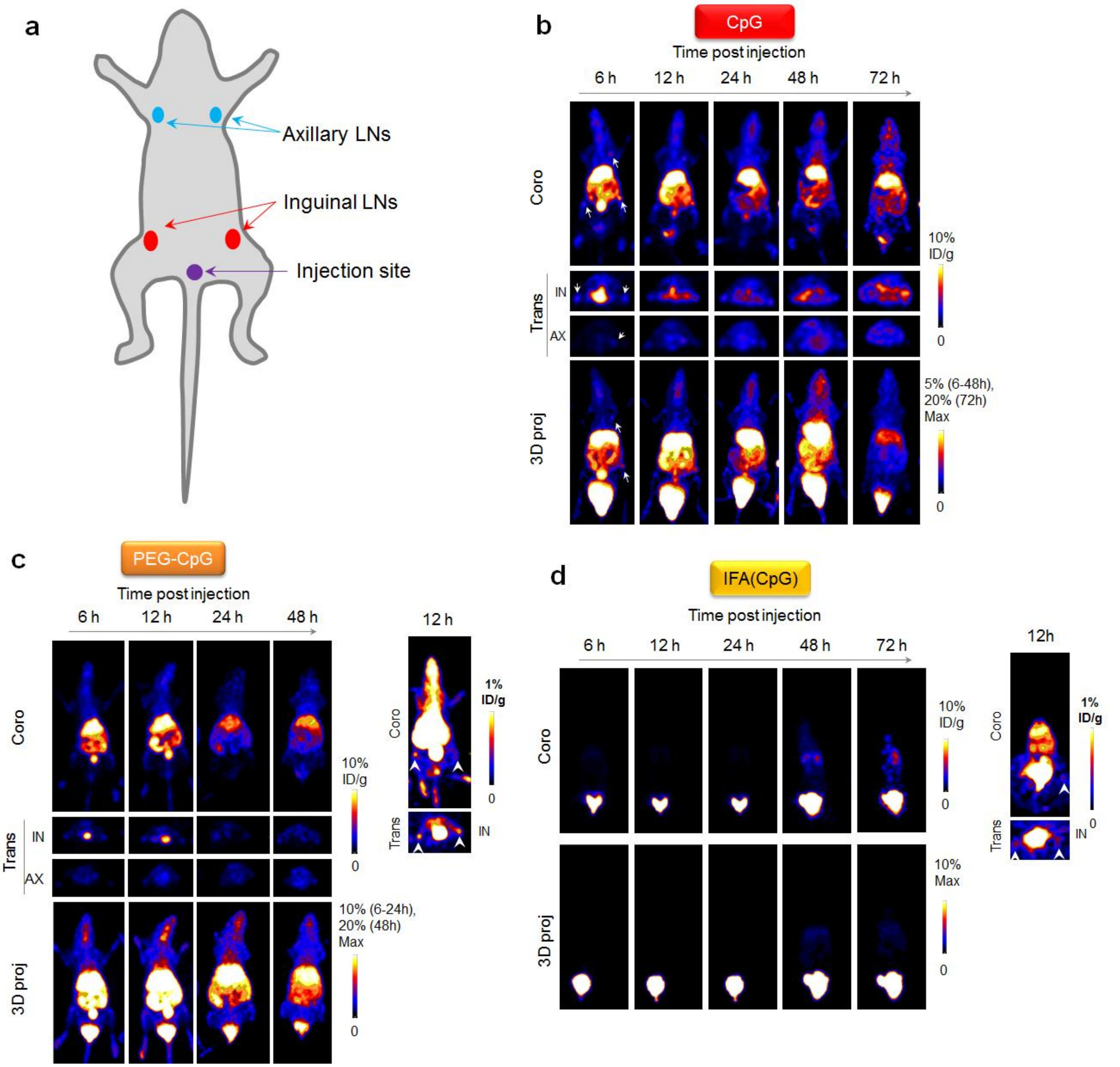
a
Axillary LNs
Inguinal LNs
Injection site
b
CpG
Time post injection
6 h
12 h
24 h
48 h
72 h
Coro
Trans
IN
AX
3D proj
10% ID/g
0
5% (6-48h), 20% (72h) Max
0
c
PEG-CpG
Time post injection
6 h
12 h
24 h
48 h
12 h
Coro
Trans
IN
AX
3D proj
10% ID/g
0
10% (6-24h), 20% (48h) Max
0
1% ID/g
0
d
IFA(CpG)
Time post injection
6 h
12 h
24 h
48 h
72 h
12h
Coro
3D proj
Trans
IN
10% ID/g
0
10% Max
0
1% ID/g
0

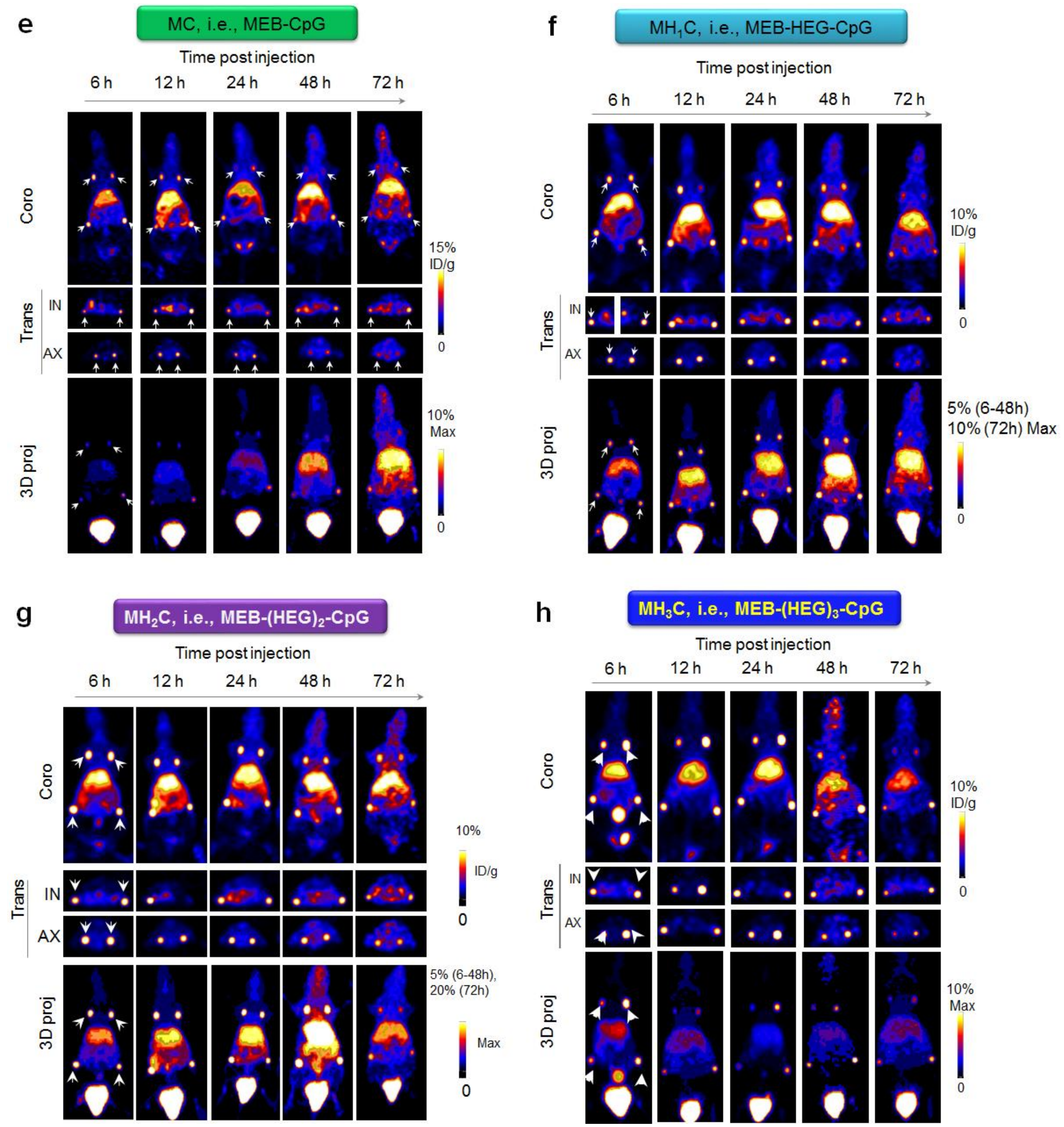

Supplementary Fig. 11. Quantitative screening AlbiCpG for optimal LN delivery. (a) Schematic illustration of the injection sites (base of tails) of radiolabeled CpG derivatives and the anatomical positions of inguinal (IN) LNs and axillary (AX) LNs. (b-h) Quantitative PET pharmaco-imaging of CpG, CpG-PEG20k, IFA-emulsified CpG, and MEB-CpG derivatives for LN-targeted delivery. Cu^{64} was labeled on CpG or derivatives for PET. Shown are the representative coronal (coro), transverse (trans), and 3D projection (3D proj) PET images of mice imaged at 6, 12, 24, 48, and 72 h post s.c. injection of the corresponding formulation of CpG at the base of the tail of FVB mice. The slightly reduced LN retention of PEG-CpG over free CpG is presumably because PEG interfered with the interaction between CpG and TLR9, thus affecting uptake by APCs and affecting LN

retention. The highlighted right insets in (c, d) verified the inefficient LN-targeted PEG-CpG and IFA(CpG) delivery, in which the radioactivity in LNs was still weak even with the scale bar as low as 0-1%ID/g. White arrow heads mark IN and AX LNs. Note that the scale bars are different for different formulations for best display of radioactivity in LNs. Injection dose: 4.4 - 5.5 Mbq. The scale bar for 3D proj was based on the maximal signal intensity in *each* image.

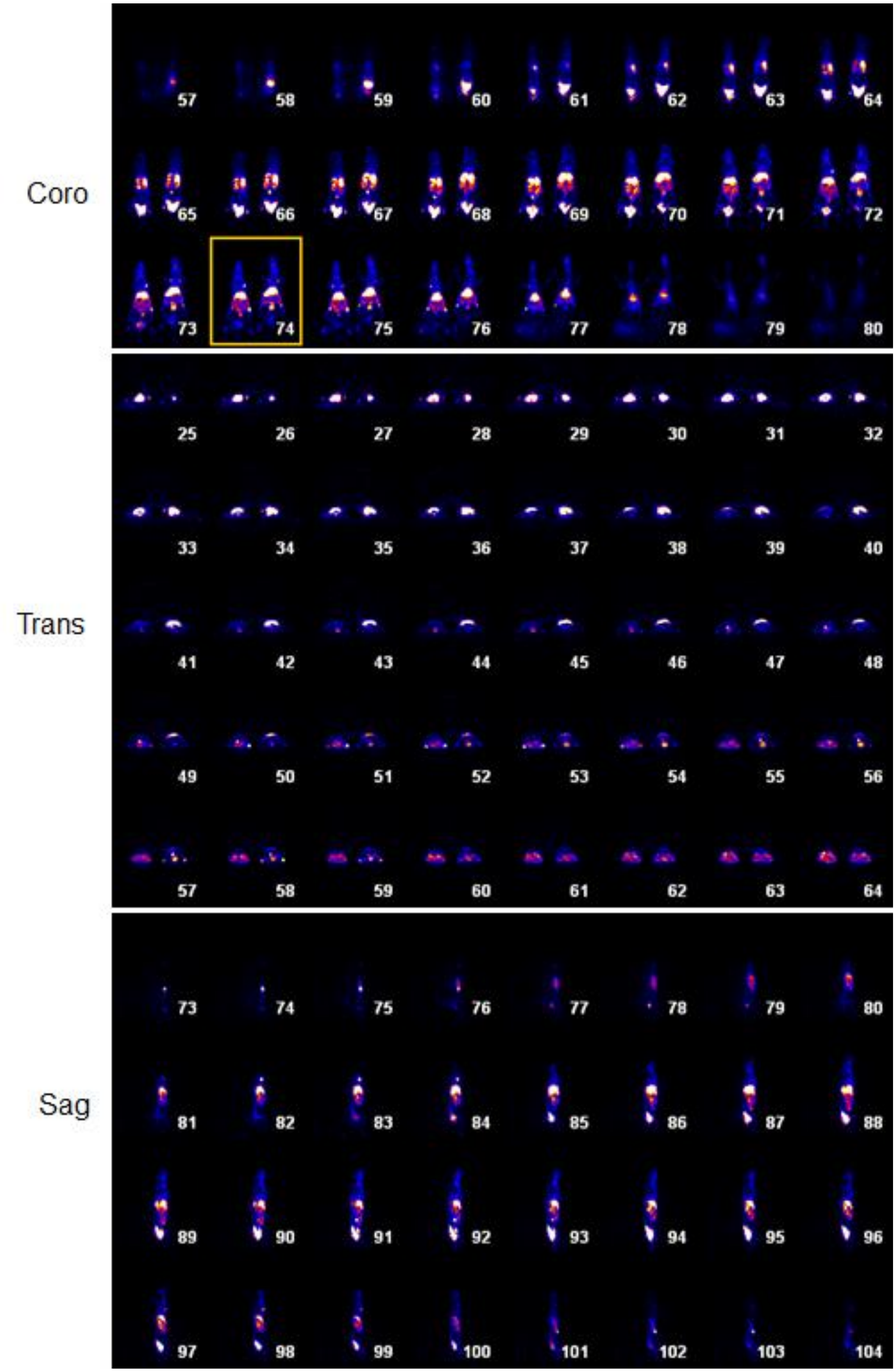

Supplementary Fig. 12. Consecutive series of coronal (coro), transverse (trans), and sagittal (sag) PET images of 2 FVB mice imaged at 6 h post s.c. injection of MEB-CpG at the tail base.

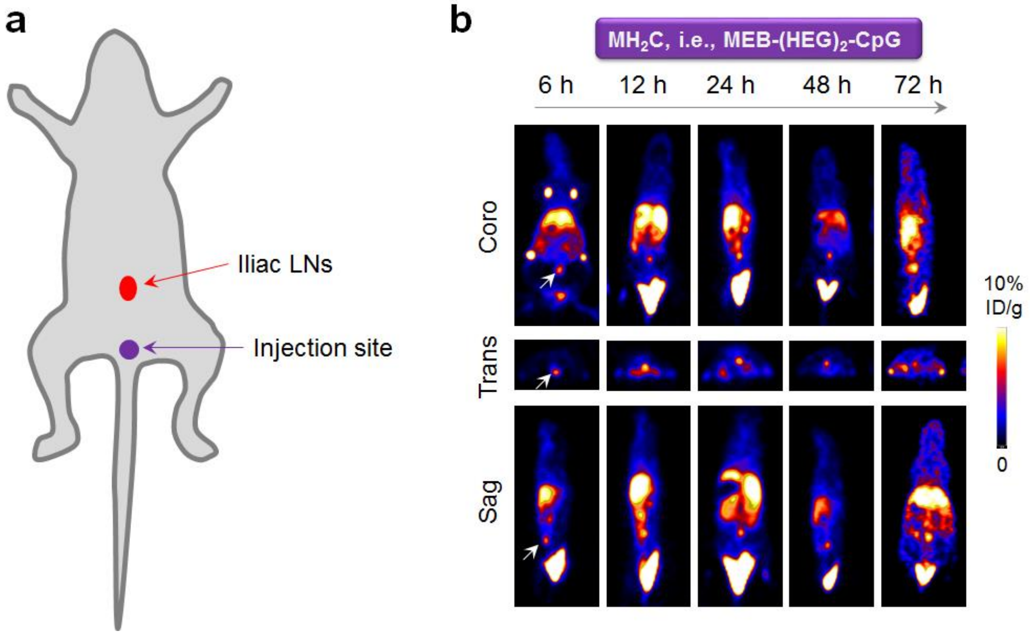

Supplementary Fig. 13. PET imaging also allowed noninvasive and sensitive detection of Cu^{64}-labeled CpG derivatives in deep draining LNs. (a) Schematic illustration of the injection site and anatomical position of iliac LNs. (b) Coronal (coro), transverse (trans), and sagittal (sag) views of PET images of mice s.c. injected with Cu^{64}-labeled AlbiCpG [MEB-$(HEG)_2$-CpG], showing the accumulation of AlbiCpG in iliac LNs. In addition to quantifying vaccines in inguinal LNs and axillary LNs, PET also allowed the quantification of vaccines in iliac LNs, which can also be important in immune regulations. Note that it is technically challenging to monitor iliac LN delivery of vaccines by optical imaging and invasive to isolate iliac LNs for *ex vivo* optical imaging.

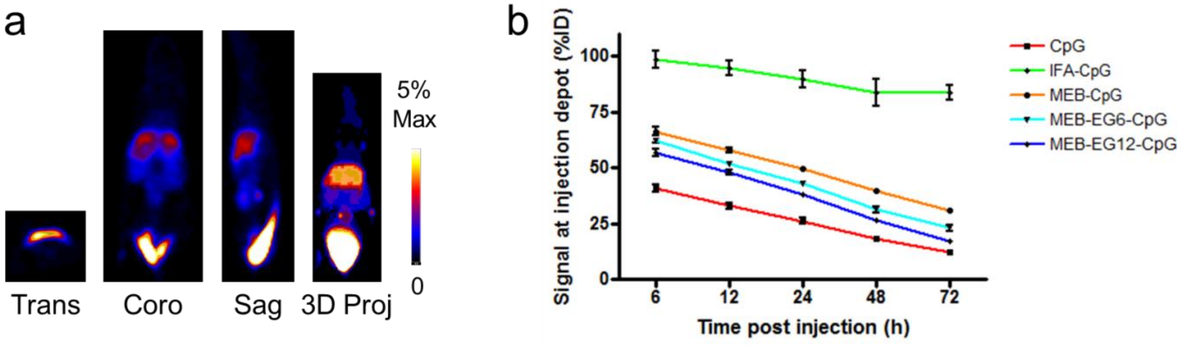

Supplementary Fig. 14. PET pharmacoimaging revealed AlbiVax at deep tissues and quantified vaccines at injection depots. (a) Coronal, transverse, and sagittal views of PET scanning of mice administered with Cu^{64}-labeled AlbiCpG (MEB-$(HEG)_2$-CpG), showing the retention of MEB-$(HEG)_2$-CpG in the injection depot and the gradual draining away from this depot. (b) Quantification of MEB-$(HEG)_2$-CpG retained in the injection depot over 3 days post administration.

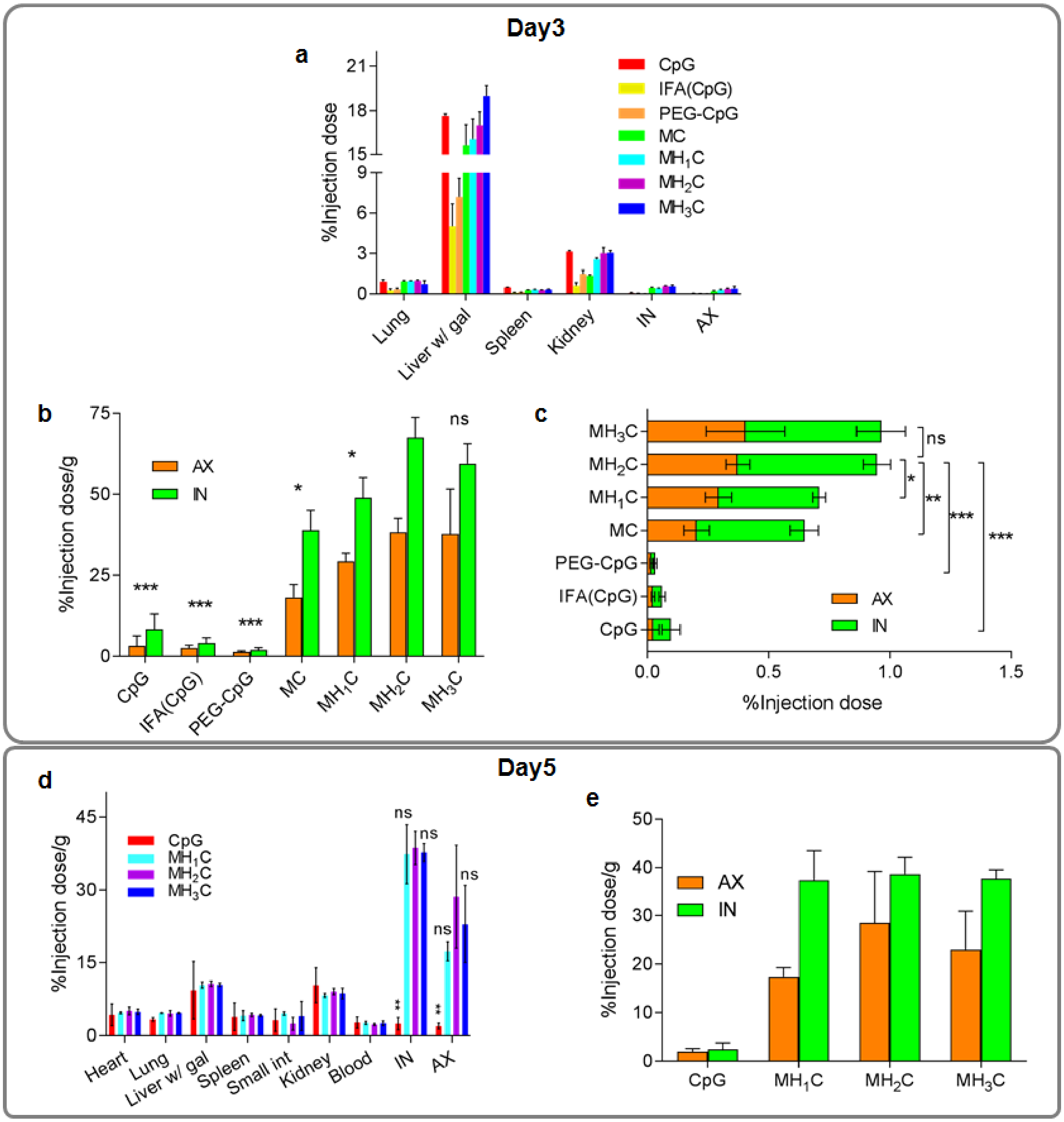

Supplementary Fig. 15. Biodistribution and LN retention of CpG on day3 and day5, as determined by ROI signal quantification of PET images and ɣ-counting of isolated organs. (a) Biodistribution of tested compounds in organs of interest measured by γ counting 3 days post injection (n = 4). (Liver/gal: liver with gallbladder; intest.: intestine; ID/g: ID/gram of organ weight). (b, c) ɣ-counting quantification of the %ID/g of different forms of CpG in LNs on Day3. Data are the same as those in (a). (d) ɣ-counting quantification of the %ID/g of different forms of CpG in major organs on Day5. (e) ɣ-counting quantification of the %ID/g of different forms of CpG in LNs on Day5. Due to signal decay over 5 days, the radioactivity was too low to be detected by PET on day5, so the %injection dose on Day5 was not determined by PET. ID: injection dose; ID/g: injection dose/gram of tissue.

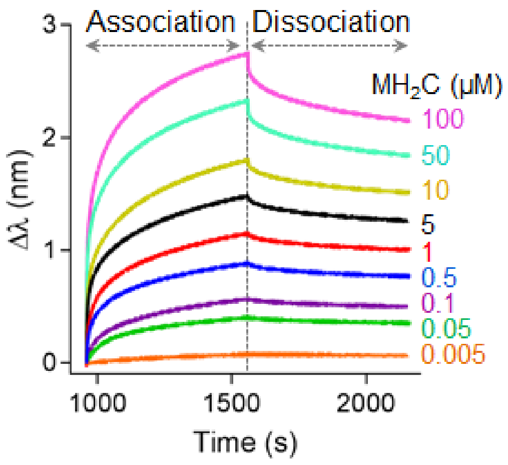

Supplementary Fig. 16. Binding kinetics of BSA and AlbiCpG in PBS measured by BLI.

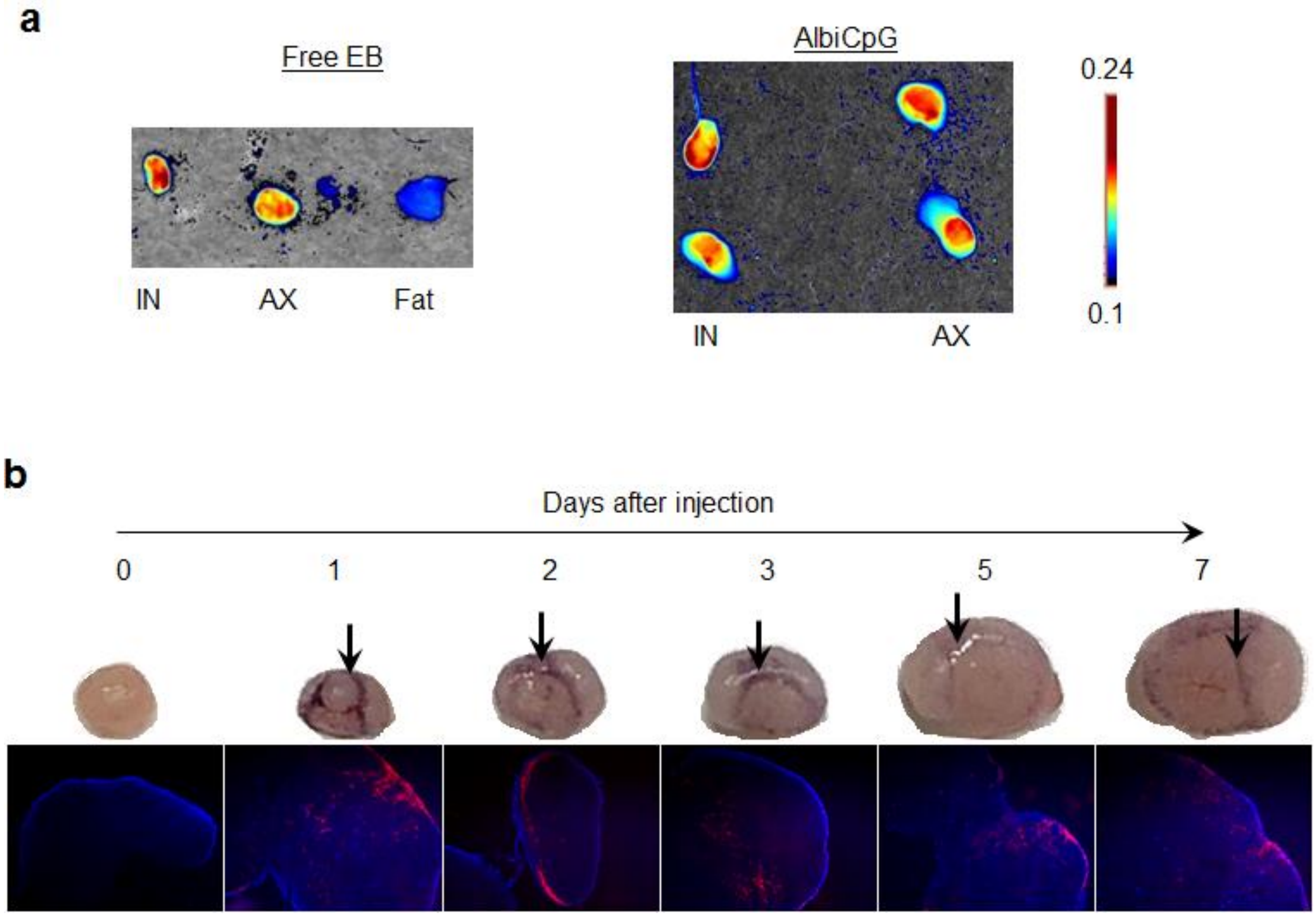

Supplementary Fig. 17. Observation of AlbiCpG delivery to LNs by MEB fluorescence and color. (a) Intrinsic fluorogenecity of MEB demonstrated targeted delivery of AlbiCpG into LNs. (b) AlbiCpG was retained in draining inguinal LNs for up to one week post injection. Shown above are the photographs of IN LNs collected on the corresponding days post injection, and at the bottom are the fluorescence images of LN tissue slices showing the distribution of AlbiCpG in LNs collected on the corresponding dates.

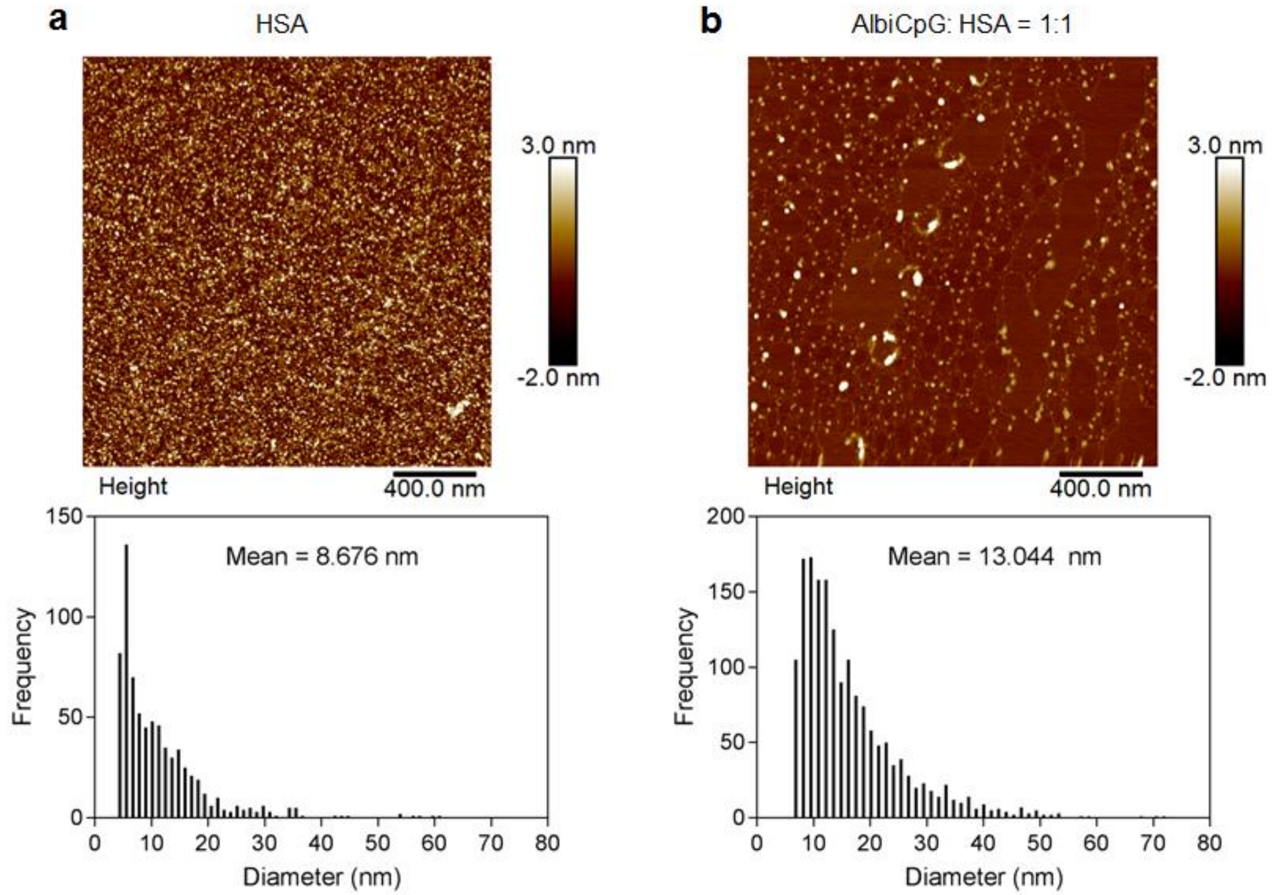

Supplementary Fig. 18. Sizes of albumin/AlbiVax nanocomplexes. (a-b) AFM images (top) and the size distribution (bottom) of HSA (a), and premixed HSA and AlbiCpG (AlbiCpG: HSA= 1:1) (b).

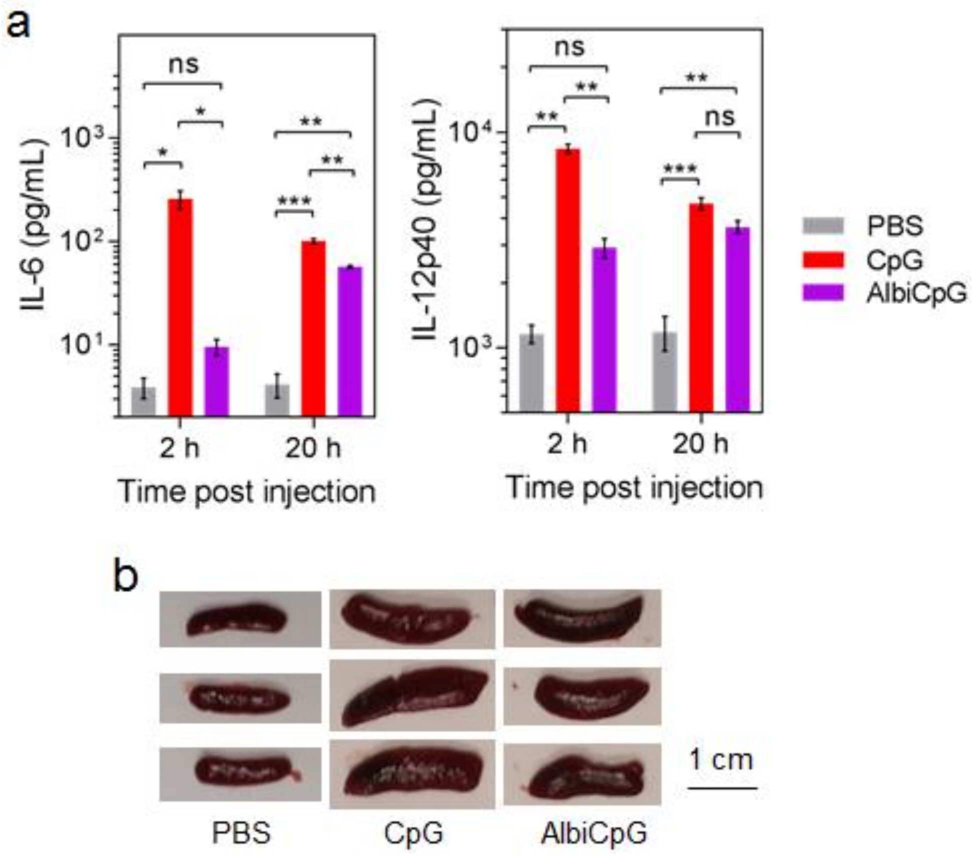

Supplementary Fig. 19. AlbiCpG ameliorated the acute systemic inflammation of CpG. (a) In C57BL/6 mice, AlbiCpG s.c. injected at the tail base ameliorated the systemic toxicity of CpG (n = 4) as shown by lower IL-6 and IL-12p40 titers in blood (e) (dose: 5 nmol CpG equivalents). (b) Photographs of spleens collected from mice injected with PBS, CpG, and AlbiCpG. Quantification of spleen/body weight was shown in Fig. 2. (Injection dose: 5 nmol on day0 and day3; spleens were collected on day6)

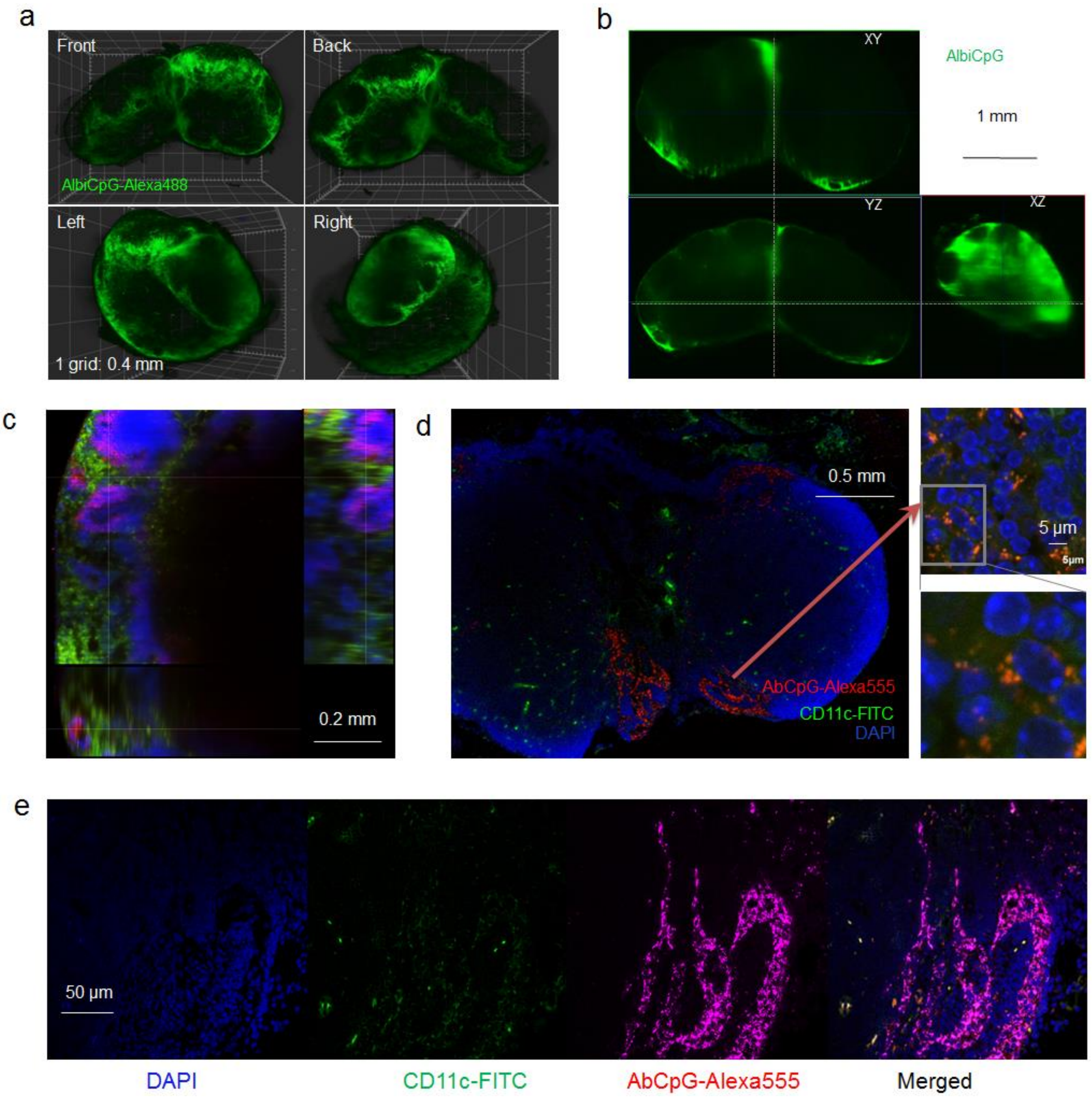

Supplementary Fig. 20. Fluorescence microscopy images showing intranodal distribution of AlbiCpG in draining inguinal LNs. (a-b) Light sheet fluorescence microscopy images that show the 3D views from different perspectives (a) and the cross-sections (b) showing the intranodal distribution of AlbiCpG-Alexa488 in cleared LNs. The sample is the same as shown in Fig. 3a-b. 1 grid: 400 µm. (c) 1 day after injection, a 3D cross-section view of cleared LNs showing the distribution of FITC-labeled AlbiCpG in draining LNs. The sample is the same as shown in Fig. 3c. In this LN where B cells ($B220^{+}$) and all nucleated cells ($DAPI^{+}$) were stained, abundant AlbiCpG was observed in subcapsular sinus areas and around B cell follicles. (d) Left: an epiluorescence microscopy image of a whole LN slide view, right: confocal microscopy images of the close-up view of the LN slide. (e) confocal microscopy images showing the localization of AlbiCpG in LNs using

draining IN LN slices, in individual channels. DCs were stained with anti-CD11c-FITC, and nucleated cells were stained with DAPI.

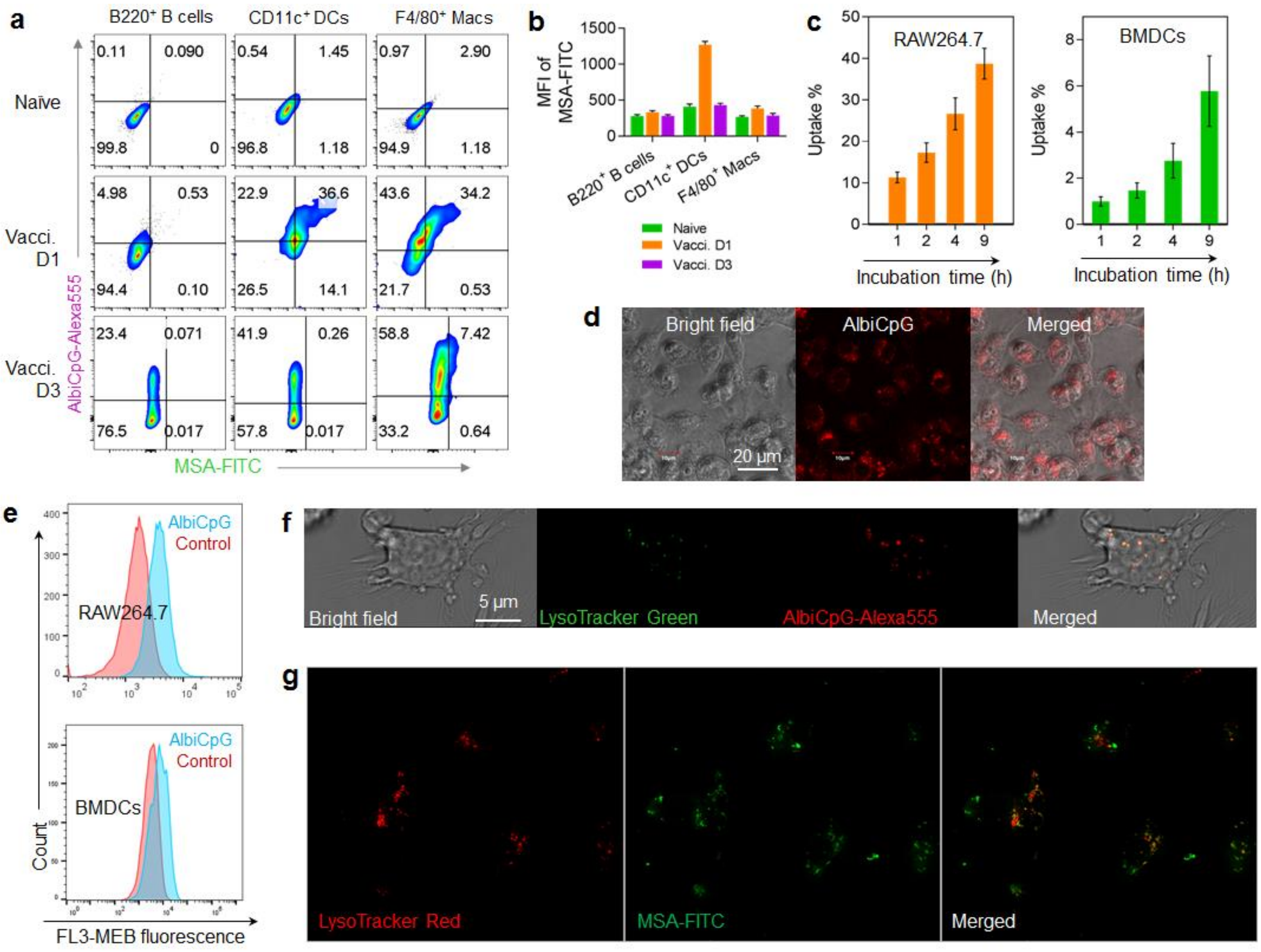

Supplementary Fig. 21. Intracellular delivery of albumin/AlbiCpG into APCs *in vivo* and *in vitro.* (a) Representative flow cytometry results showing the uptake of AlbiCpG-Alexa555 and/or MSA-FITC in B220$^+$ B cells, CD11c$^+$ DCs, and F4/80$^+$ macrophages, 1 day (D1) and 3 days (D3) post vaccination with premixed MSA-FITC/AlbiCpG-Alexa555. (b) Median fluorescence intensity (MFI) of MSA-FITC in B220$^+$ B cells, CD11c$^+$ DCs, and F4/80$^+$ macrophages. The MFI of AlbiCpG will be discussed below. (c-e) Efficient *in vitro* uptake of AlbiCpG into BMDCs and RAW264.7 macrophages was demonstrated by quantitative γ-counting using ^{64}Cu-labeled AlbiCpG (c), confocal microscopy (d), and flow cytometry using MEB fluorescence (e). (f) Same study as shown in Fig. 3e showing confocal microscopy of a single BMDC after deconvolution, with individual and merged fluorescence/bright-field channels. (g) Super-resolution instant SIM images showing efficient uptake of FITC-labeled MSA into BMDCs. MSA was located in the endolysosome after incubation with BMDCs for 3 h.

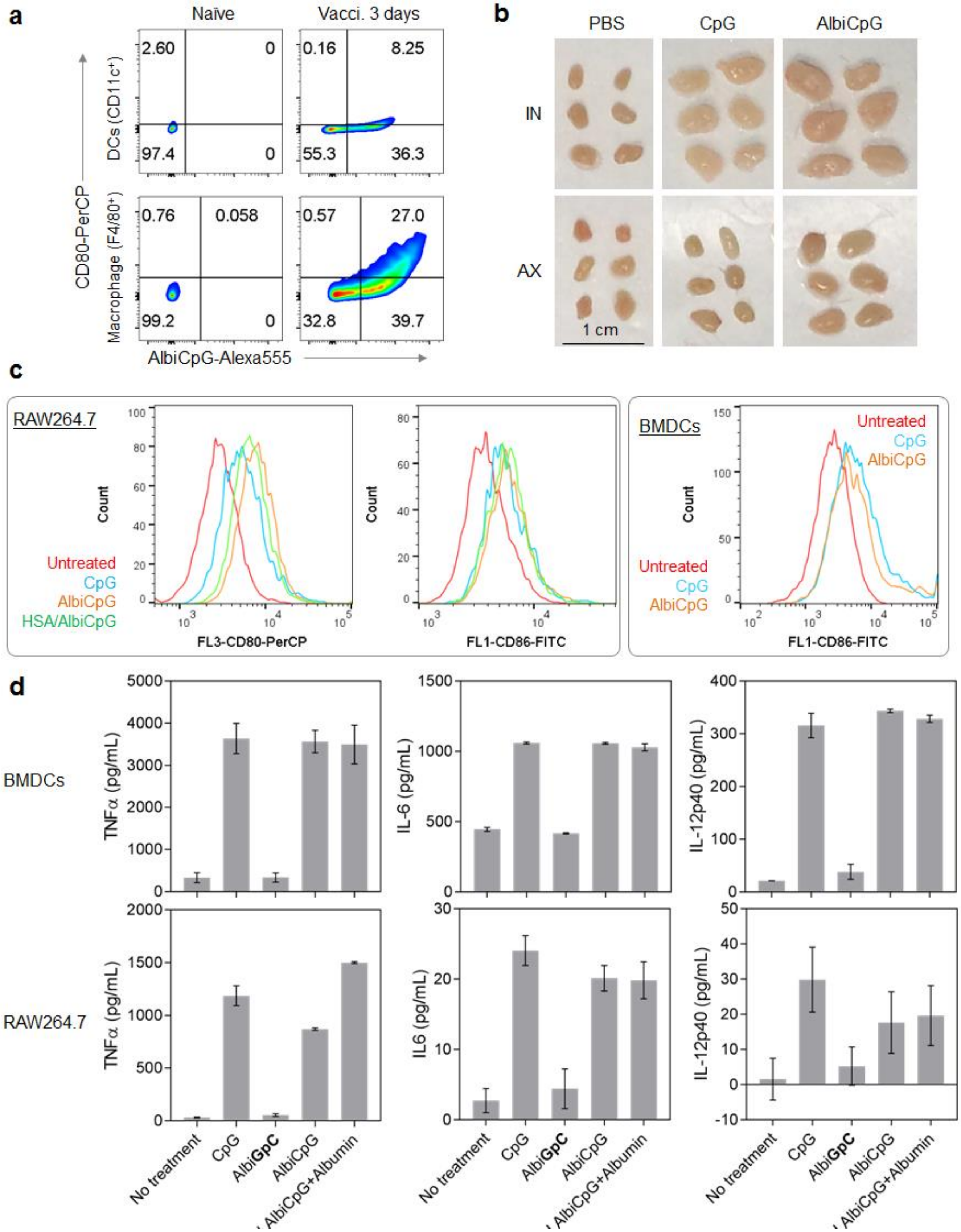
a
Naïve
Vacci. 3 days
CD80-PerCP
DCs (CD11c+)
Macrophage (F4/80+)
AlbiCpG-Alexa555
2.60
0
97.4
0
0.16
8.25
55.3
36.3
0.76
0.058
99.2
0
0.57
27.0
32.8
39.7
b
PBS
CpG
AlbiCpG
IN
AX
1 cm
c
RAW264.7
Count
Untreated
CpG
AlbiCpG
HSA/AlbiCpG
FL3-CD80-PerCP
FL1-CD86-FITC
BMDCs
FL1-CD86-FITC
d
BMDCs
RAW264.7
TNFα (pg/mL)
IL-6 (pg/mL)
IL-12p40 (pg/mL)
IL6 (pg/mL)
No treatment
CpG
AlbiGpC
AlbiCpG
AlbiCpG+Albumin

Supplementary Fig. 22. *In vitro* and *in vivo* immunostimulation of AlbiCpG. (a) Representative flow cytometry plots showing the up-regulated expression of CD80 in DCs and macrophages of draining IN LNs. C57BL/6 mice were s.c. injected with AlbiCpG-Alexa555, and LN-residing APCs were analyzed on day3 post injection. (b) AlbiCpG induced local lymphadenopathy, in which LNs swelled resulting from the buildup of lymph and cells in LNs. C57BL/6 mice were s.c. injected at the base of the tail (dose: 5 nmol/mouse), on day0 and day3, and organs were collected on day6. (c) *In vitro* immunostimulation of AlbiCpG in APCs was also demonstrated by up-regulated expression of co-stimulatory factors CD86 and/or CD80 in RAW264.7 and BMDCs (concentrations: 100 nM; treatment time: 14 h). (d) ELISA analysis of the culture medium of *in vitro* cells demonstrate that AlbiCpG, either alone or premixed with albumin, stimulated BMDCs and RAW264.7 cells to produce proinflammatory factors at comparable levels as free CpG. (Treatment time: 14 h; concentrations: 100 nM for TNFα in BMDCs and IL-6 and IL-12p40 in RAW264.7, 20 nM for IL-6 and IL-12p40 in BMDCs, and 10 nM for TNFα in RAW264.7 macrophage.)

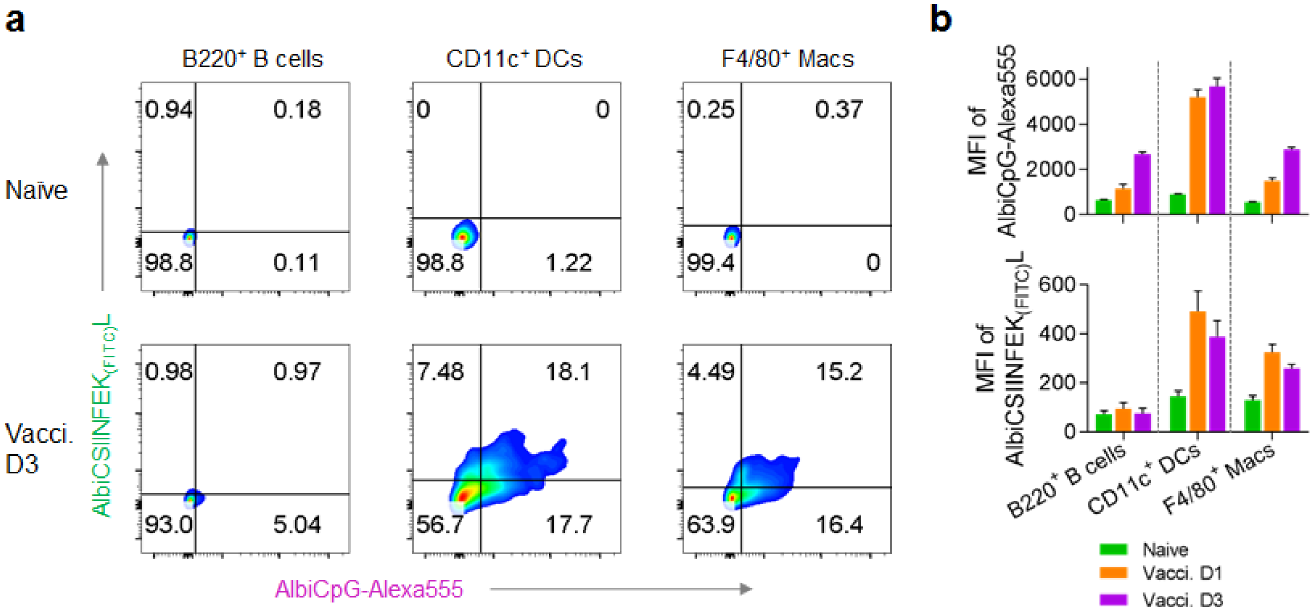

Supplementary Fig. 23. Additional data in Fig. 3g showing co-delivery of AlbiCpG and AlbiAg. Representative flow cytometry plots (a) and MFI quantified from flow cytometry data (b) showing the double-positive adjuvant and antigen (AlbiCpG$^+$AlbiCSIINFEKL$^+$) in DCs and macrophages of draining IN LNs. C57BL/6 mice were s.c. injected with AlbiCpG-Alexa555 and AlbiCSIINFEK$_{(FITC)}$L, followed by flow cytometric analysis of LN-residing APCs.

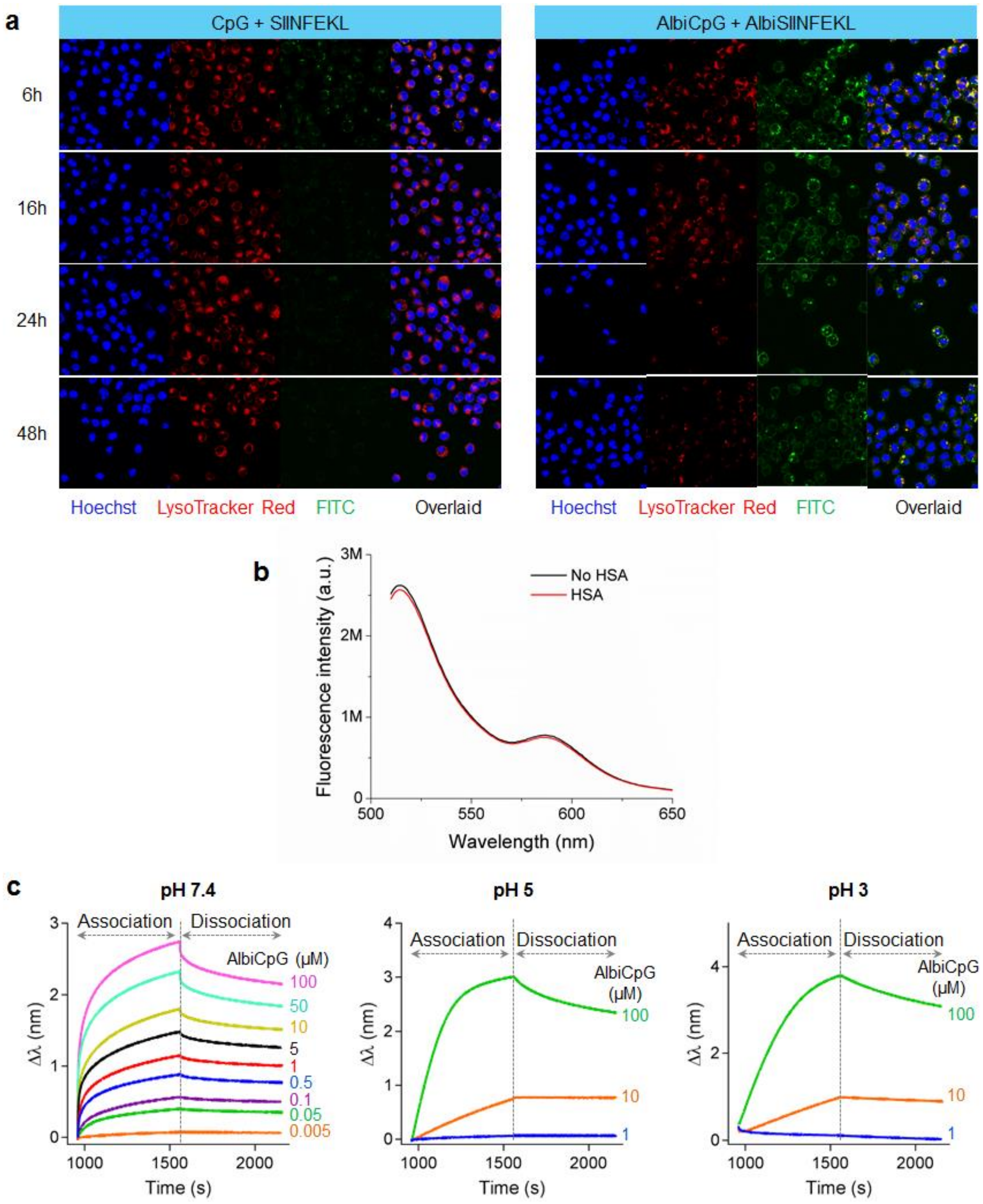

Supplementary Fig. 24. Efficient antigen presentation mediated by AlbiVax. (a) Additional confocal microscopy images from Fig. 3i. Confocal microscopy images showing efficient antigen presentation of AlbiCSIINFEK$_{(FITC)}$L (500 nM) + AlbiCpG (500 nM), compared with CSIINFEK$_{(FITC)}$L (500 nM) + CpG (500 nM) in BMDCs. For incubation time >6 h, fresh medium was substituted to cells incubated with the above vaccines after for 14-h incubation to mimic *in vivo* vaccine pharmacokinetics. (b) Fluorescence spectra suggested that the intracellular co-delivery of CpG and SIINFEKL was likely mediated by binding of AlbiCpG and

AlbiCSIINFEKL with separate albumin molecules, because Förster resonance energy transfer between AlbiCpG-Alexa555 and AlbiCSIINFEK$_{(FITC)}$L was undetectable in the presence of albumin. On the other hand, if AlbiCpG-Alexa555 and AlbiCSIINFEK$_{(FITC)}$L bind to the same albumin molecule, the proximity between AlbiCpG-Alexa555 and AlbiCSIINFEK$_{(FITC)}$L would have resulted in FRET. Concentrations: AlbiCpG-Alexa555: 50 nM; AlbiCSIINFEK$_{(FITC)}$L: 300 nM; HAS: 50 nM. Excitation: 488 nm; emission: 510 – 650 nm. (c) BLI binding kinetics of AlbiCpG with BSA at pH 7.4 (K_d = 1.0 µM) (same as Supplemental Fig. 16), pH 5 (K_d = 26.7 µM), and pH 3 (K_d = 29.4 µM). The binding ability was significantly reduced by over 20 folds under acidic conditions.

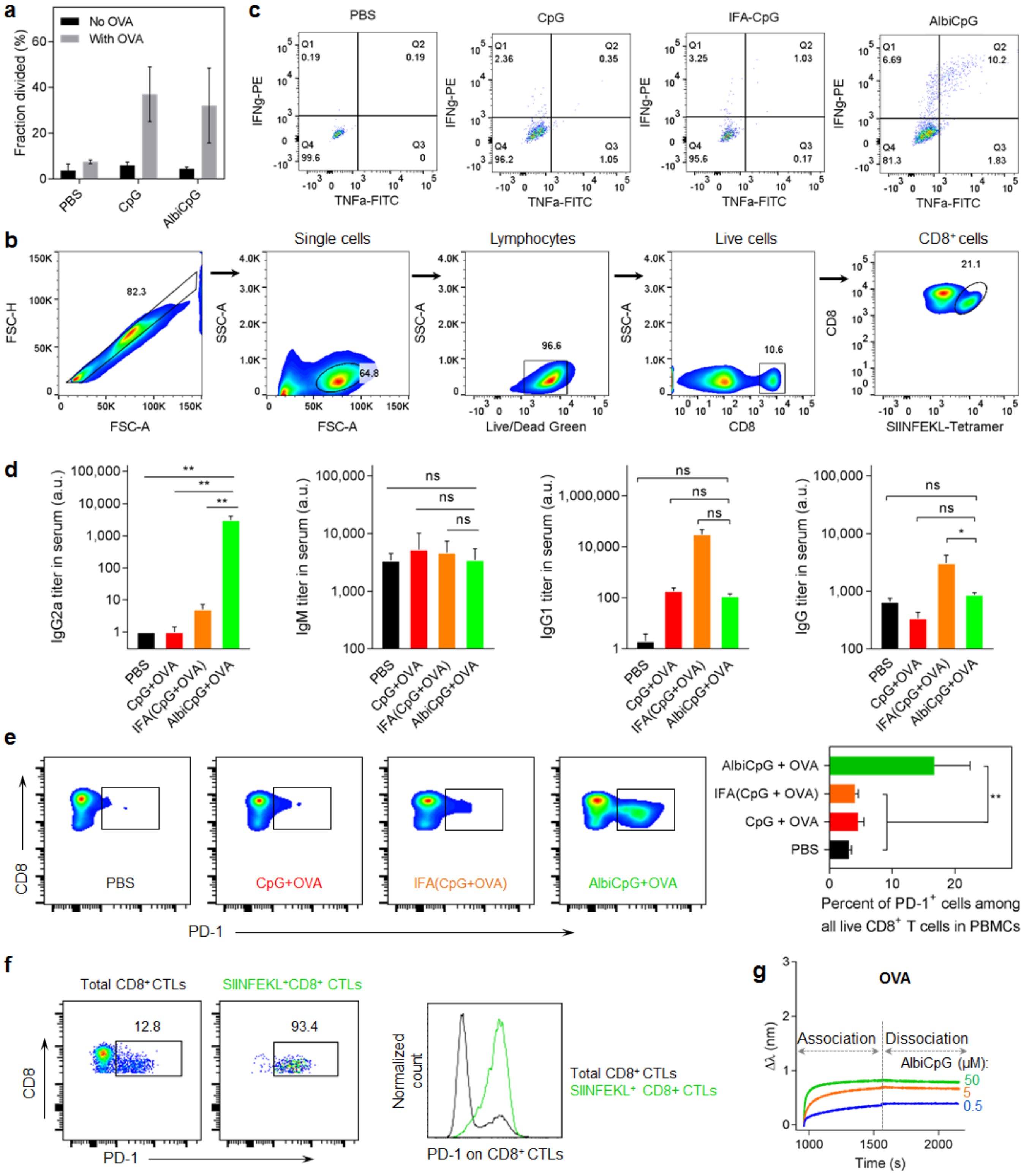

Supplementary Fig. 25. AlbiVax (AlbiCpG + OVA) induced potent antitumor immunity including T cell responses. (a) AlbiCpG + OVA stimulated *in vitro* proliferation of OVA-specific CD8$^+$ T cells from OT-1 mice, at a comparable level as CpG. (b-f) AlbiCpG + OVA) induced potent antitumor immunity. C57BL/6 mice were vaccinated on day0 and day14 (2 nmol CpG equivalents and 10 µg OVA), and peripheral PBMCs and serum were analyzed on day21. (b) Gating tree used in flow cytometric analysis of tetramer staining in PBMCs. (c)

Representative dot plots showing that AlbiVax enhanced cytotoxicity of antigen-specific CD8⁺ T cells. (d) AlbiVax (AlbiCpG + OVA) induced potent production of antigen-specific antibodies. C57BL/6 mice (*n* = 4-5) were s.c. vaccinated at the tail base on day0 and day14 with AlbiVax (2 nmol CpG equivalents, 10 µg OVA), followed by analysis of OVA-specific antibody titers on day21. Shown are serum titers of IgG2a, IgM, IgG and IgG1 in vaccinated mice. AlbiCpG was superior in the production of IgG2a, which is effective for tumor therapy. IFA-emulsified CpG + OVA [IFA(CpG + OVA)] was superior to AlbiCpG in the overall IgG and IgG1 response. Note the log scale of both mean and s.e.m. values. (e-f) AlbiCpG + OVA elicited the upregulation of PD-1 expression on antigen-specific $CD8^+$ CTLs. (e) Representative flow cytometry plots (left) and frequency (right) of PD-1-positive cells among live $CD8^+$ CTLs in peripheral blood. (f) Representative flow cytometry plots showing the frequencies (left) and fluorescence intensity histogram (right) of PD-1 expression of $CD8^+$ CTLs among total $CD8^+$ CTLs and SIINFEKL-specific $CD8^+$ CTLs, upon vaccination with AlbiCpG + OVA. (g) BLI binding kinetics of AlbiCpG with OVA at pH7.4 (K_d = 1.0 µM). The binding signal was weaker than AlbiCpG to MSA (Fig. 2f), but likely still contributed to the robust immunity induced by AlbiCpG + OVA. Data represent mean ± SD. **$p < 0.01$, *$p < 0.05$, ns: not significant ($p > 0.05$), by one-way ANOVA with Bonferroni post-test.

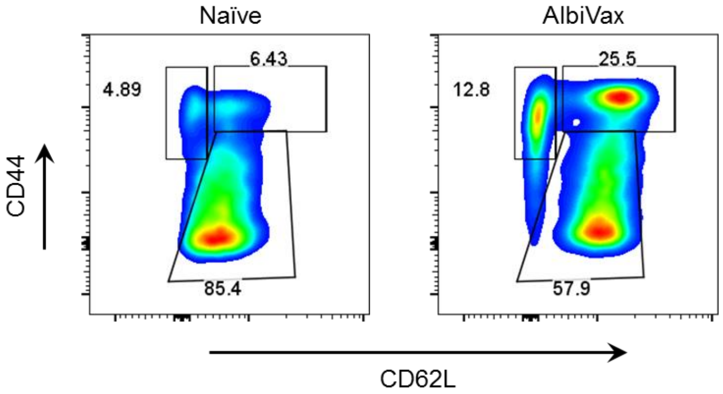

Supplementary Fig. 26. Representative flow cytometry results showing the analysis of immune memory CD8⁺ T cells among total CD8⁺ T cells of native or AlbiVax-immunized mice.

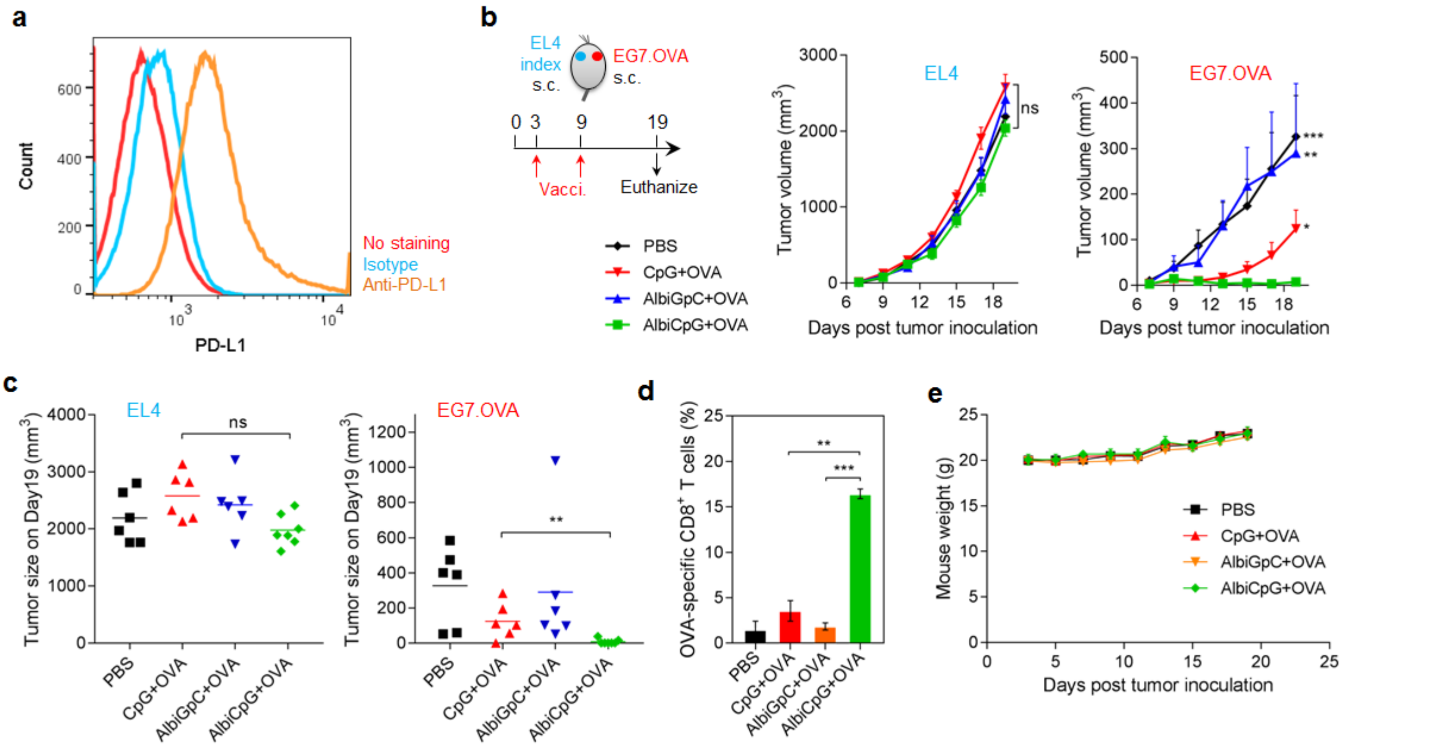

Supplementary Fig. 27. AlbiVax-based cancer immunotherapy. (a) PD-L1 expression level on EG7.OVA tumor cells. (b) Experiment outline (left): C57BL/6 mice (n = 5-7) were inoculated with 3 x 10^5 EL4 cells on the left shoulder and 3 x 10^5 EG7.OVA cells on the right shoulder on day0, treated with AlbiVax or controls (2 nmol CpG, 20 µg OVA) on day3 and day9. Right: growth curves of EL4 tumors and EG7.OVA tumors. (c) Tumor sizes of EL4 and EG7.OVA on day19 post tumor inoculation. (d) Percentage of peripheral SIINFEKL-specific $CD8^+$ T cells in mice, as determined by staining peripheral blood cells with SIINFEKL-H-$2K^b$ tetramer on day19 post tumor inoculation. (e) Mouse weight monitored over the course of treatment. No significant mouse weight change or morbidity was observed during the treatment. ***P < 0.001, **P < 0.01, *P < 0.05, ns: not significant (P > 0.05), by one-way ANOVA with Bonferroni post-test, unless denoted otherwise. Data are shown as mean ± s.e.m.. Asterisks in (b) indicate statistically significant differences between AlbiCpG and other groups.

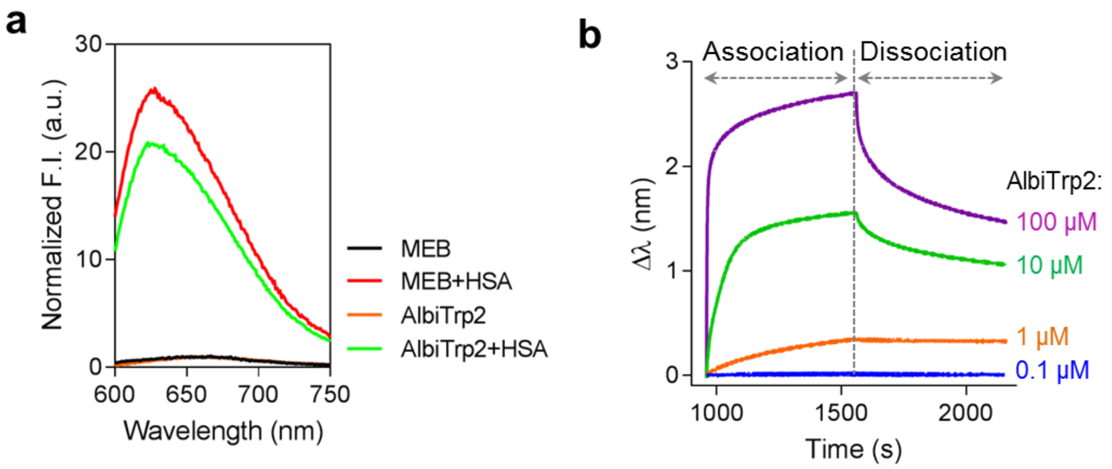

Supplementary Fig. 28. Trp2-based AlbiVax. (a) Albumin binding of AlbiTrp2 recovered the MEB fluorescence. (b) BLI binding kinetics of BSA (1 μg/mL) with AlbiTrp2 in PBS (pH 7.4).

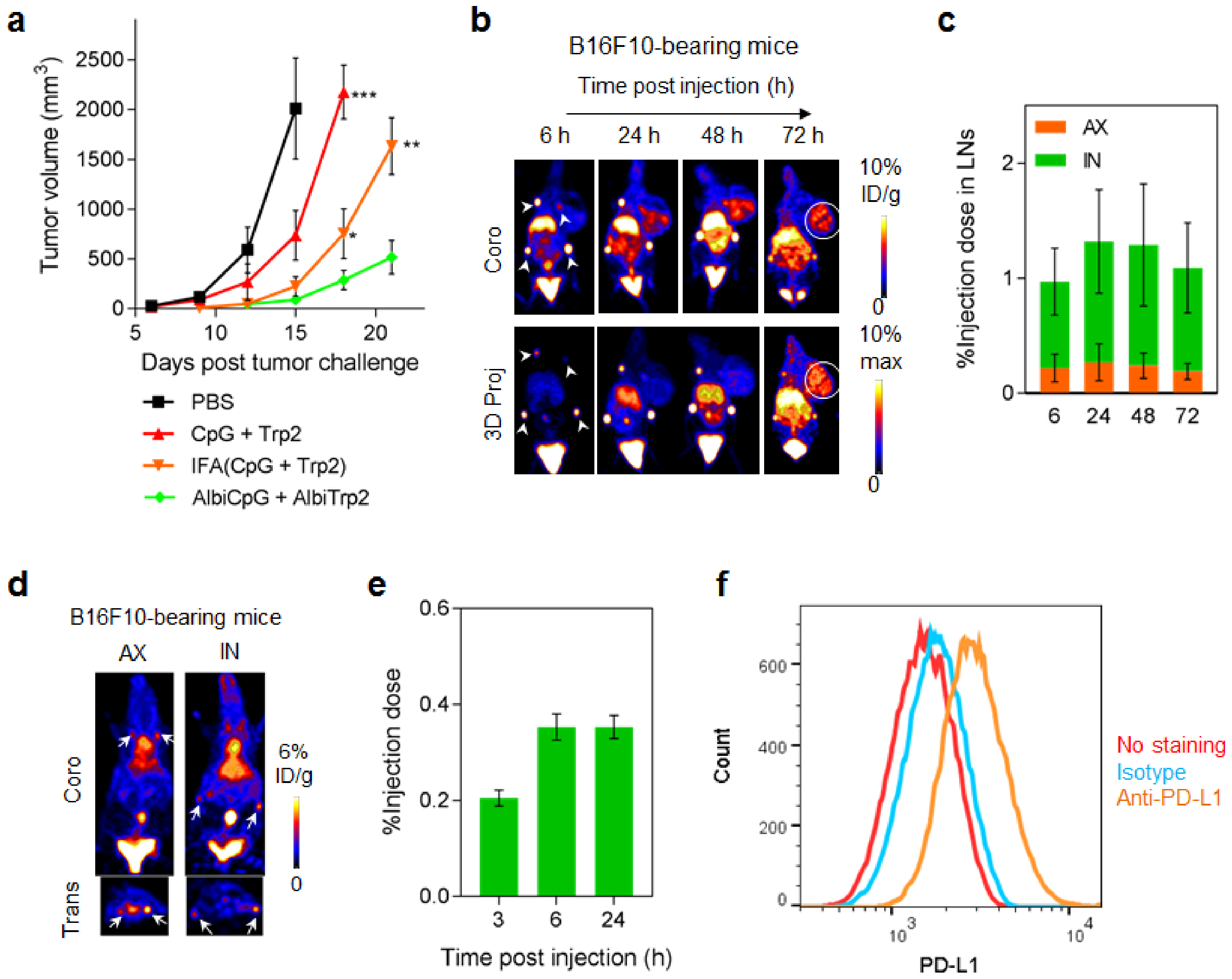

Supplementary Fig. 29. AlbiVax (AlbiCpG + AlbiTrp2) for melanoma immunotherapy. (a) Growth curves of B16F10 tumors in mice that were prophylactically immunized with AlbiVax. 3 x 10^5 B16F10 melanoma cells were s.c. injected. (b-c) Representative PET images (b) and the corresponding quantification (c) showing the uptake of AlbiCpG (MEB-$(HEG)_2$-CpG) in draining IN LNs and AX LNs of B16F10 tumor-bearing C57BL/6 mice. Tumor-draining LNs, the AX LNs on the right of mouse body, showed low vaccine signal due to blocked lymphatic drainage in the large tumor tissues. Injection dose: 4.4 - 5.5 Mbq. (d-g) Representative PET images (d) and the corresponding quantification (e) showing the uptake of AlbiTrp2 in LNs (IN and AX) of B16F10 tumor-bearing C57BL/6 mice. (Coro: coronal; trans: transverse; proj: projection; ID: injection dose; ID/g: ID/gram of organ weight) (f) Flow cytometry result showing the PD-L1 expression level on B16F10 tumor cells.

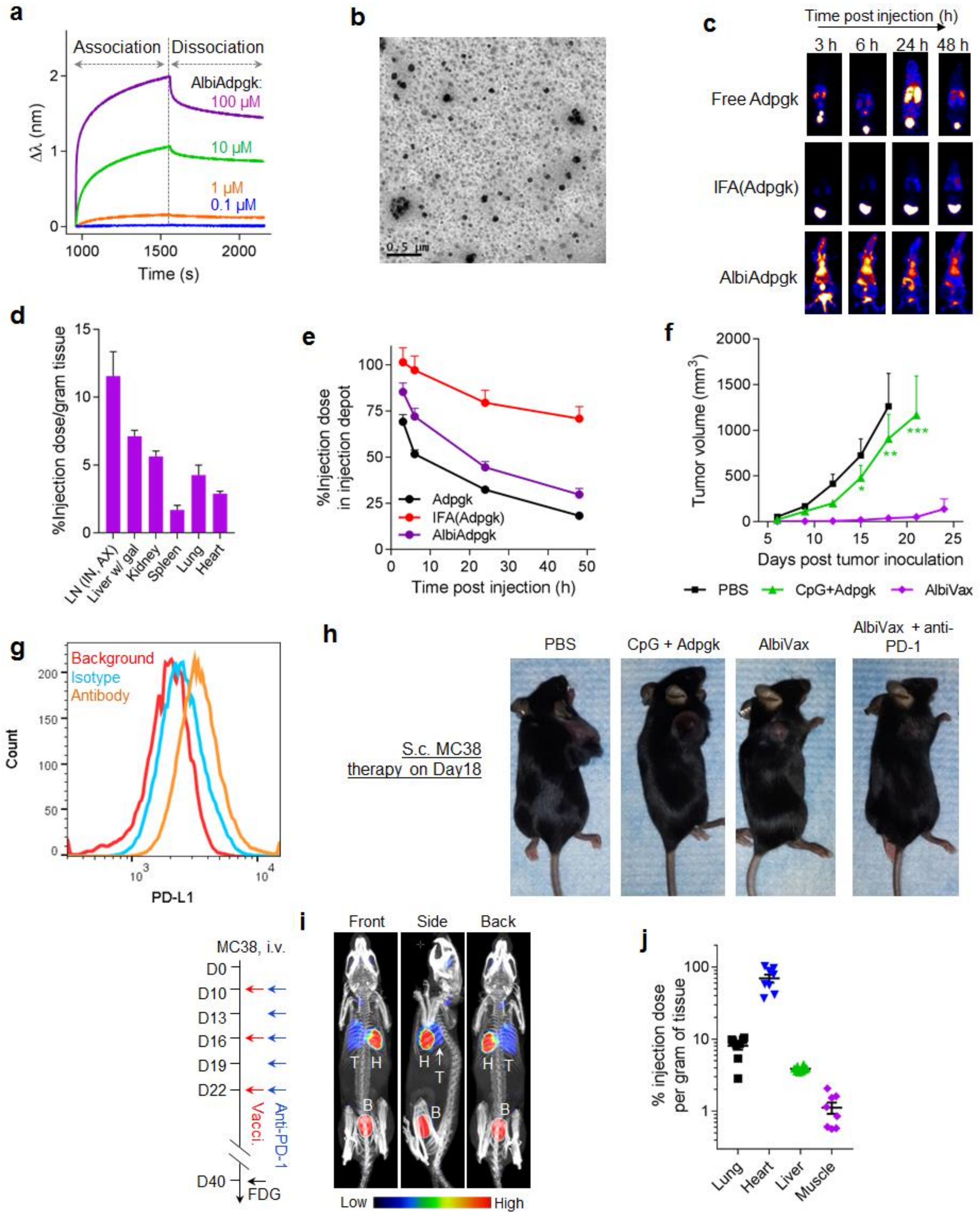

Supplementary Fig. 30. Neoantigen-based AlbiVax for personalized tumor immunotherapy. (a) BLI binding kinetics of BSA (1 μg/mL) with AlbiAdpgk in PBS (pH7.4). (b) A TEM image of AlbiAdpgk nanoparticles in water. Scale bar: 500 nm. (c-e) Pharmacoimaging of AlbiAdpgk in FVB mice. (c) Representative PET images of 64Cu-labeled AlbiAdpgk, free Adpgk, and IFA(Adpgk). Injection dose: 4.4 - 5.5 Mbq. (d) Quantified biodistribution of ^{64}Cu-labeled AlbiAdpgk in organs by γ-counting of excised organs at 48 h post

injection. (e) %ID at injection sites quantified from decay-corrected PET scans. (f) MC38 tumor growth curves after challenging AlbiVax-immunized C57BL/6 mice with 3 x 10^5 MC38 tumor cells. (g) PD-L1 expression level in MC38 tumor cells. (h) Representative photographs of mice undertaking immunotherapy at day18 post tumor inoculation. (i-j) Immunotherapy of lung metastatic MC38 tumor with AlbiCpG + AlbiAdpgk alone or in combination with anti-PD-1. C57BL/6 mice were *i.v*. inoculated with 1 x 10^5 MC38 cells, treated with AlbiCpG (2 nmol) + AlbiAdpgk (20 µg) on days 10, 16, and 22), and with anti-PD-1 (200 µg) every 3 days from day10 for 6 times. On day40, mice were injected with FDG tracer (3.7 Mbq). (i) Lung metastatic tumors were shown by PET/CT scanning of randomly selected mice treated with PBS. T: tumor; H: heart; B: bladder. (j) Mice were euthanized and organs of interest were resected and radioactivity measured by γ-counting. Data show mean ± s.e.m. of 2 – 3 independent experiments.

Reference

1 Liu, Y. *et al.* Stable Evans Blue Derived Exendin-4 Peptide for Type 2 Diabetes Treatment. *Bioconjugate Chemistry* **27**, 54-58, doi:10.1021/acs.bioconjchem.5b00625 (2016).
