# Supplementary material for: Albumin/vaccine nanocomplexes that assemble in vivo for combination cancer immunotherapy
Source: Nat Commun. 2017 Dec 5;8:1954. doi: 10.1038/s41467-017-02191-y (PMC5715147; doi:10.1038/s41467-017-02191-y)
Supplement: Supplementary file 7 — Peer Review File [file 41467_2017_2191_MOESM7_ESM.pdf]

Editorial Note: this manuscript has been previously reviewed at another journal that is not operating a transparent peer review scheme. This document only contains reviewer comments and rebuttal letters for versions considered at Nature Communications.

Reviewer #1 (Remarks to the Author):

The authors have addressed my concerns sufficiently in the revised manuscript.

Reviewer #2 (Remarks to the Author):

The authors have addressed my concerns.

Reviewer #3 (Remarks to the Author):

All my comments were adequately addressed, no more concerns.

Responses to comments

-- “Albumin/Vaccine Nanocomplexes That Assemble In Vivo for Combination Cancer Immunotherapy”

(manuscript NCOMMS-17-24287-T), submitted to Nature Communications

REVIEWERS' COMMENTS:

REVIEWERS' COMMENTS:

Reviewer #1 (Remarks to the Author):

The authors have addressed my concerns sufficiently in the revised manuscript.

Response: No revision needed.

Reviewer #2 (Remarks to the Author):

The authors have addressed my concerns.

Response: No revision needed.

Reviewer #3 (Remarks to the Author):

All my comments were adequately addressed, no more concerns.

Response: No revision needed.
